# Supplementary material for: Anti-Adipogenic Lanostane-Type Triterpenoids from the Edible and Medicinal Mushroom Ganoderma applanatum
Source: J Fungi (Basel). 2022 Mar 22;8(4):331. doi: 10.3390/jof8040331 (PMC9028577; doi:10.3390/jof8040331)
Supplement: Supplementary file 1 [file jof-08-00331-s001.zip › supplementary material.pdf]

Anti-Adipogenic Lanostane-Type Triterpenoids from the Edible and Medicinal Mushroom *Ganoderma applanatum*

Xing-Rong Peng,[1,2,#] Qian Wang,[3,#] Hai-Guo Su,[1,2] Lin Zhou,[1,2] Wen-Yong Xiong,[1,3,*] and Ming-Hua Qiu[1,2,*]

1 State Key Laboratory of Phytochemistry and Plant Resources in West China, Kunming Institute of Botany, Chinese Academy of Science, Kunming 650201, People's Republic of China

2 University of the Chinese Academy of Science, Beijing 100049, People's Republic of China

3 Key Laboratory of Medicinal Chemistry for Natural Resource, Ministry of Education; Yunnan Provincial Center for Research & Development of Natural Products; School of Chemical Science and Technology, Yunnan University, Kunming 650091, P.R.China.

* Correspondence: pengxingrong@mail.kib.ac.cn (X.-R.P); xwy@ynu.edu.cn (W.-Y.X); mhchiu@mail.kib.ac.cn (M.-H.Q)

[#] These authors equally contribute to this paper.

Contents

Section S1: 1D and 2D NMR spectra of compound 1 7

Figure S1. ^{1}H NMR (600 MHz, MeOH-d_4) spectrum of 1. 7

Figure S2. ^{13}C NMR (150 MHz, MeOH-d_4) spectrum of 1. 8

Figure S3. ^{1}H-^{1}H COSY (600 MHz, MeOH-d_4) spectrum of 1. 9

Figure S4. HSQC (600/150 MHz, MeOH-d_4) spectrum of 1. 10

Figure S5. HMBC (600/150 MHz, MeOH-d_4) spectrum of 1. 11

Figure S6. ROESY (600/150 MHz, MeOH-d_4) spectrum of 1. 12

Section S2: HRESIMS spectrum of 1 13

Section S3: X-ray crystallographic data of 1 14

Section S4: 1D and 2D NMR spectra of compound 2 16

Figure S8. ^{1}H NMR (600 MHz, MeOH-d_4) spectrum of 2. 16

Figure S9. ^{13}C NMR (150 MHz, MeOH-d_4) spectrum of 2. 17

Figure S10. ^{1}H-^{1}H COSY (600 MHz, MeOH-d_4) spectrum of 2. 18

Figure S11. HSQC (600/150 MHz, MeOH-d_4) spectrum of 2. 19

Figure S12. HMBC (600/150 MHz, MeOH-d_4) spectrum of 2. 20

Figure S13. ROESY (600/150 MHz, MeOH-d_4) spectrum of 2. 21

Section S5: HRESIMS spectrum of 2 22

Section S6: 1D and 2D NMR spectra of compound 3 23

Figure S15. ^{1}H NMR (600 MHz, MeOH-d_4) spectrum of 3. 23

Figure S16. ^{13}C NMR (150 MHz, MeOH-d_4) spectrum of 3. 24

Figure S17. ^{1}H-^{1}H COSY (600 MHz, MeOH-d_4) spectrum of 3. 25

Figure S18. HSQC (600/150 MHz, MeOH-d_4) spectrum of 3. 26

Figure S19. HMBC (600/150 MHz, MeOH-d_4) spectrum of 3. 27

Figure S20. ROESY (600/150 MHz, MeOH-d_4) spectrum of 3. 28

Section S7: HRESIMS spectrum of 3 29

Section S8: 1D and 2D NMR spectra of compound 4 30

Figure S22. ^{1}H NMR (600 MHz, MeOH-d_4) spectrum of 4. 30

Figure S23. ^{13}C NMR (150 MHz, MeOH-d_4) spectrum of 4. 31

Figure S24. ^{1}H-^{1}H COSY (600 MHz, MeOH-d_4) spectrum of 4. 32

Figure S25. HSQC (600/150 MHz, MeOH-d_4) spectrum of 4. 33

Figure S26. HMBC (600/150 MHz, MeOH-d_4) spectrum of 4. 34

Figure S27. ROESY (600/150 MHz, MeOH-d_4) spectrum of 4. 35

Section S9: HRESIMS spectrum of 4 36

Section S10: Comparison of ^{1}H NMR and ^{1}H-^{1}H COSY spectra between 4r and 4s 37

Figure S29. Comparison of ^{1}H NMR spectra (600 MHz, pyridine-d_5) between 4r and 4s.... 37
Figure S30. The ^{1}H-^{1}H COSY spectrum (600 MHz, pyridine-d_5) of 4r. 38
Figure S31. The ^{1}H-^{1}H COSY spectrum (600 MHz, pyridine-d_5) of 4r. 39
Section S11: 1D and 2D NMR spectra of compound 5 40
Figure S32. ^{1}H NMR (600 MHz, MeOH-d_4) spectrum of 5. 40
Figure S33. ^{13}C NMR (150 MHz, MeOH-d_4) spectrum of 5. 41
Figure S34. ^{1}H-^{1}H COSY (600 MHz, MeOH-d_4) spectrum of 5. 42
Figure S35. HSQC (600/150 MHz, MeOH-d_4) spectrum of 5. 43
Figure S36. HMBC (600/150 MHz, MeOH-d_4) spectrum of 5. 44
Figure S37. ROESY (600/150 MHz, MeOH-d_4) spectrum of 5. 45
Section S12: HRESIMS spectrum of 5 46
Section S13: 1D and 2D NMR spectra of compound 6 47
Figure S39. ^{1}H NMR (600 MHz, MeOH-d_4) spectrum of 6. 47
Figure S40. ^{13}C NMR (150 MHz, MeOH-d_4) spectrum of 6. 48
Figure S41. ^{1}H-^{1}H COSY (600 MHz, MeOH-d_4) spectrum of 6. 49
Figure S42. HSQC (600/150 MHz, MeOH-d_4) spectrum of 6. 50
Figure S43. HMBC (600/150 MHz, MeOH-d_4) spectrum of 6. 51
Figure S44. ROESY (600/150 MHz, MeOH-d_4) spectrum of 6. 52
Section S14: HRESIMS spectrum of 6 53
Section S15: 1D and 2D NMR spectra of compound 7 54
Figure S46. ^{1}H NMR (600 MHz, MeOH-d_4) spectrum of 7. 54
Figure S47. ^{13}C NMR (150 MHz, MeOH-d_4) spectrum of 7. 55
Figure S48. ^{1}H-^{1}H COSY (600 MHz, MeOH-d_4) spectrum of 7. 56
Figure S49. HSQC (600/150 MHz, MeOH-d_4) spectrum of 7. 57
Figure S50. HMBC (600/150 MHz, MeOH-d_4) spectrum of 7. 58
Figure S51. ROESY (600/150 MHz, MeOH-d_4) spectrum of 7. 59
Section S16: HRESIMS spectrum of 7 60
Section S17: 1D and 2D NMR spectra of compound 8 61
Figure S53. ^{1}H NMR (600 MHz, MeOH-d_4) spectrum of 8. 61
Figure S54. ^{13}C NMR (150 MHz, MeOH-d_4) spectrum of 8. 62
Figure S55. ^{1}H-^{1}H COSY (600 MHz, MeOH-d_4) spectrum of 8. 63
Figure S56. HSQC (600/150 MHz, MeOH-d_4) spectrum of 8. 64
Figure S57. HMBC (600/150 MHz, MeOH-d_4) spectrum of 8. 65
Figure S58. ROESY (600 MHz, MeOH-d_4) spectrum of 8..66
Section S18: HRESIMS spectrum of 8 67
Section S19: 1D and 2D NMR spectra of compound 9 68

Figure S60. ^{1}H NMR (600 MHz, $CDCl_3$) spectrum of 9 68
Figure S61. ^{13}C NMR (150 MHz, $CDCl_3$) spectrum of 9 69
Figure S62. ^{1}H-^{1}H COSY (600 MHz, $CDCl_3$) spectrum of 9. 70
Figure S63. HSQC (600/150 MHz, $CDCl_3$) spectrum of 9. 71
Figure S64. HMBC (600/150 MHz, $CDCl_3$) spectrum of 9. 72
Figure S65. ROESY (600/150 MHz, $CDCl_3$) spectrum of 9. 73
Section S20: HRESIMS spectrum of 9 74
Section S21: 1D and 2D NMR spectra of compound 10 75
Figure S67. ^{1}H NMR (600 MHz, MeOH-d_4) spectrum of 10. 75
Figure S68. ^{13}C NMR (150 MHz, MeOH-d_4) spectrum of 10. 76
Figure S69. ^{1}H-^{1}H COSY (600 MHz, MeOH-d_4) spectrum of 10. 77
Figure S70. HSQC (600/150 MHz, MeOH-d_4) spectrum of 10. 78
Figure S71. HMBC (600/150 MHz, MeOH-d_4) spectrum of 10. 79
Figure S72. ROESY (600/150 MHz, MeOH-d_4) spectrum of 10. 80
Section S22: HRESIMS spectrum of 10 81
Section S23: 1D and 2D NMR spectra of compound 11 82
Figure S74. ^{1}H NMR (600 MHz, MeOH-d_4) spectrum of 11. 82
Figure S75. ^{13}C NMR (150 MHz, MeOH-d_4) spectrum of 11. 83
Figure S76. ^{1}H-^{1}H COSY (600 MHz, MeOH-d_4) spectrum of 11. 84
Figure S77. HSQC (600/150 MHz, MeOH-d_4) spectrum of 11. 85
Figure S78. HMBC (600/150 MHz, MeOH-d_4) spectrum of 11. 86
Figure S79. ROESY (600/150 MHz, MeOH-d_4) spectrum of 11. 87
Section S24: HRESIMS spectrum of 11 88
Section S25: 1D and 2D NMR spectra of compound 12 89
Figure S81. ^{1}H NMR (600 MHz, MeOH-d_4) spectrum of 12. 89
Figure S82. ^{13}C NMR (150 MHz, MeOH-d_4) spectrum of 12. 90
Figure S83. ^{1}H-^{1}H COSY (600 MHz, MeOH-d_4) spectrum of 12. 91
Figure S84. HSQC (600/150 MHz, MeOH-d_4) spectrum of 12. 92
Figure S85. HMBC (600/150 MHz, MeOH-d_4) spectrum of 12. 93
Figure S86. ROESY (600/150 MHz, MeOH-d_4) spectrum of 12. 94
Section S26: HRESIMS spectrum of 12 95
Section S27: 1D and 2D NMR spectra of compound 13 96
Figure S88. ^{1}H NMR (600 MHz, MeOH-d_4) spectrum of 13. 96
Figure S89. ^{13}C NMR (150 MHz, MeOH-d_4) spectrum of 13. 97
Figure S90. ^{1}H-^{1}H COSY (600 MHz, MeOH-d_4) spectrum of 13. 98
Figure S91. HSQC (600/150 MHz, MeOH-d_4) spectrum of 13. 99

Figure S92. HMBC (600/150 MHz, MeOH-d_4) spectrum of 13. .... **100**

Figure S93. ROESY (600/150 MHz, MeOH-d_4) spectrum of 13. .... **101**

Section S28: HRESIMS spectrum of 13 .... **102**

Section S29: 1D and 2D NMR spectra of compound 14 .... **103**

Figure S95. ^{1}H NMR (600 MHz, MeOH-d_4) spectrum of 14. .... **103**

Figure S96. ^{13}C NMR (150 MHz, MeOH-d_4) spectrum of 14. .... **104**

Figure S97. ^{1}H-^{1}H COSY (600 MHz, MeOH-d_4) spectrum of 14. .... **105**

Figure S98. HSQC (600/150 MHz, MeOH-d_4) spectrum of 14. .... **106**

Figure S99. HMBC (600/150 MHz, MeOH-d_4) spectrum of 14. .... **107**

Figure S100. ROESY (600/150 MHz, MeOH-d_4) spectrum of 14. .... **108**

Section S30: HRESIMS spectrum of 14 .... **109**

Section S31: 1D and 2D NMR spectra of compound 15 .... **110**

Figure S102. Comparison of the ^{1}H NMR (600 MHz, MeOH-d_4) spectra of 15 and 28. .... **110**

Section S32: HRESIMS spectrum of 15 .... **111**

Figure S103. HRESIMS spectrum of 15. .... **111**

Section S33: 1D and 2D NMR spectra of compound 16 .... **112**

Figure S104. ^{1}H NMR (600 MHz, $CDCl_3$) spectrum of 16. .... **112**

Figure S105. ^{13}C NMR (150 MHz, $CDCl_3$) spectrum of 16. .... **113**

Figure S106. ^{1}H-^{1}H COSY (600 MHz, $CDCl_3$) spectrum of 16. .... **114**

Figure S107. HSQC (600/150 MHz, $CDCl_3$) spectrum of 16. .... **115**

Figure S108. HMBC (600/150 MHz, $CDCl_3$) spectrum of 16. .... **116**

Figure S109. ROESY (600/150 MHz, $CDCl_3$) spectrum of 16. .... **117**

Section S34: HRESIMS spectrum of 16 .... **118**

Section S1: 1D and 2D NMR spectra of compound 1

Figure S1. **^{1}H NMR (600 MHz, MeOH-d_4) spectrum of 1.**

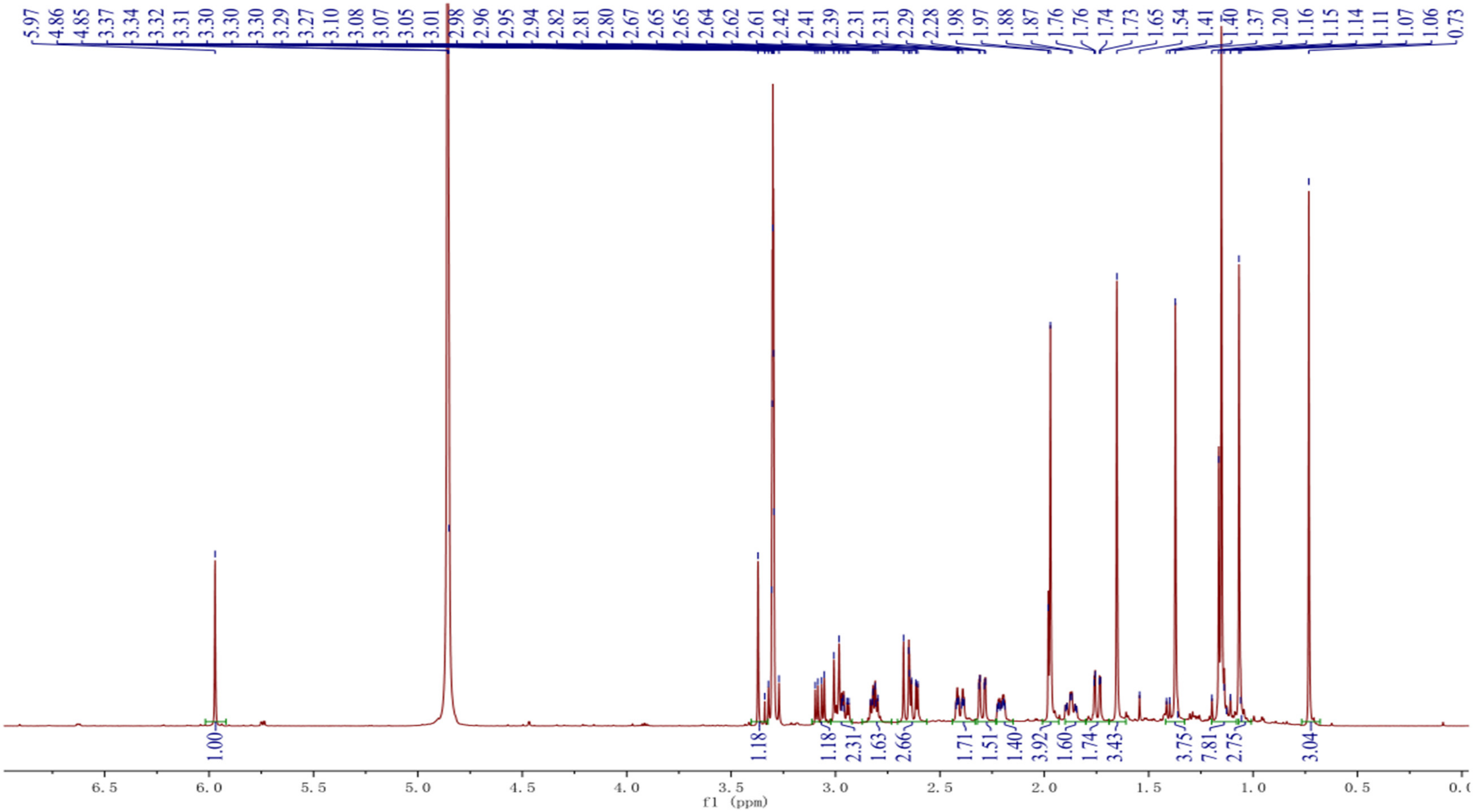

Figure S2. ^{13}C **NMR (150 MHz, MeOH-*d_4*) spectrum of 1.**

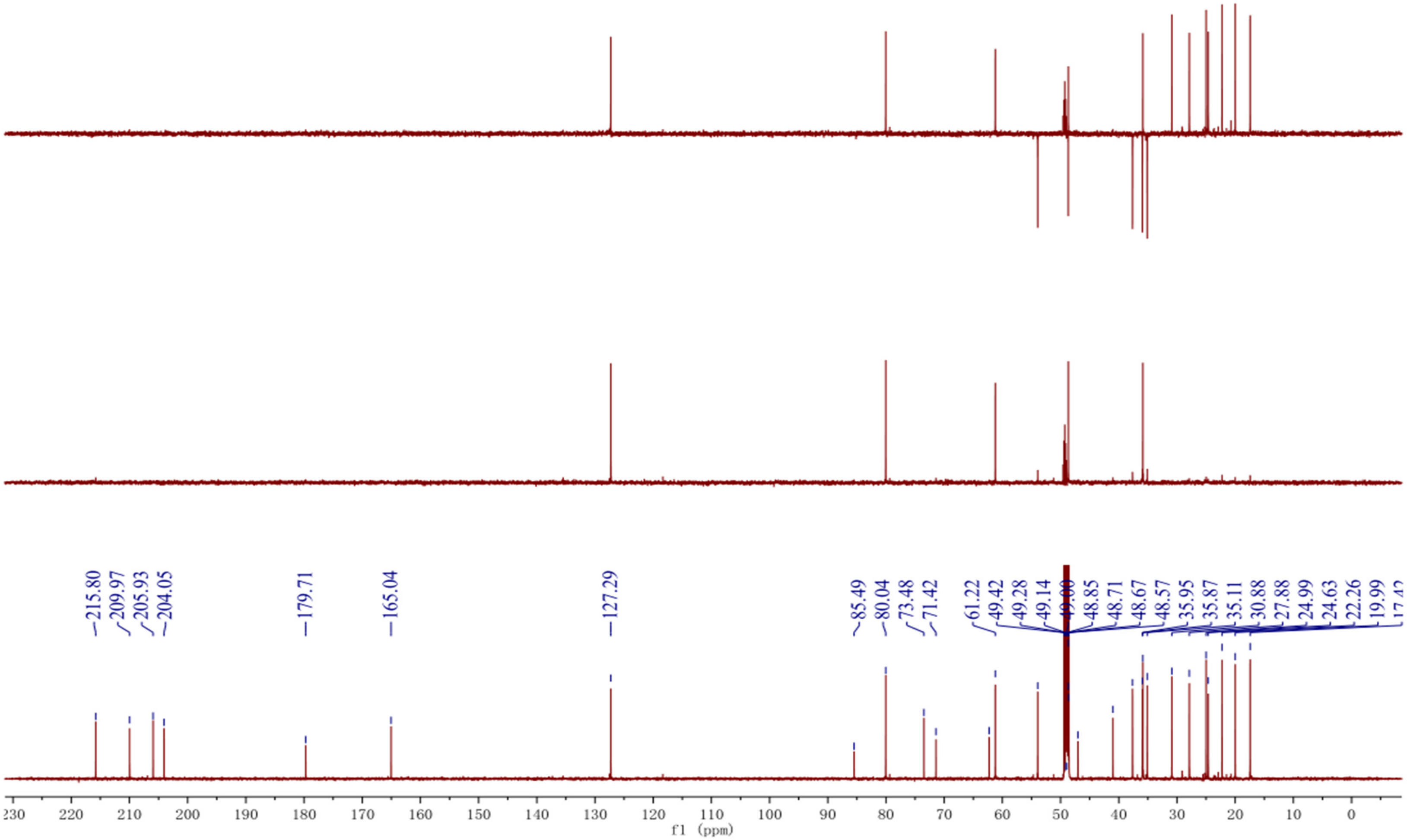

Figure S3. ^{1}**H-**^{1}**H COSY (600 MHz, MeOH-*d*$_4$) spectrum of 1.**

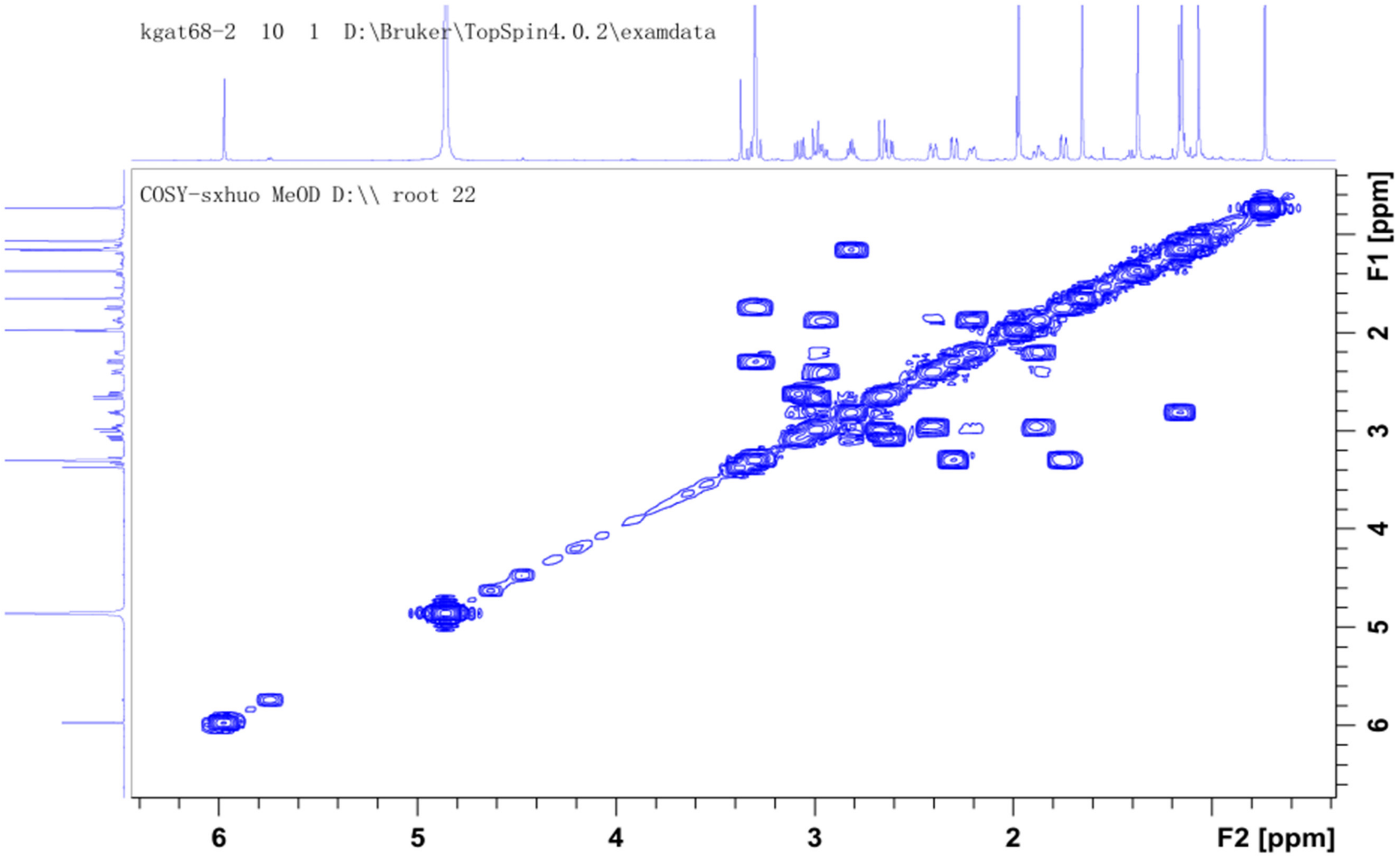

Figure S4. HSQC (600/150 MHz, MeOH-d_4) spectrum of 1.

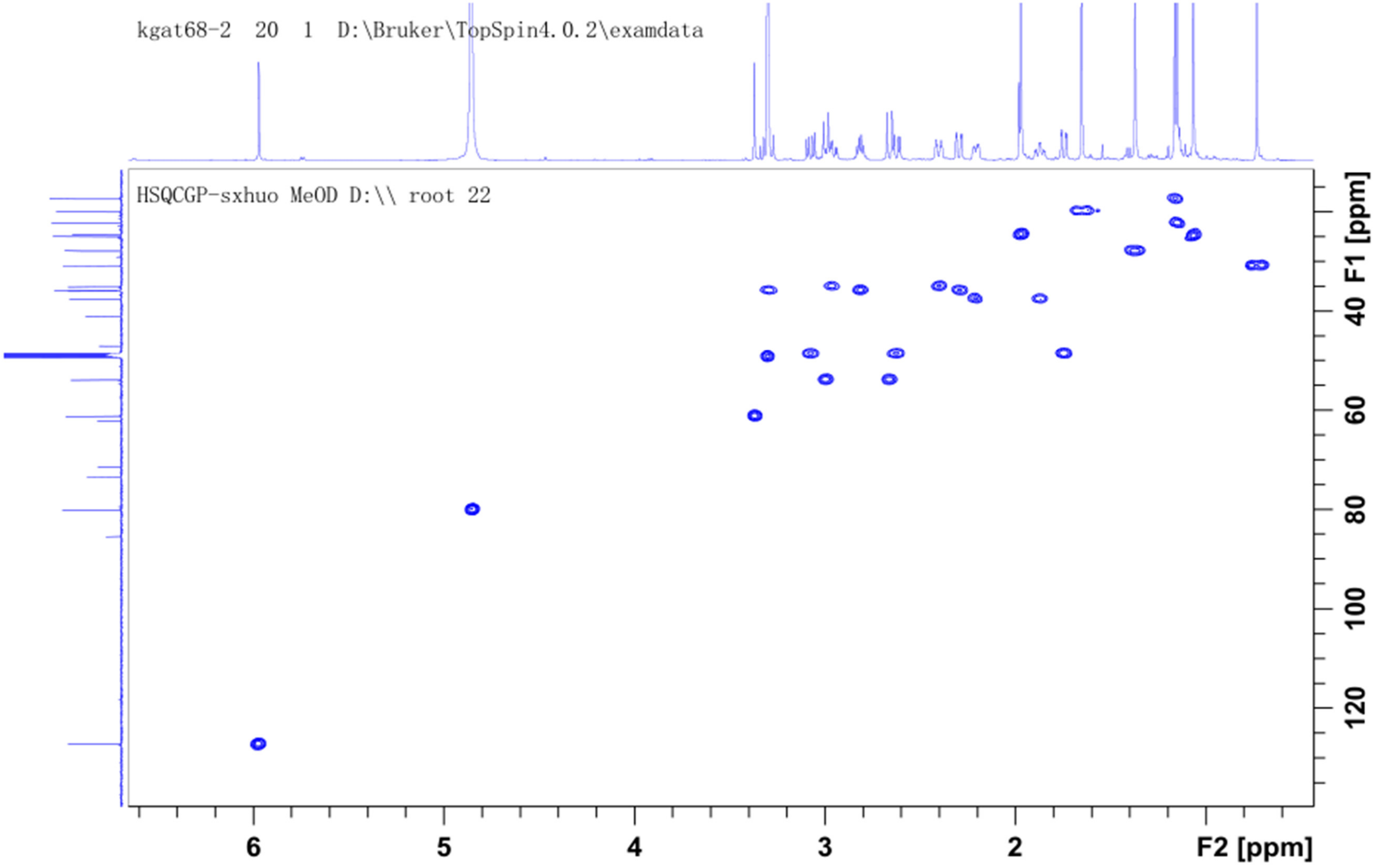

Figure S5. HMBC (600/150 MHz, MeOH-d_4) spectrum of 1.

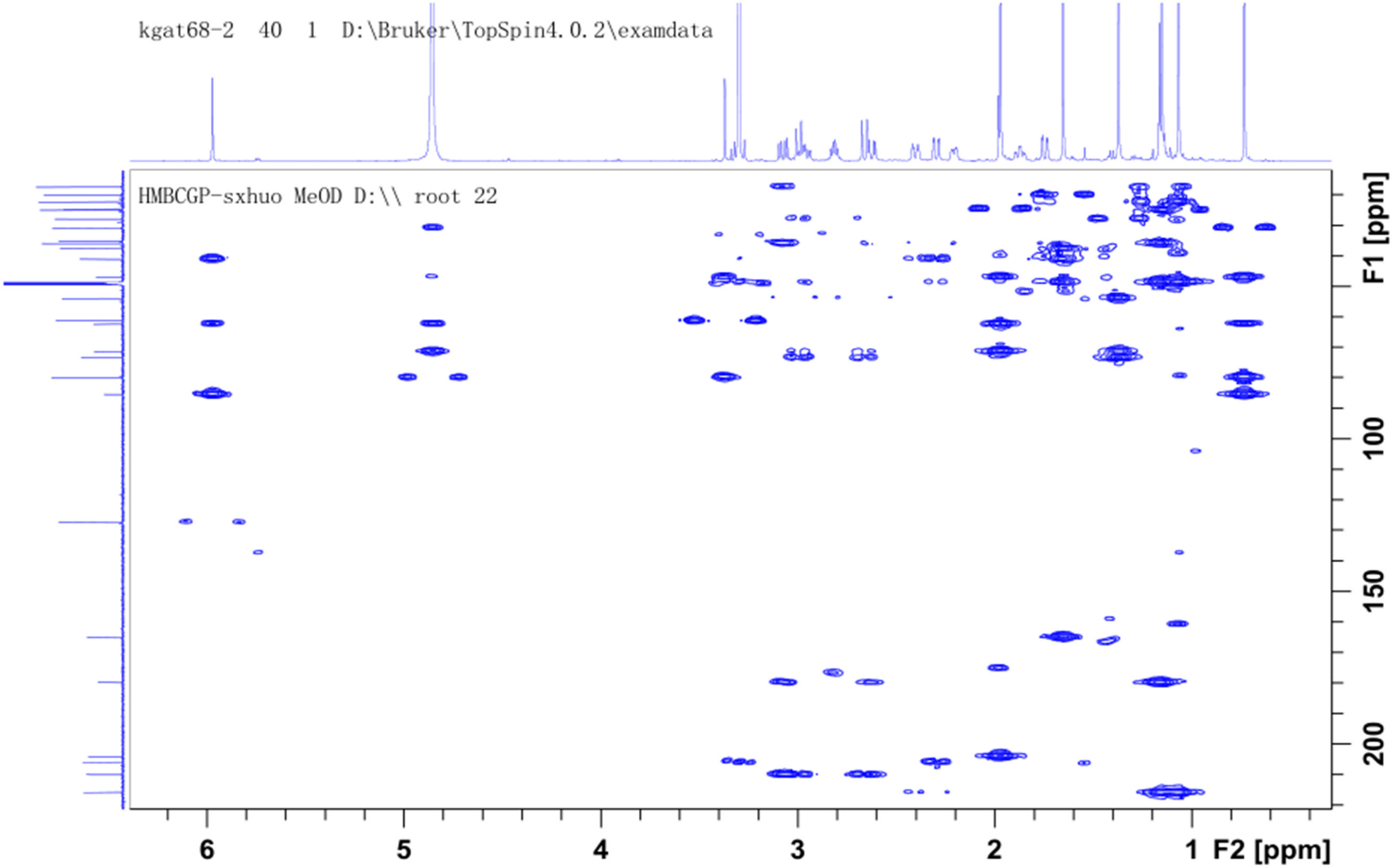

Figure S6. ROESY (600/150 MHz, MeOH-d_4) spectrum of 1.

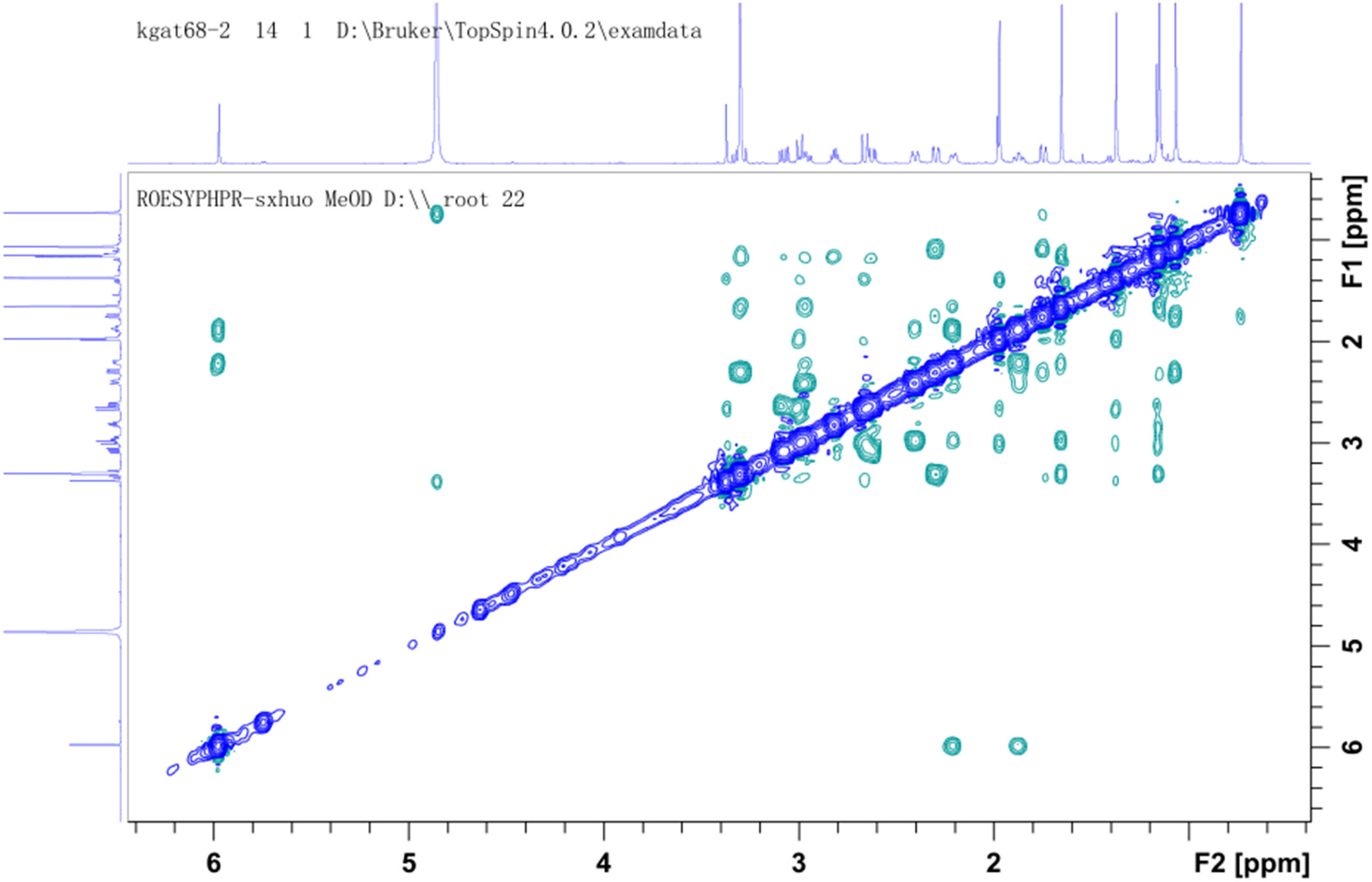

Section S2: HRESIMS spectrum of 1

Figure S7. HRESIMS spectrum of 1.

Qualitative Analysis Report

**Data Filename**	kgat68-2.d	**Sample Name**	kgat68-2
**Sample Type**	Sample	**Position**	P1-A2
**Instrument Name**	Instrument 1	**User Name**	
**Acq Method**	s.m	**Acquired Time**	2/22/2022 11:14:49 AM
**IRM Calibration Status**	Success	**DA Method**	PCDL.m
**Comment**			

Sample Group **Info.**
Acquisition SW 6200 series TOF/6500 series
Version Q-TOF B.05.01 (B5125.2)

User Spectra

Fragmentor Voltage	Collision Energy	Ionization Mode
135	0	ESI

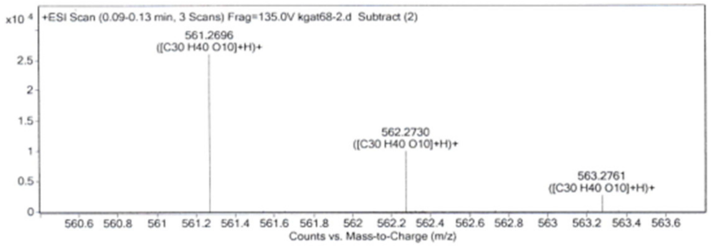

Peak List

m/z	z	Abund	Formula	Ion
63.998		17268.65		
79.0215		1125543.25		
80.0244		28274.9		
81.0173	1	49077.89		
101.0035		10379.81		
137.0021	1	11767.81		
157.0351	1	34260.16		
525.2488	1	12195.33		
543.2591	1	13386.97		
561.2696	1	26014.12	C30 H40 O10	(M+H)+

Formula Calculator Element Limits

Element	Min	Max
C	3	200
H	0	400
O	0	50

Formula Calculator Results

Formula	CalculatedMass	CalculatedMz	Mz	Diff. (mDa)	Diff. (ppm)	DBE
C30 H40 O10	560.2622	561.2694	561.2696	-0.20	-0.36	11.0000

--- End Of Report ---

Agilent Technologies Page 1 of 1 Printed at: 11:18 AM on: 2/22/2022

Section S3: X-ray crystallographic data of 1

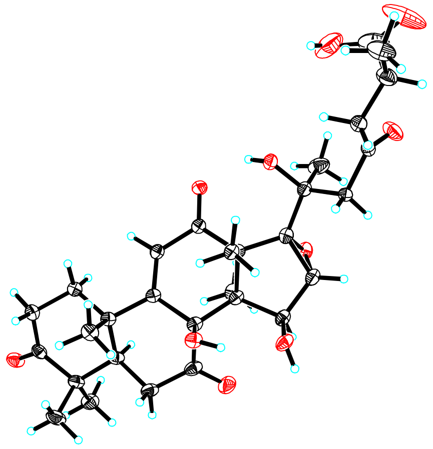

View of a molecule of 1 with the atom-labelling scheme.

Displacement ellipsoids are drawn at the 30% probability level.

Table S1. Crystal data and structure refinement for 1.

Identification code	global
Empirical formula	$C_{31} H_{46} O_{12}$
Formula weight	610.68
Temperature	100(2) K
Wavelength	1.54178 Å
Crystal system	Monoclinic
Space group	P 1 21 1
Unit cell dimensions	a = 13.9761(7) Å, α= 90°; b = 6.9089(3) Å, β= 90.948(2)°; c = 15.8732(7) Å, γ = 90°
Volume	1532.50(12) Å3
Z	2
Density (calculated)	1.323 Mg/m^3
Absorption coefficient	0.844 mm^{-1}
F(000)	656
Crystal size	1.050 x 0.140 x 0.120 mm3
Theta range for data collection	2.78 to 72.57°
Index ranges	-17<=h<=17, -8<=k<=8, -19<=l<=19
Reflections collected	26208
Independent reflections	5973 [R(int) = 0.0593]
Completeness to theta = 72.57°	99.6 %
Absorption correction	Semi-empirical from equivalents
Max. and min. transmission	0.91 and 0.62
Refinement method	Full-matrix least-squares on F2
Data / restraints / parameters	5973 / 352 / 506
Goodness-of-fit on F2	1.042
Final R indices [I>2sigma(I)]	R1 = 0.0504, wR2 = 0.1381
R indices (all data)	R1 = 0.0543, wR2 = 0.1436
Absolute structure parameter	0.07(9)
Largest diff. peak and hole	0.372 and -0.254 e.Å-3

Section S4: 1D and 2D NMR spectra of compound 2

Figure S8. ^{1}**H NMR (600 MHz, MeOH-d_4) spectrum of 2.**

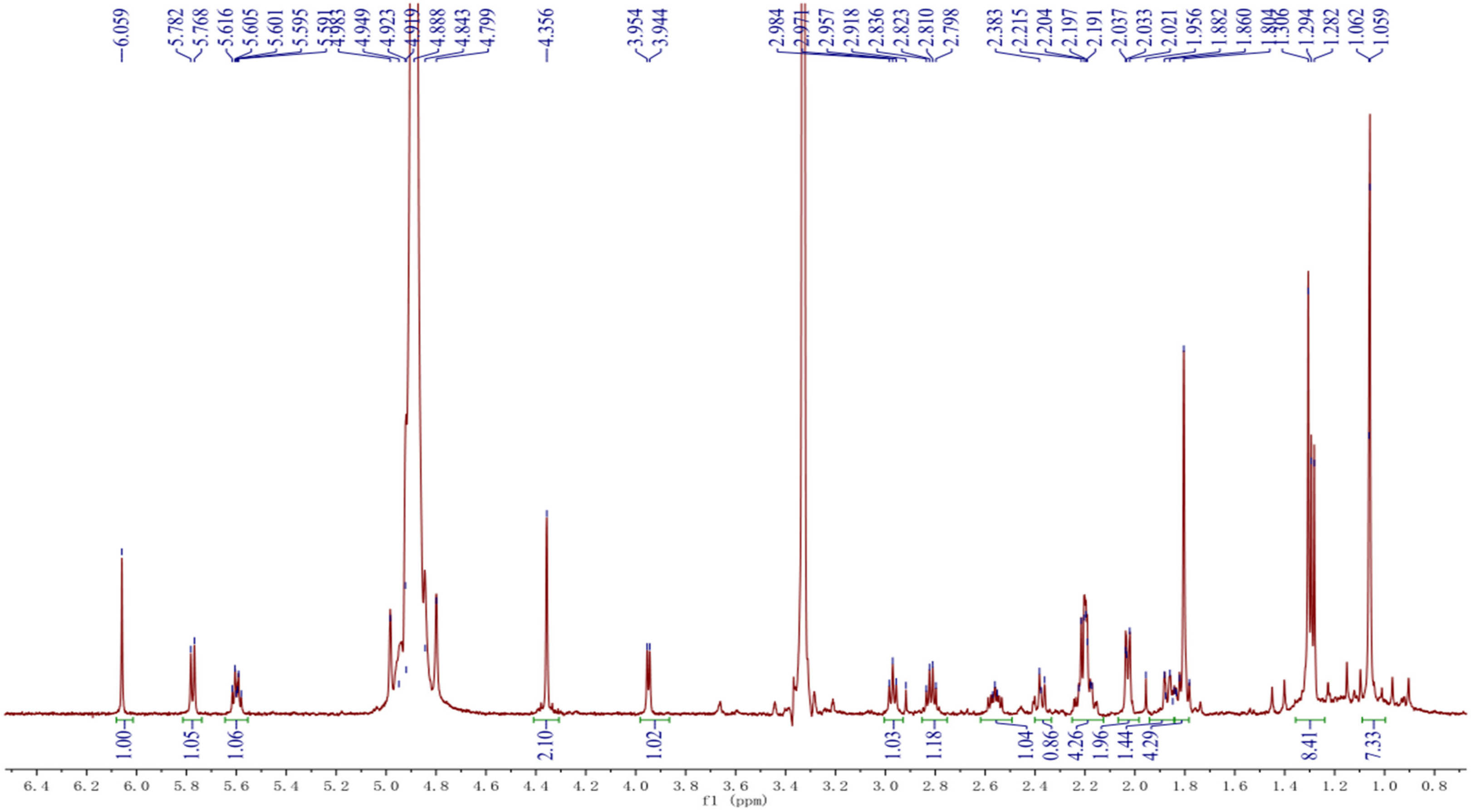

Figure S9. ^{13}C NMR (150 MHz, MeOH-d_4) spectrum of 2.

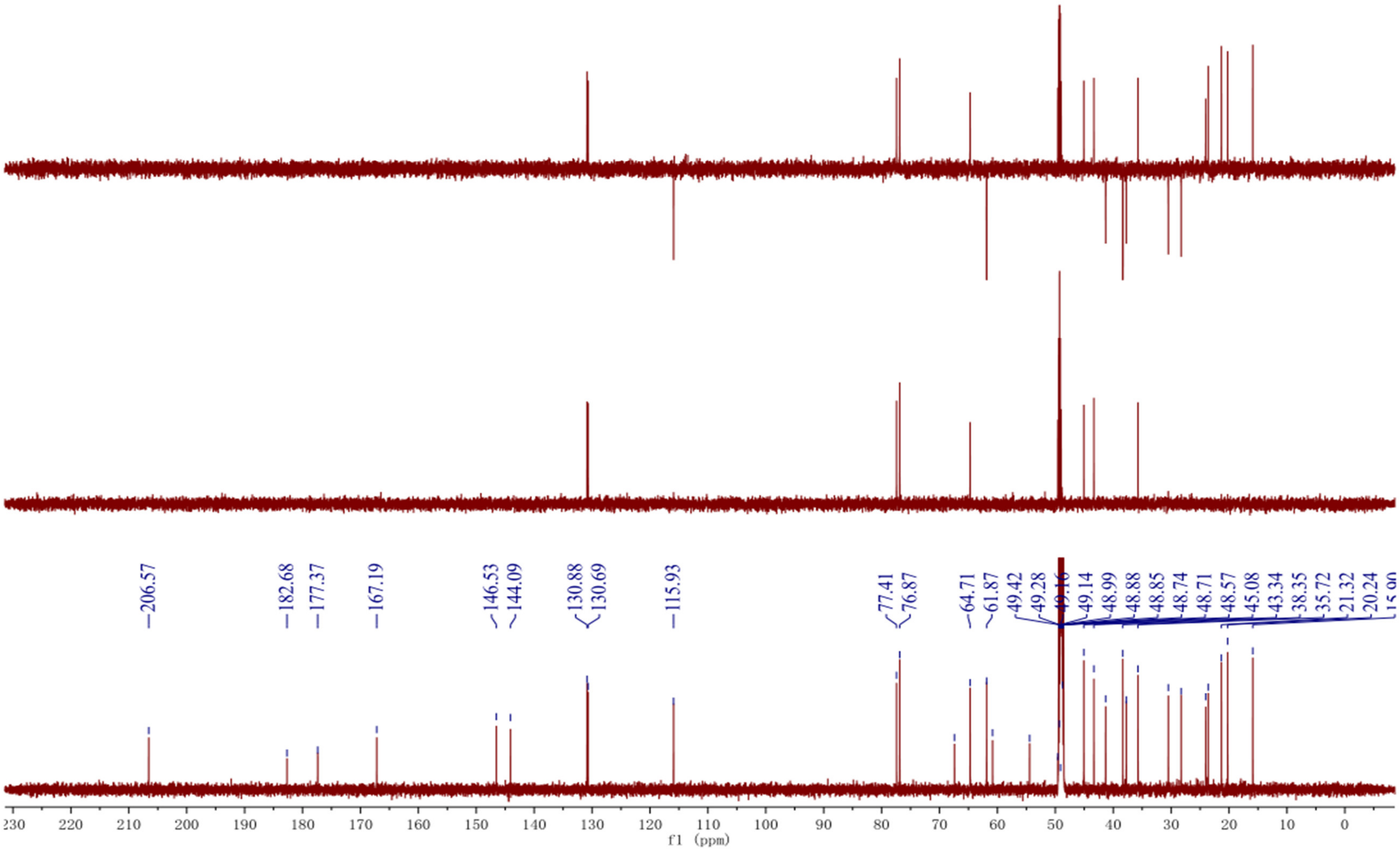

Figure S10. ^{1}H-^{1}H COSY (600 MHz, MeOH-d_4) spectrum of 2.

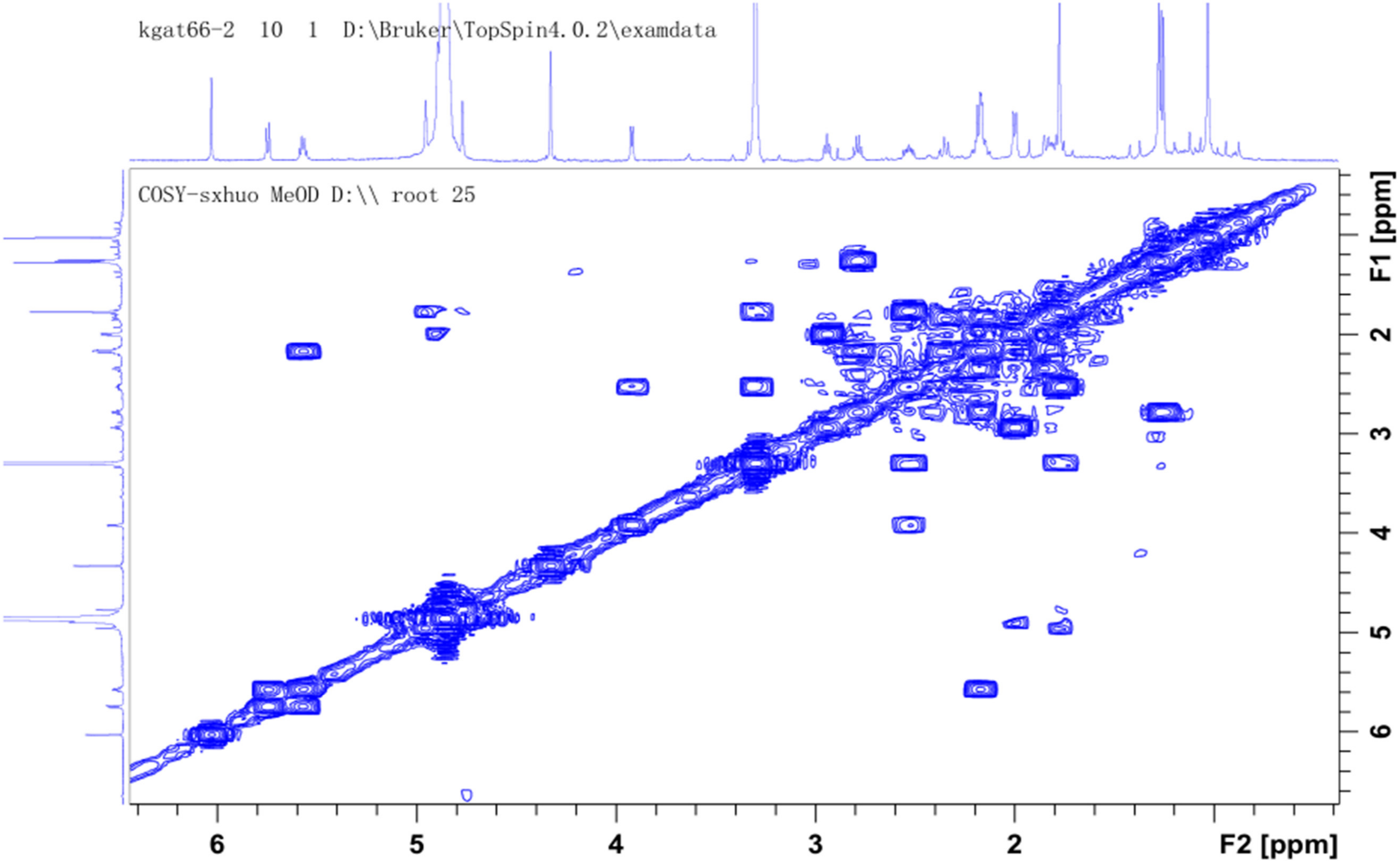

Figure S11. HSQC (600/150 MHz, MeOH-d_4) spectrum of 2.

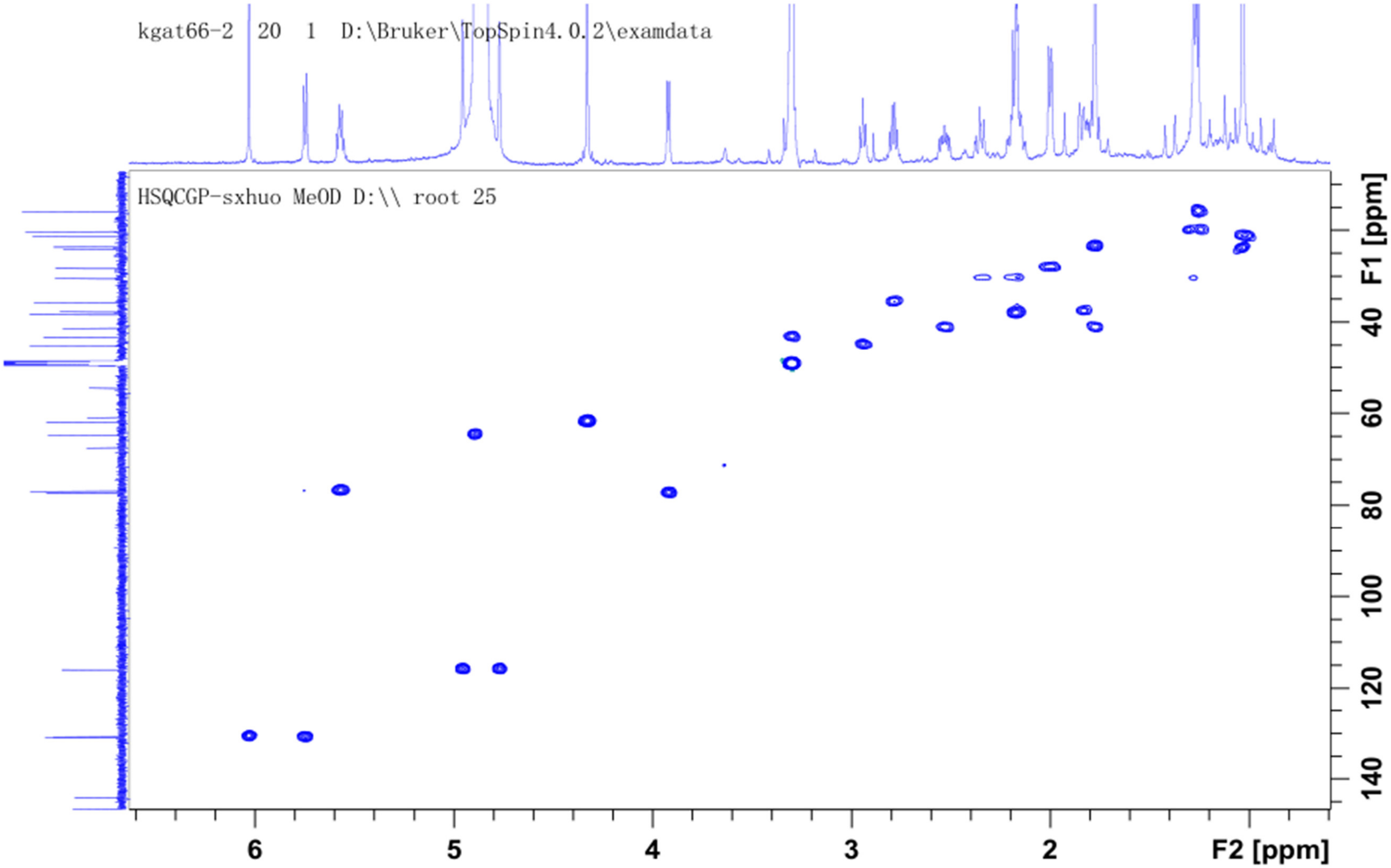

Figure S12. HMBC (600/150 MHz, MeOH-d_4) spectrum of 2.

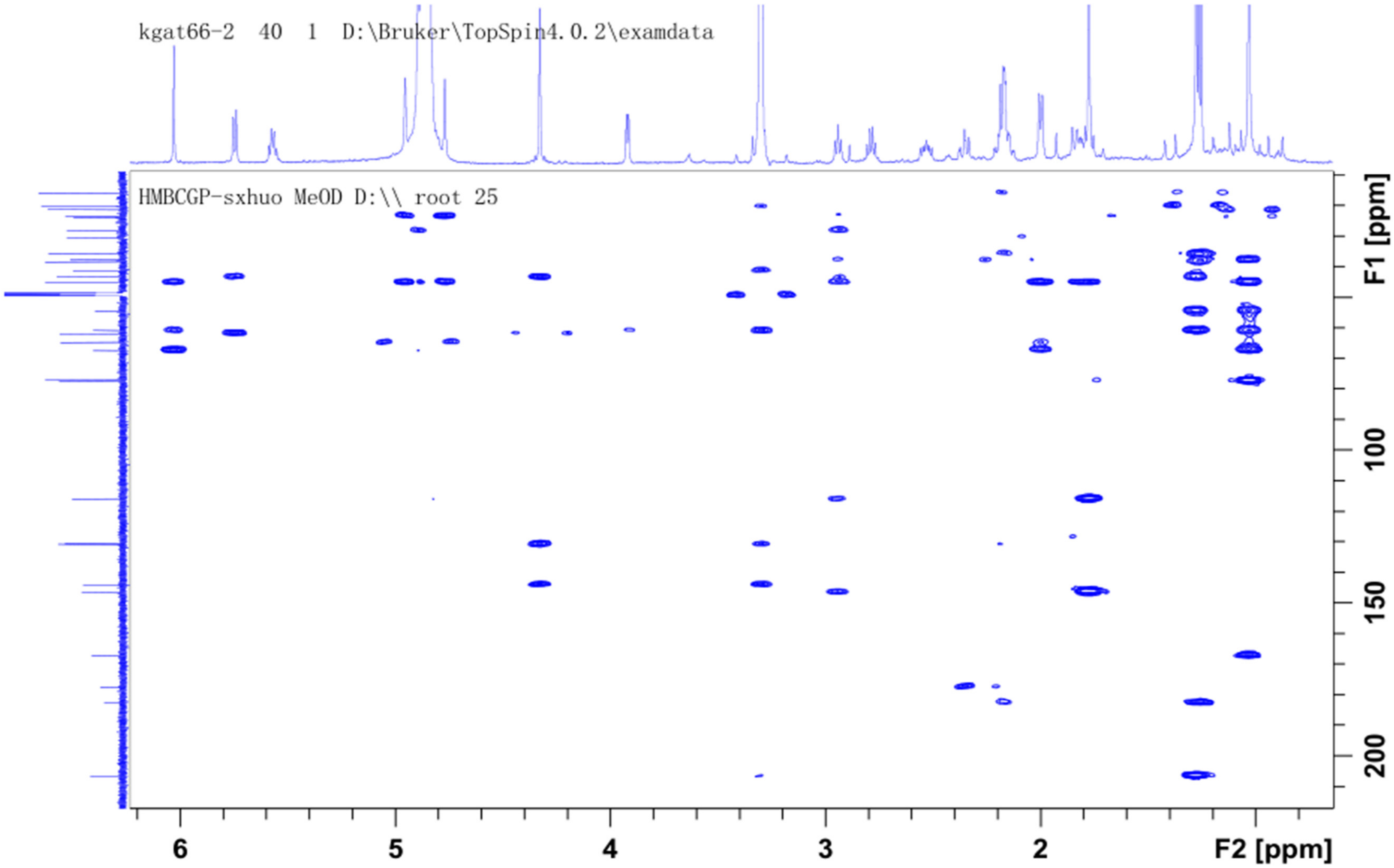

Figure S13. ROESY (600/150 MHz, MeOH-d_4) spectrum of 2.

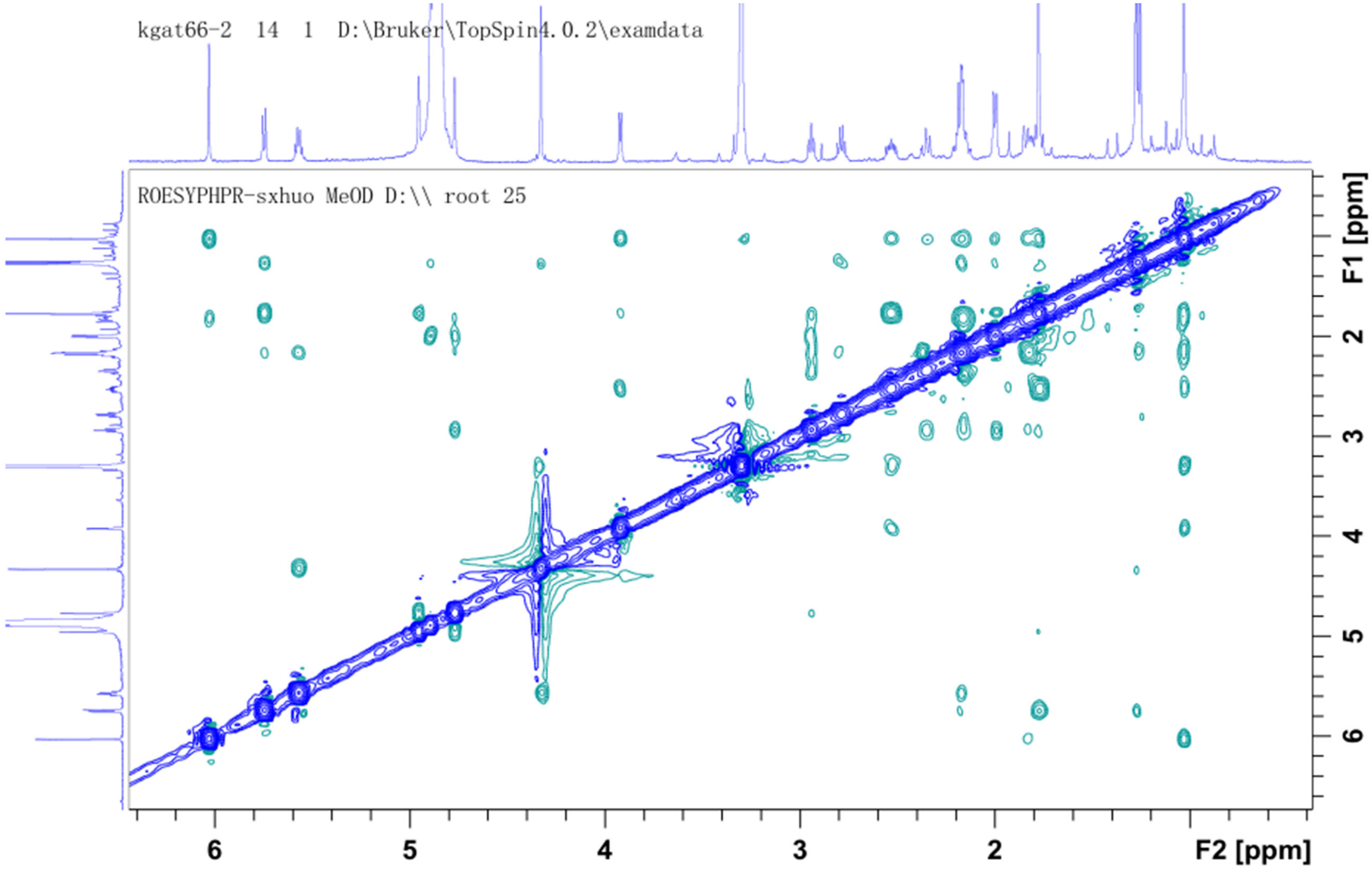

Section S5: HRESIMS spectrum of 2

Figure S14. HRESIMS spectrum of 2.

Qualitative Analysis Report

**Data Filename**	kgat66-2.d	**Sample Name**	kgat66-2
**Sample Type**	Sample	**Position**	P1-A4
**Instrument Name**	Instrument 1	**User Name**	
**Acq Method**	s-.m	**Acquired Time**	7/9/2021 2:54:52 PM
**IRM Calibration Status**	Success	**DA Method**	Default.m
**Comment**			

Sample Group **Info.**

**Acquisition SW**	6200 series TOF/6500 series
**Version**	Q-TOF B.05.01 (B5125.2)

User Spectra

Fragmentor Voltage	Collision Energy	Ionization Mode
180	0	ESI

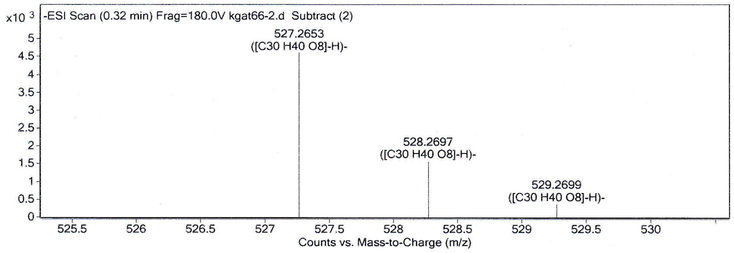

Peak List

m/z	z	Abund	Formula	Ion
324.9885		348.81		
447.5934		347.95		
448.9755		365.66		
500.3118		352.77		
527.2653	1	4608.72	C30 H40 O8	(M-H)-
528.2697	1	1573.13	C30 H40 O8	(M-H)-
529.2699	1	360.96	C30 H40 O8	(M-H)-
573.2709		593.19		
595.2445		440.07		
948.0079		444.48		
1071.9621		314.56		
1306.9568		366.34		

Formula Calculator Element Limits

Element	Min	Max
C	3	60
H	0	120
O	0	30

Formula Calculator Results

Formula	CalculatedMass	CalculatedMz	Mz	Diff. (mDa)	Diff. (ppm)	DBE
C30 H40 O8	528.2723	527.2650	527.2653	-0.30	-0.57	11.0000

--- End Of Report ---

Agilent Technologies Page 1 of 1 Printed at: 3:17 PM on: 7/9/2021

Section S6: 1D and 2D NMR spectra of compound 3

Figure S15. **^{1}H NMR (600 MHz, MeOH-d_4) spectrum of 3.**

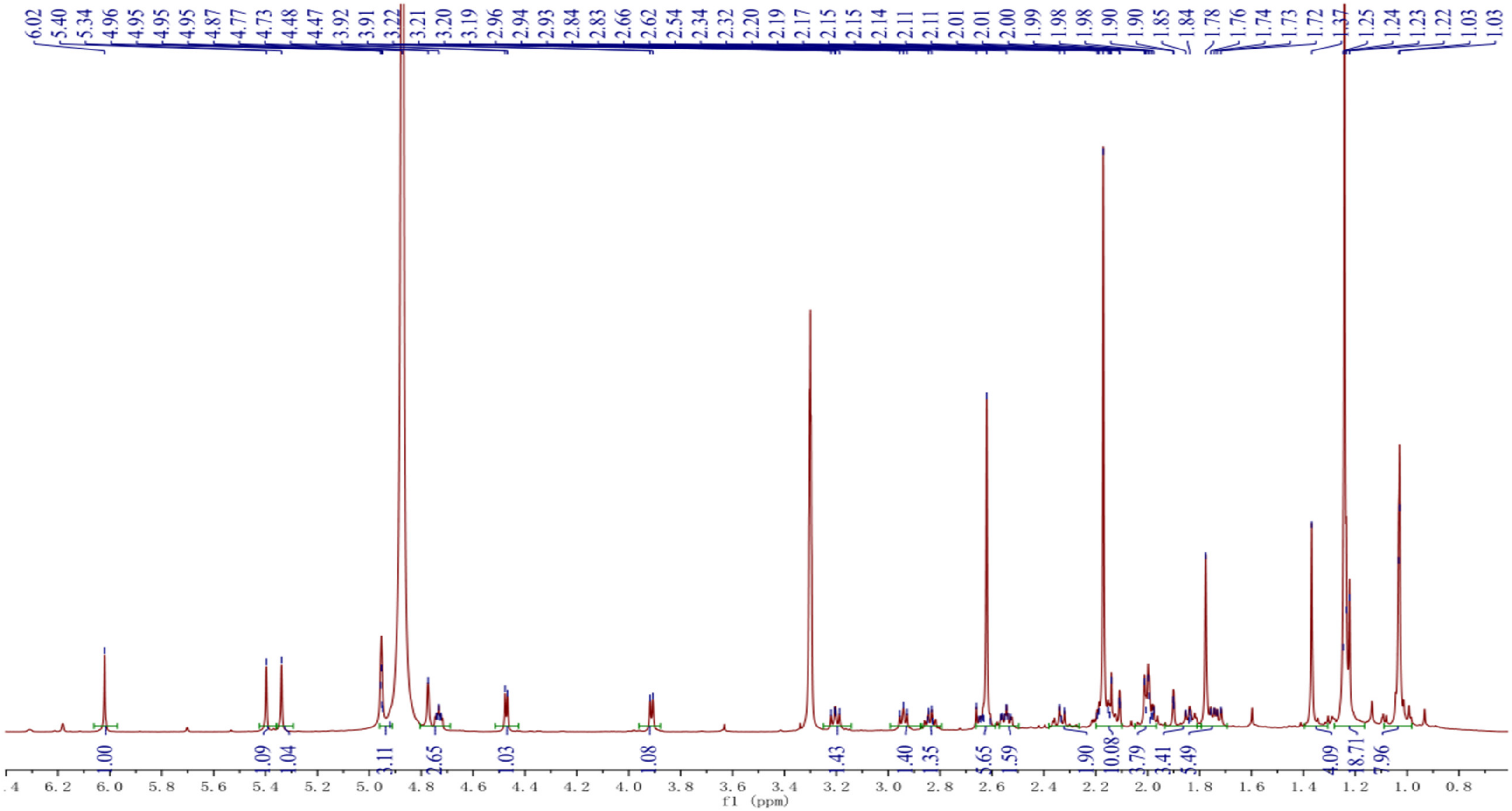

Figure S16. ^{13}**C NMR (150 MHz, MeOH-*d*$_4$) spectrum of 3.**

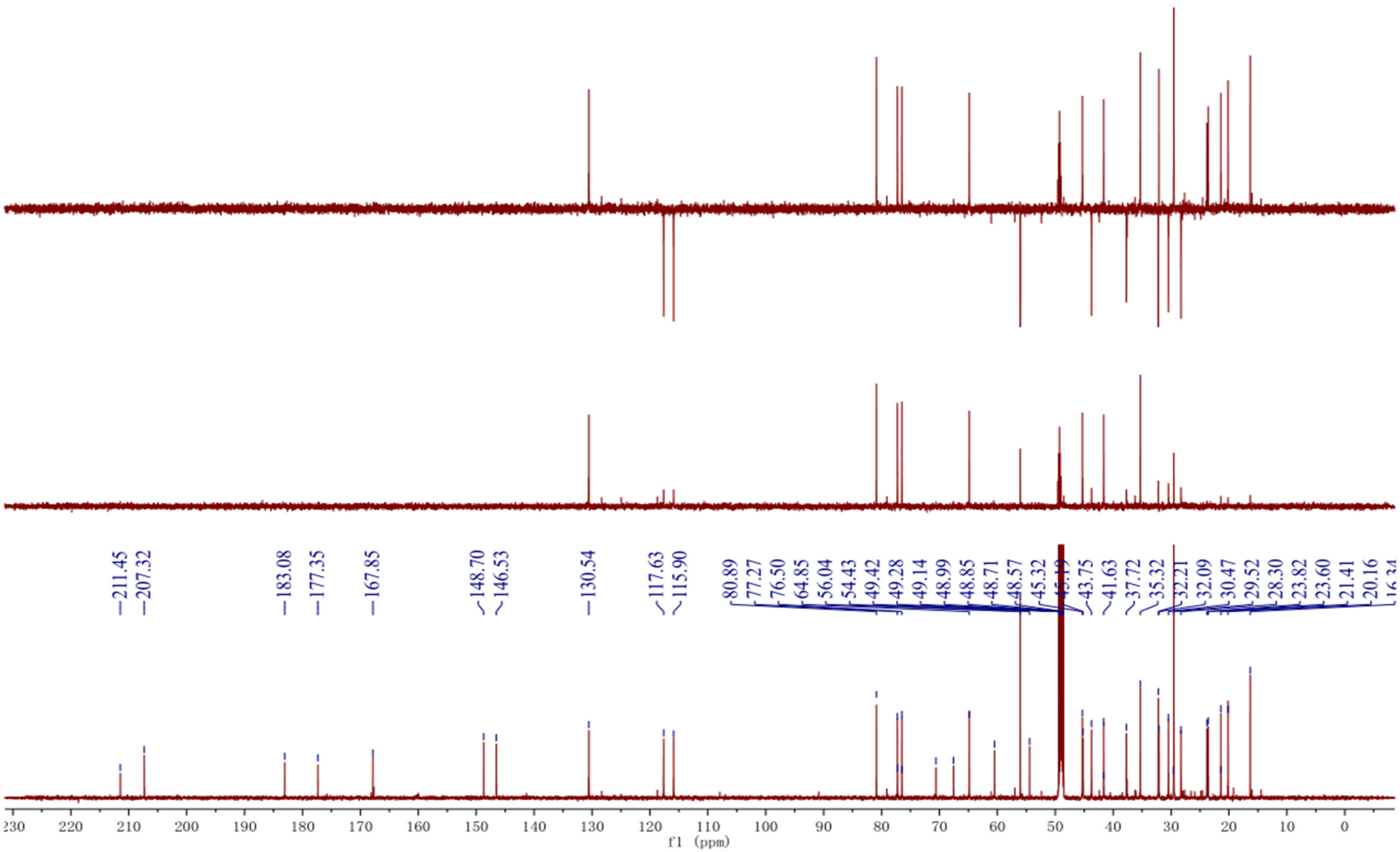

Figure S17. **^{1}H-^{1}H COSY (600 MHz, MeOH-d_4) spectrum of 3.**

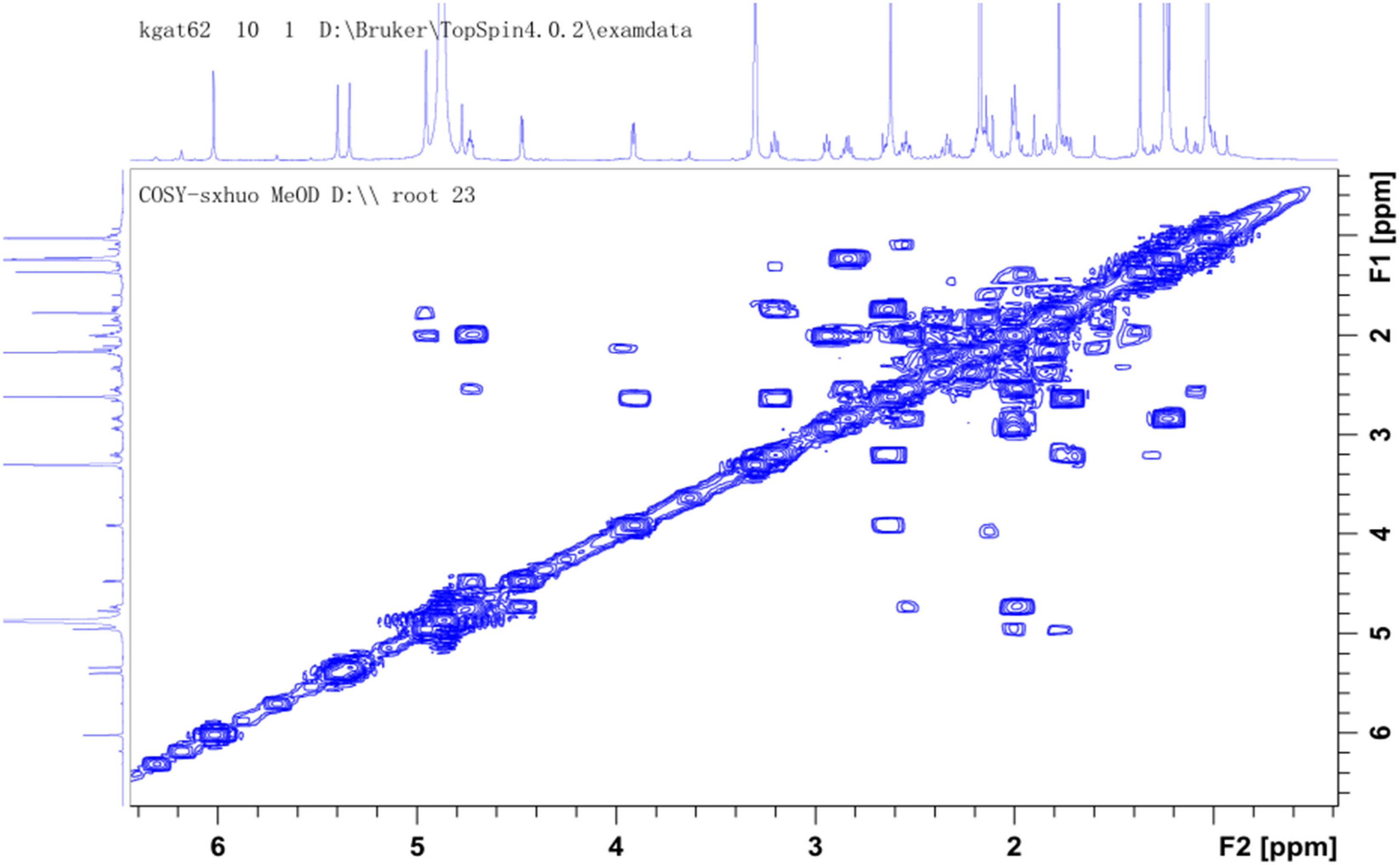

Figure S18. HSQC (600/150 MHz, MeOH-d_4) spectrum of 3.

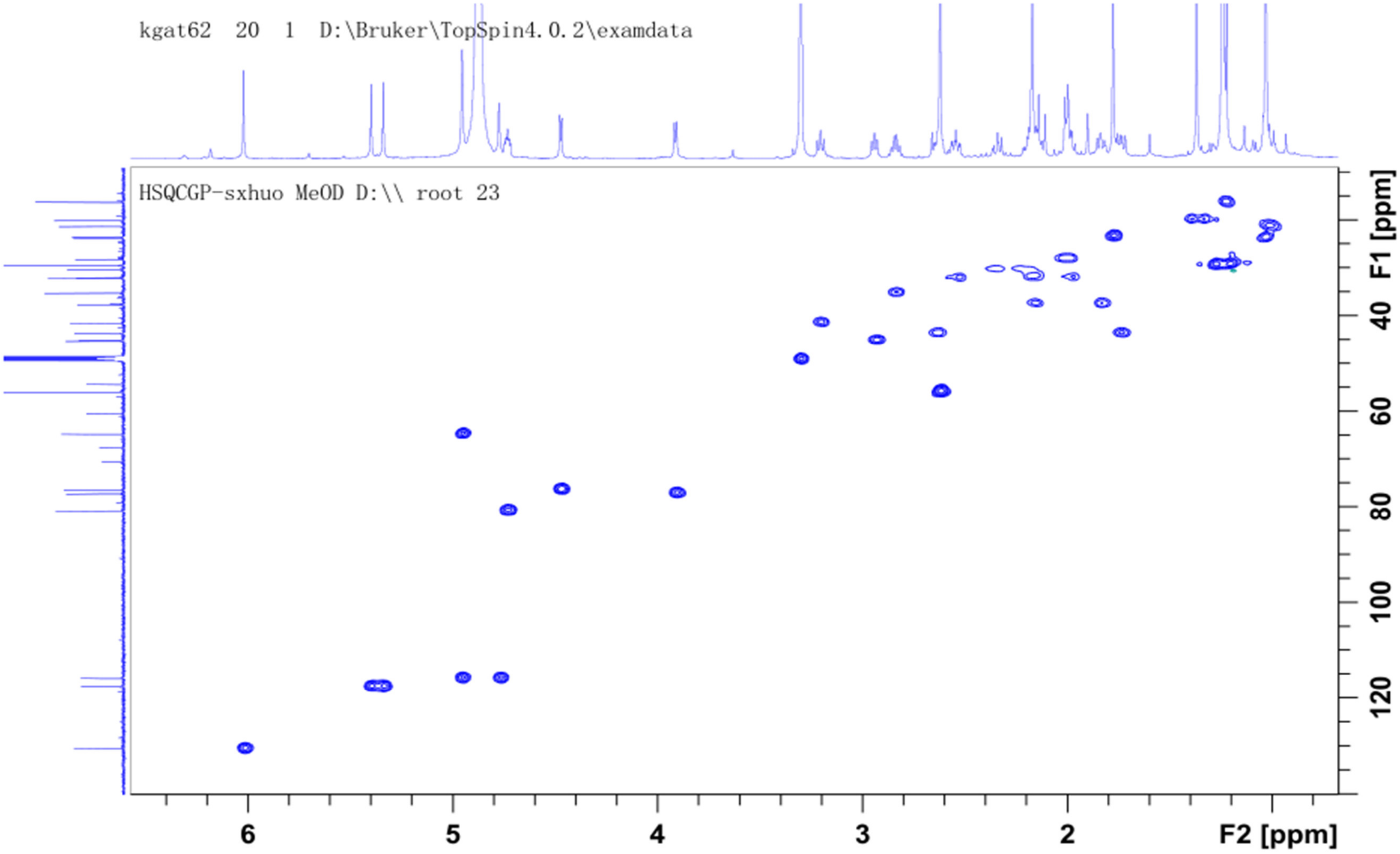

Figure S19. HMBC (600/150 MHz, MeOH-d_4) spectrum of 3.

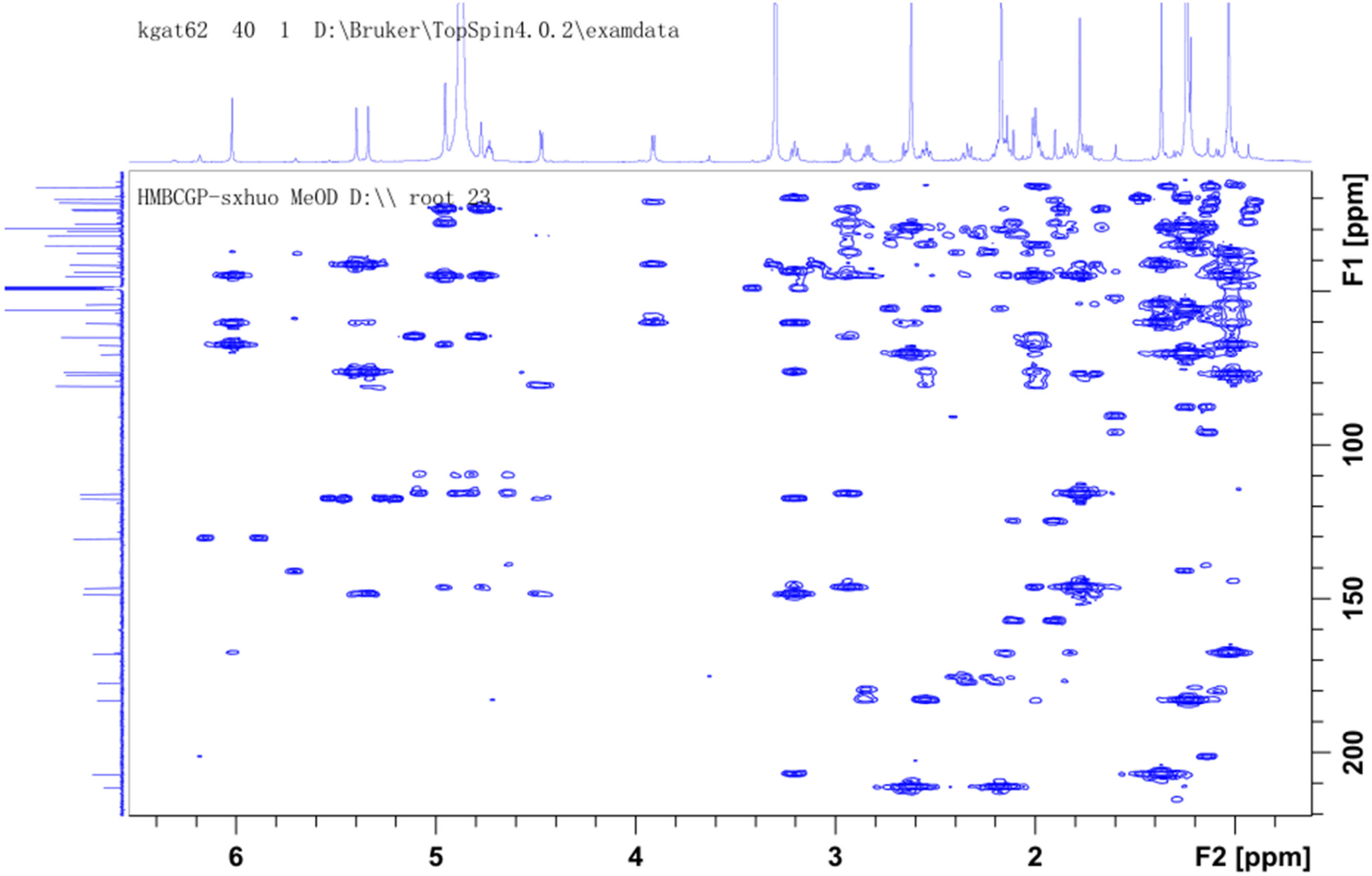

Figure S20. ROESY (600/150 MHz, MeOH-d_4) spectrum of 3.

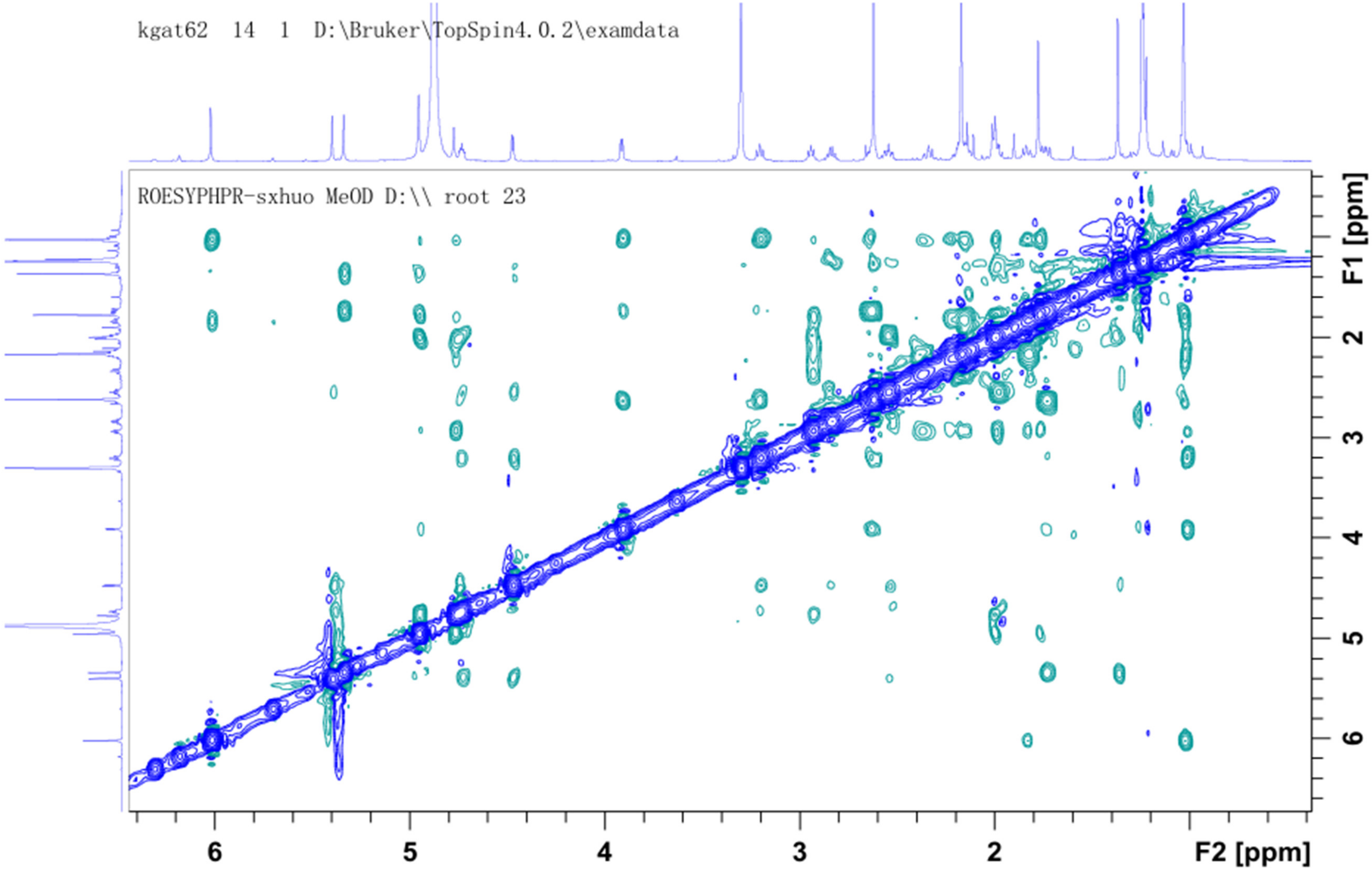

Section S7: HRESIMS spectrum of 3

Figure S21. HRESIMS spectrum of 3.

Qualitative Analysis Report

**Data Filename**	kgat62.d	**Sample Name**	kgat62
**Sample Type**	Sample	**Position**	P1-A4
**Instrument Name**	Instrument 1	**User Name**	
**Acq Method**	s-.m	**Acquired Time**	5/19/2021 10:40:04 AM
**IRM Calibration Status**	Success	**DA Method**	Default.m
**Comment**			

Sample Group **Info.**
Acquisition SW 6200 series TOF/6500 series
Version Q-TOF B.05.01 (B5125.2)

User Spectra

Fragmentor Voltage	Collision Energy	Ionization Mode
135	0	ESI

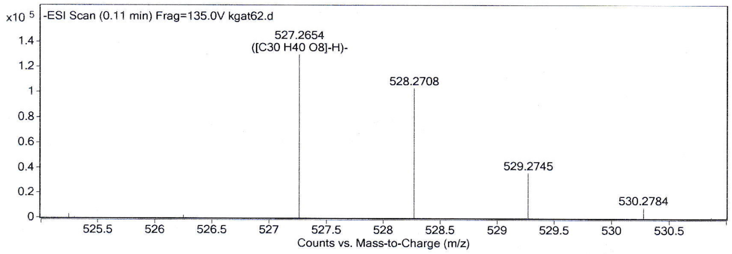

Peak List

m/z	z	Abund	Formula	Ion
112.9856		326571.84		
527.2654		129859.75	C30 H40 O8	(M-H)-
528.2708	1	102946.99		
544.2997		99789.67		
545.306	1	271520.28		
573.2709	1	99540.22		
590.3058	1	644088.69		
591.309	1	201983.73		
1033.9885	1	247649.55		
1055.5377	1	109539.64		
1072.572	1	365939.16		
1073.5753	1	247403.97		

Formula Calculator Element Limits

Element	Min	Max
C	3	60
H	0	120
O	0	30

Formula Calculator Results

Formula	CalculatedMass	CalculatedMz	Mz	Diff. (mDa)	Diff. (ppm)	DBE
C30 H40 O8	528.2723	527.2650	527.2654	-0.40	-0.76	11.0000

--- End Of Report ---

Section S8: 1D and 2D NMR spectra of compound 4

Figure S22. ^{1}**H NMR (600 MHz, MeOH-d_4) spectrum of 4.**

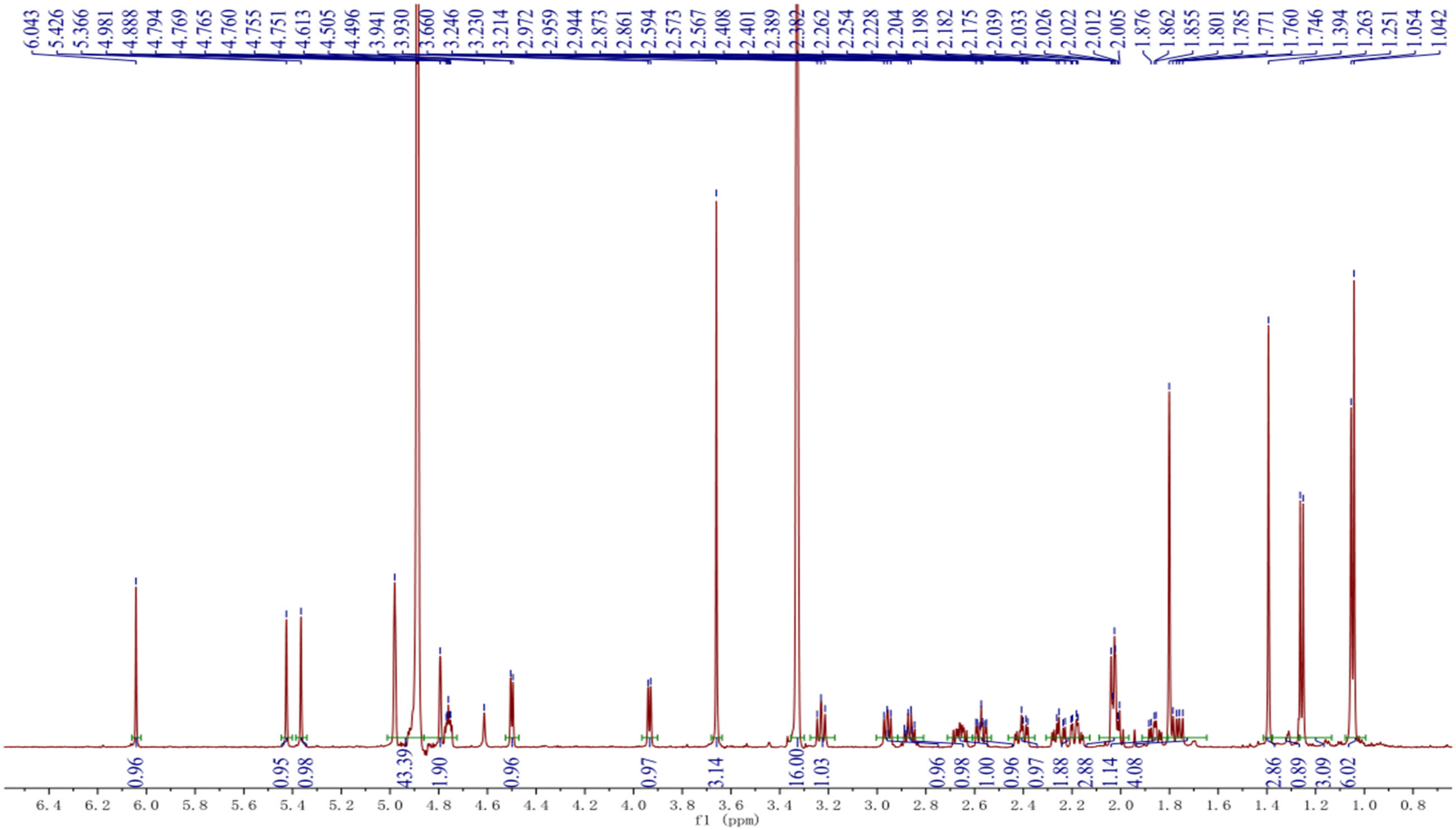

Figure S23. ^{13}C NMR (150 MHz, MeOH-d_4) spectrum of **4**.

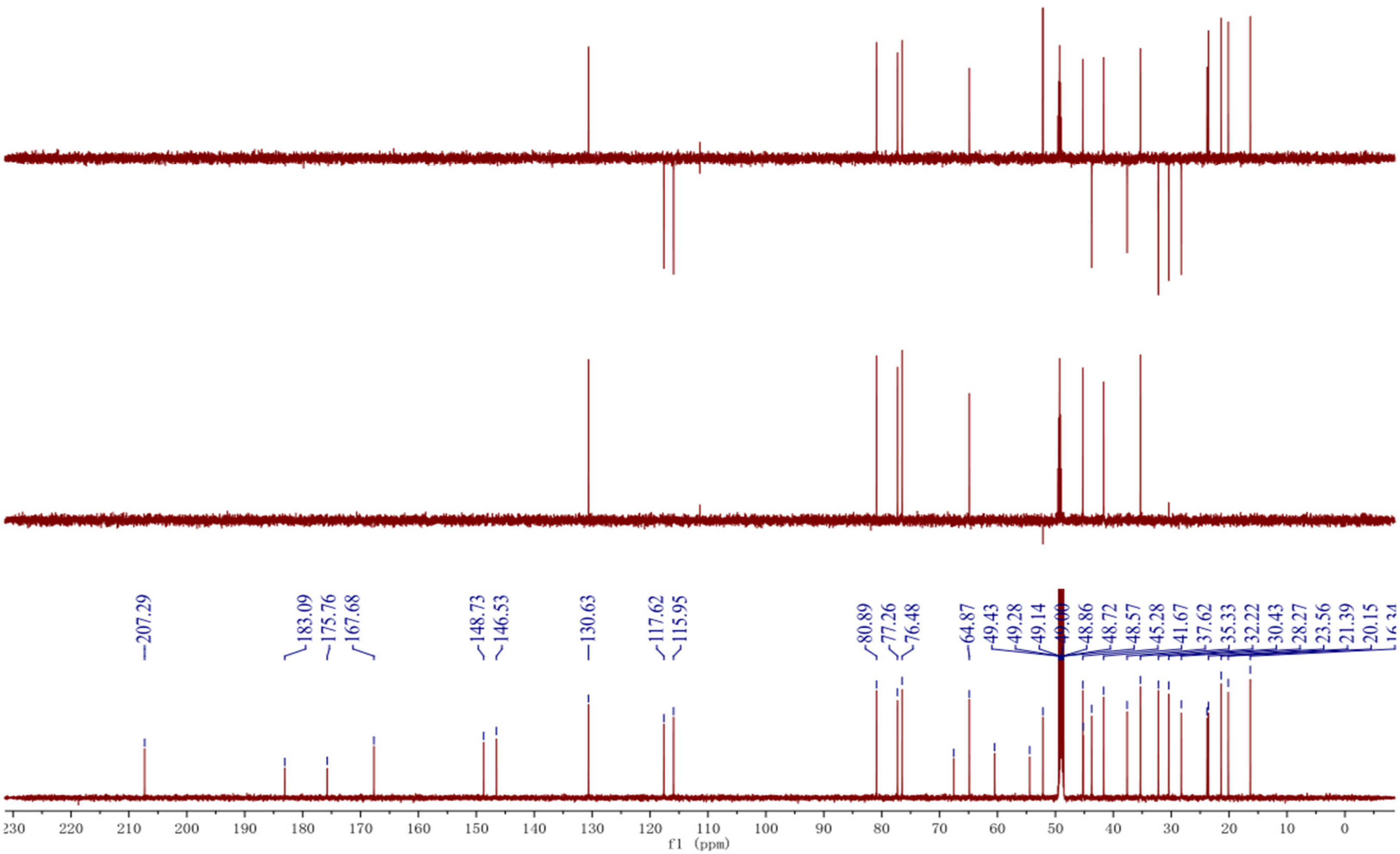

Figure S24. **^1H-^1H COSY (600 MHz, MeOH-d_4) spectrum of 4.**

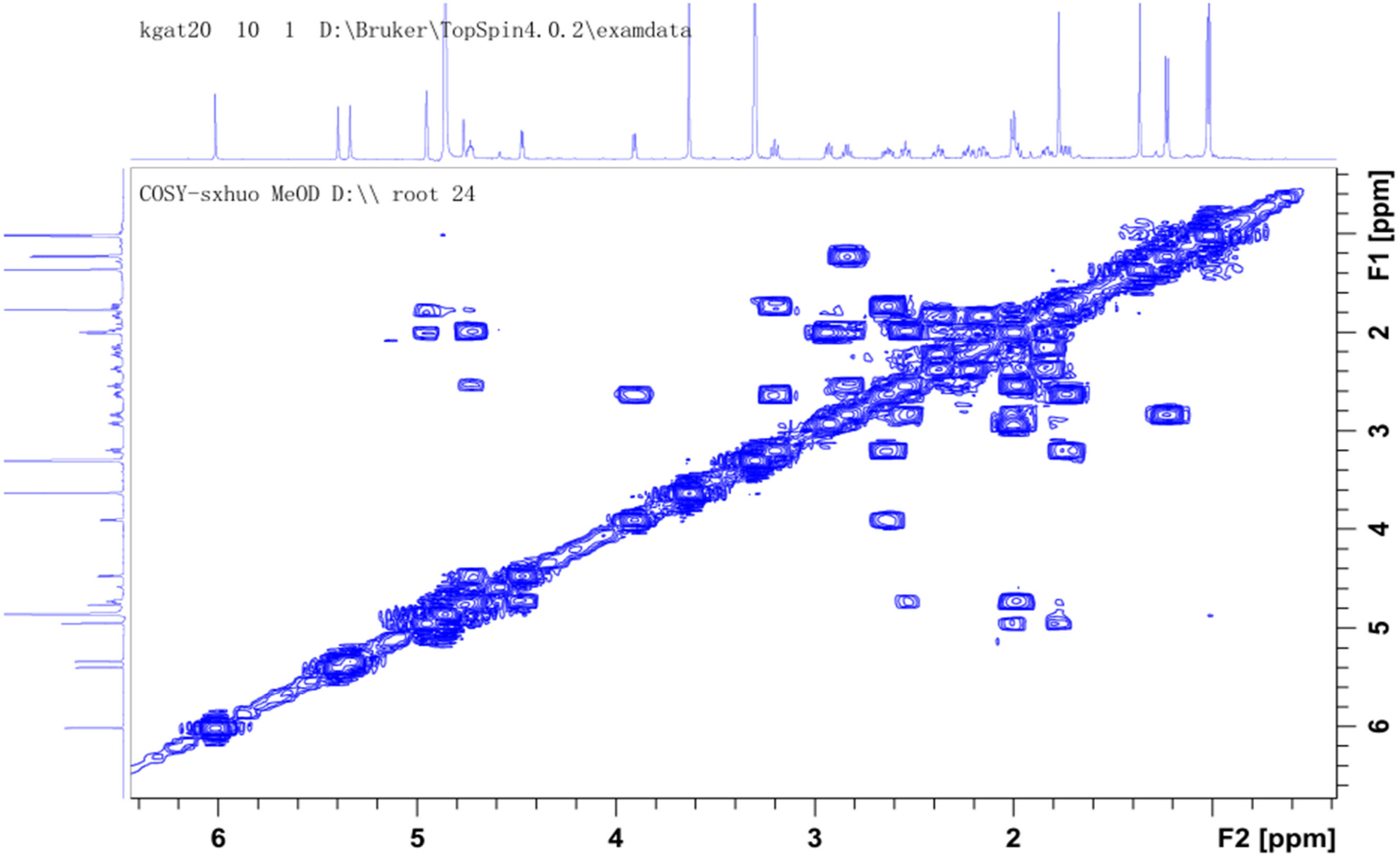

Figure S25. HSQC (600/150 MHz, MeOH-d_4) spectrum of 4.

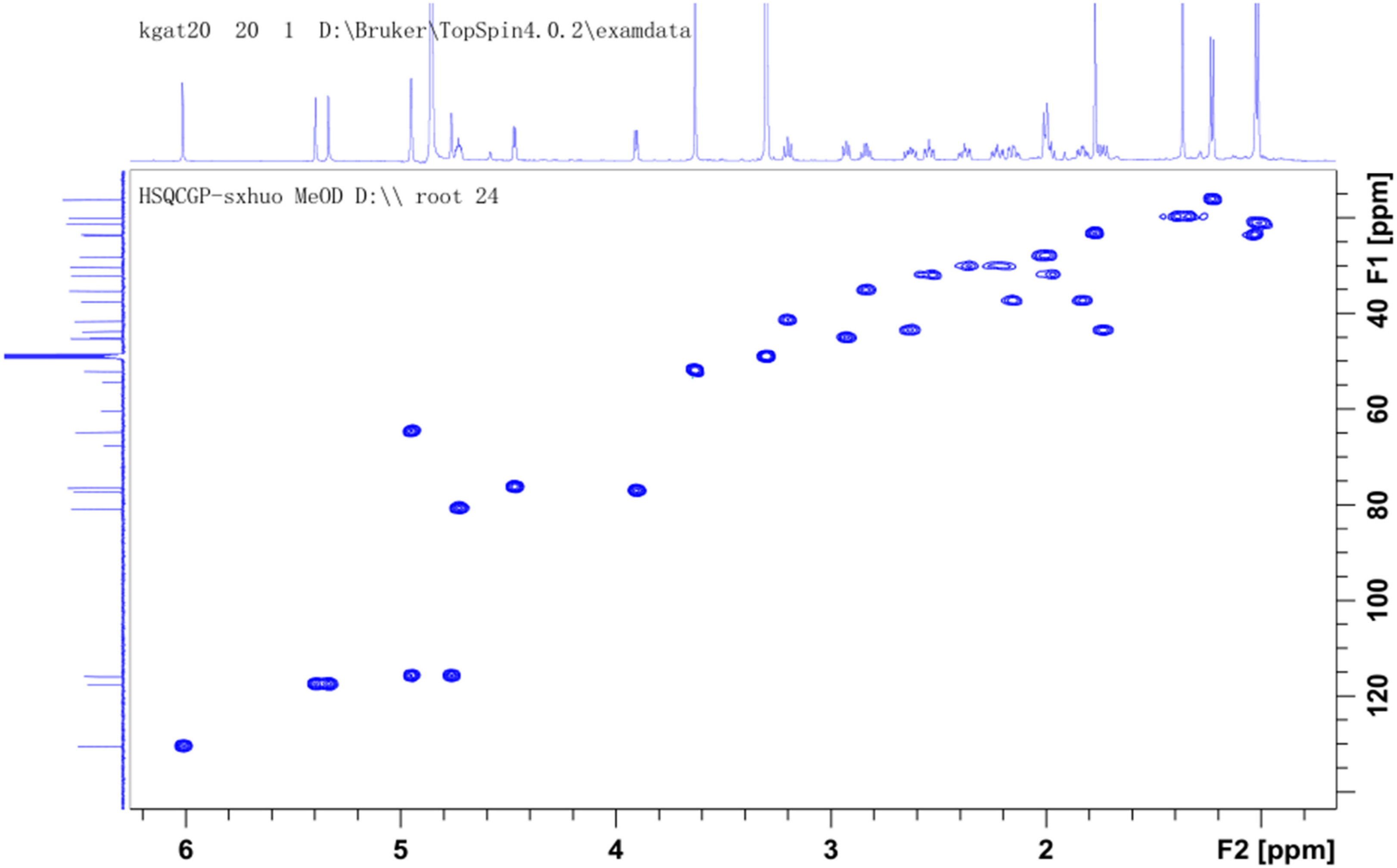

Figure S26. HMBC (600/150 MHz, MeOH-d_4) spectrum of 4.

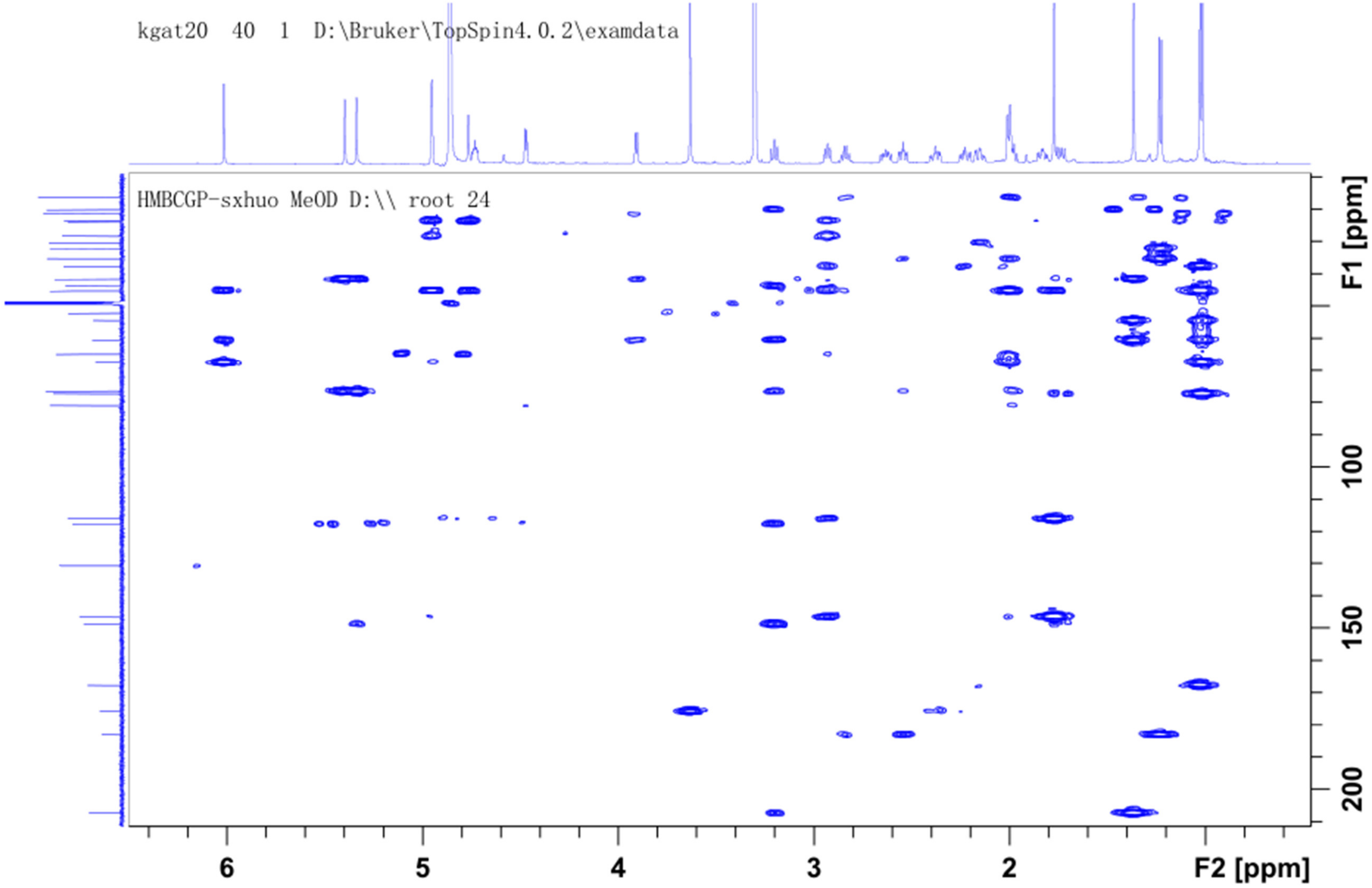

Figure S27. ROESY (600/150 MHz, MeOH-d_4) spectrum of 4.

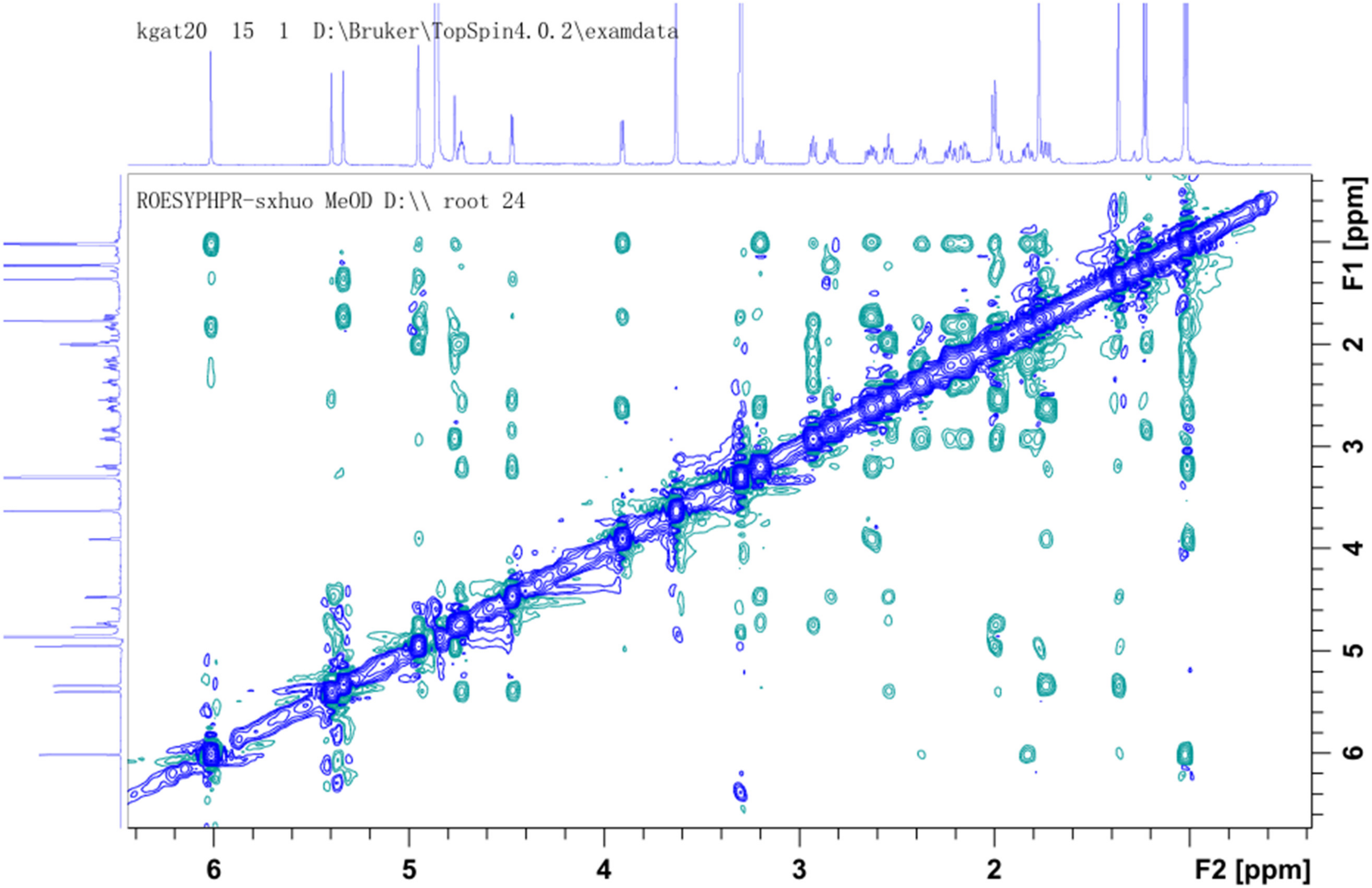

Section S9: HRESIMS spectrum of 4

Figure S28. HRESIMS spectrum of 4.

Qualitative Analysis Report

**Data Filename**	kgat18.d	**Sample Name**	kgat18
**Sample Type**	Sample	**Position**	P1-A7
**Instrument Name**	Instrument 1	**User Name**	
**Acq Method**	s.m	**Acquired Time**	2/25/2021 11:44:06 AM
**IRM Calibration Status**	Success	**DA Method**	Default.m
**Comment**			

Sample Group **Info.**
Acquisition SW Version 6200 series TOF/6500 series Q-TOF B.05.01 (B5125.2)

User Spectra

Fragmentor Voltage	Collision Energy	Ionization Mode
150	0	ESI

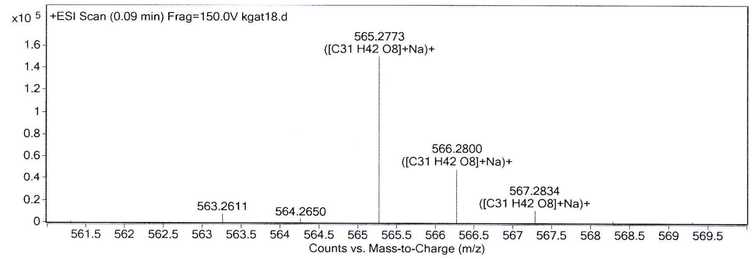

Peak List

m/z	z	Abund	Formula	Ion
102.1273	1	57547.51		
146.0803	1	27420.7		
172.0935	1	43954.79		
535.3023	1	28773.45		
543.2954	1	32063.38		
565.2773	1	150467.63	C31 H42 O8	(M+Na)+
566.28	1	49360.54	C31 H42 O8	(M+Na)+
714.3826	1	25285.37		
1107.565	1	43101.66		
1108.5688	1	28253.28		

Formula Calculator Element Limits

Element	Min	Max
C	3	60
H	0	120
O	0	30

Formula Calculator Results

Formula	CalculatedMass	CalculatedMz	Mz	Diff. (mDa)	Diff. (ppm)	DBE
C31 H42 O8	542.2880	565.2772	565.2773	-0.10	-0.18	11.0000

--- End Of Report ---

Agilent Technologies Page 1 of 1 Printed at: 2:01 PM on: 2/25/2021

Section S10: Comparison of ^{1}H NMR and ^{1}H-^{1}H COSY spectra between 4r and 4s

Figure S29. Comparison of ^{1}H NMR spectra (600 MHz, pyridine-d_5) between 4r and 4s.

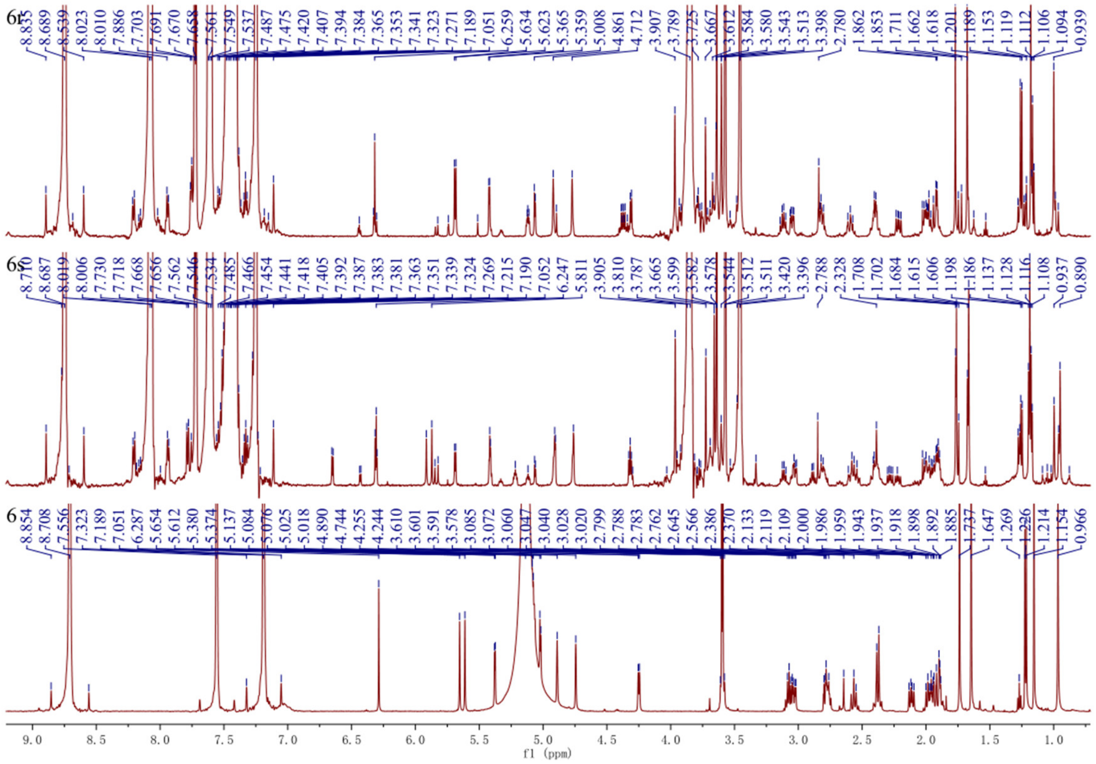

Figure S30. The ^1H-^1H COSY spectrum (600 MHz, pyridine-d_5) of 4r.

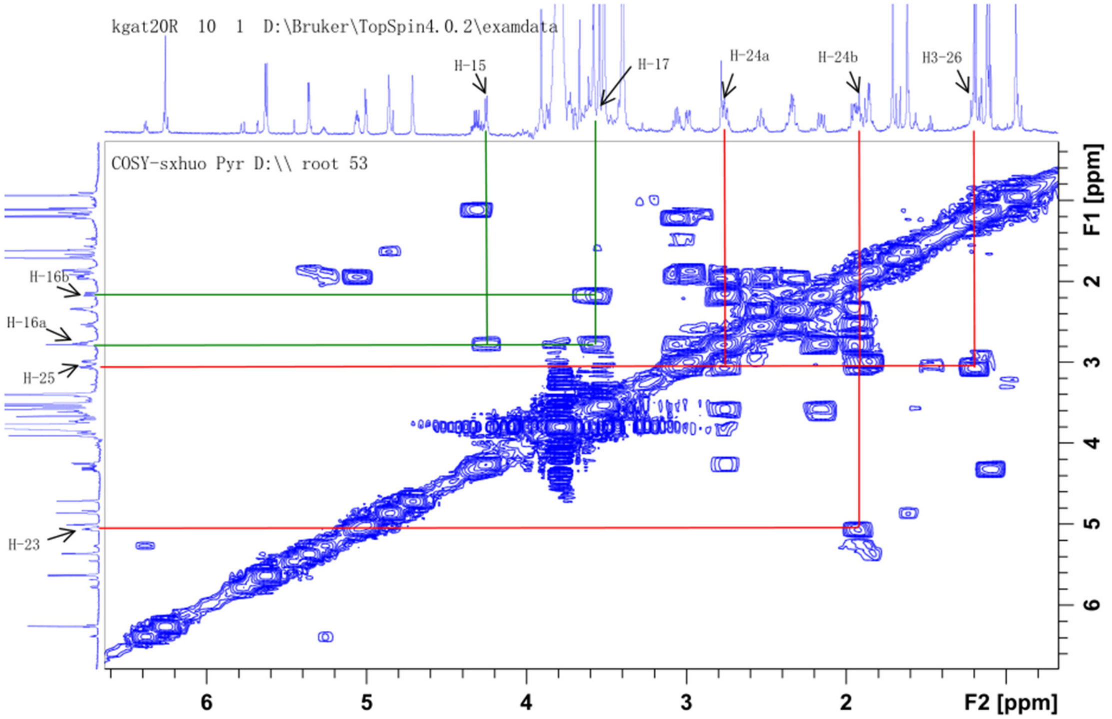

Figure S31. The ^{1}H-^{1}H COSY spectrum (600 MHz, pyridine-d_5) of 4r.

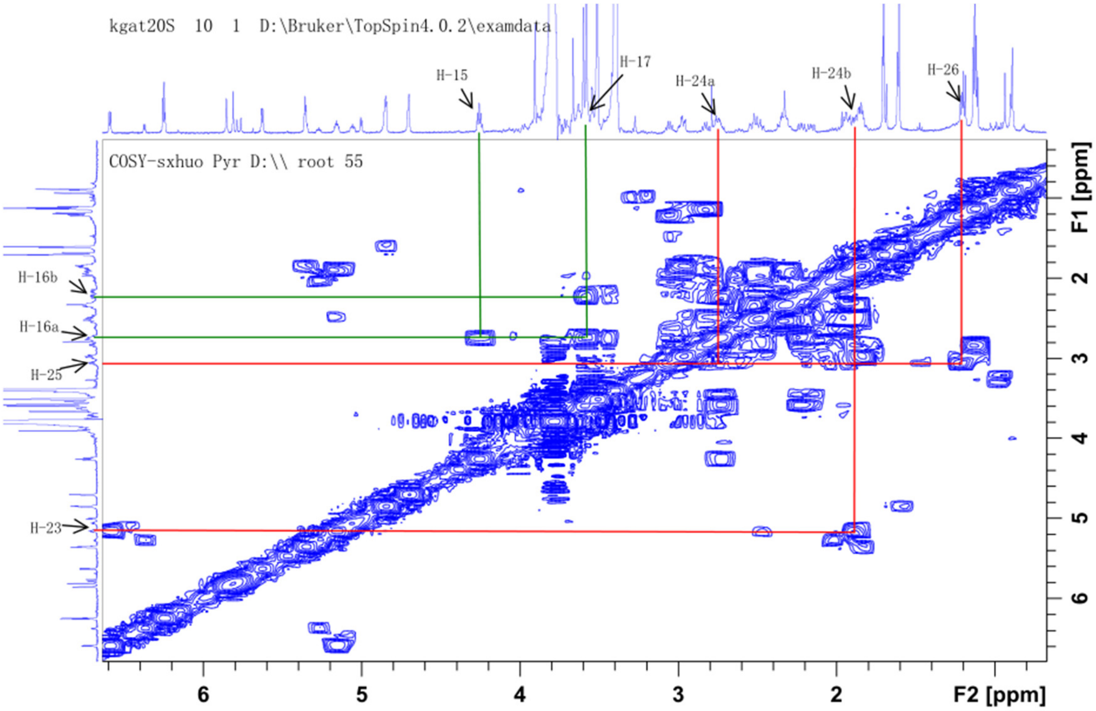

Section S11: 1D and 2D NMR spectra of compound 5

Figure S32. ^{1}**H NMR (600 MHz, MeOH-d_4) spectrum of 5.**

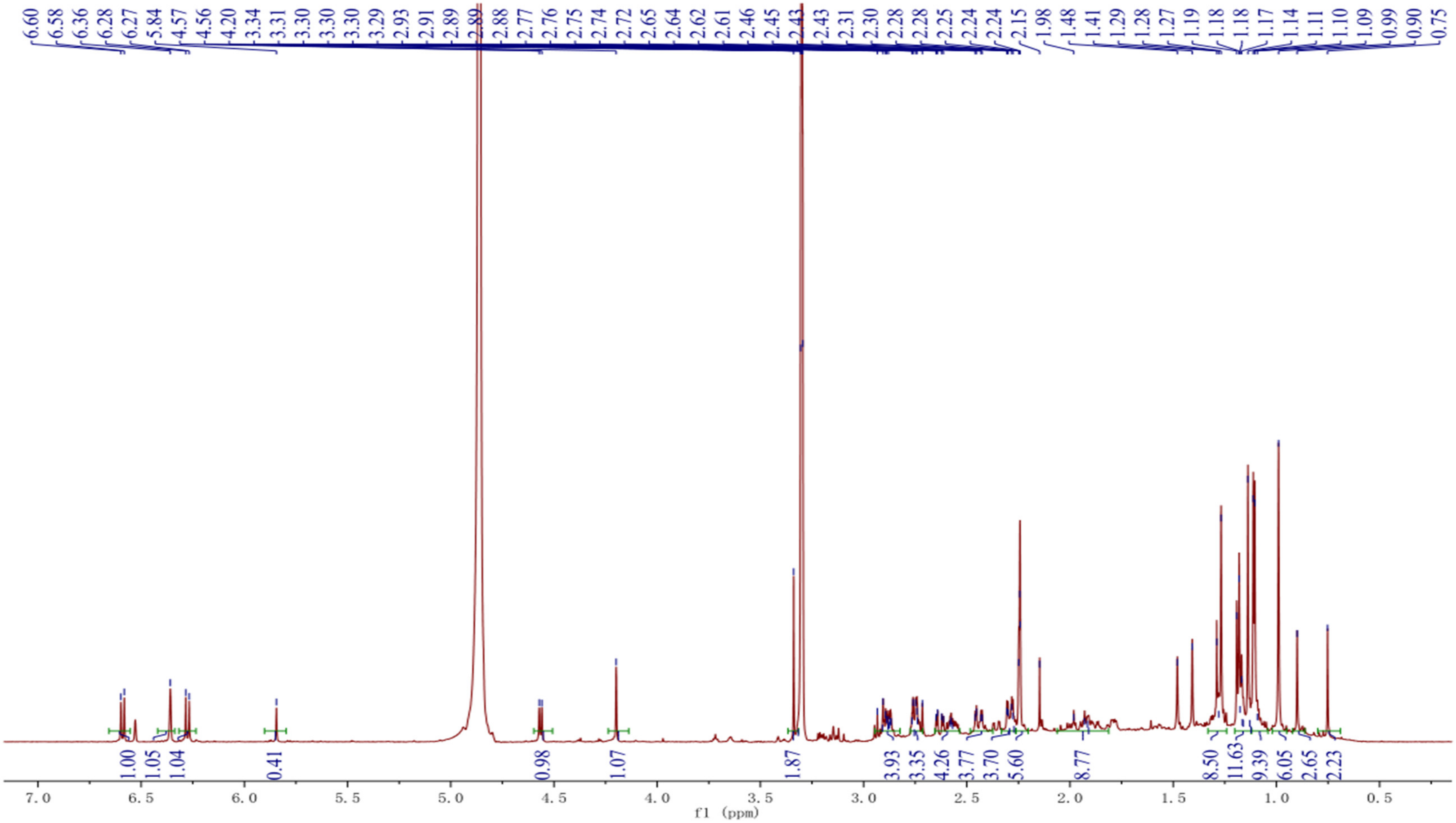

Figure S33. ^{13}**C NMR (150 MHz, MeOH-*d*$_4$) spectrum of 5.**

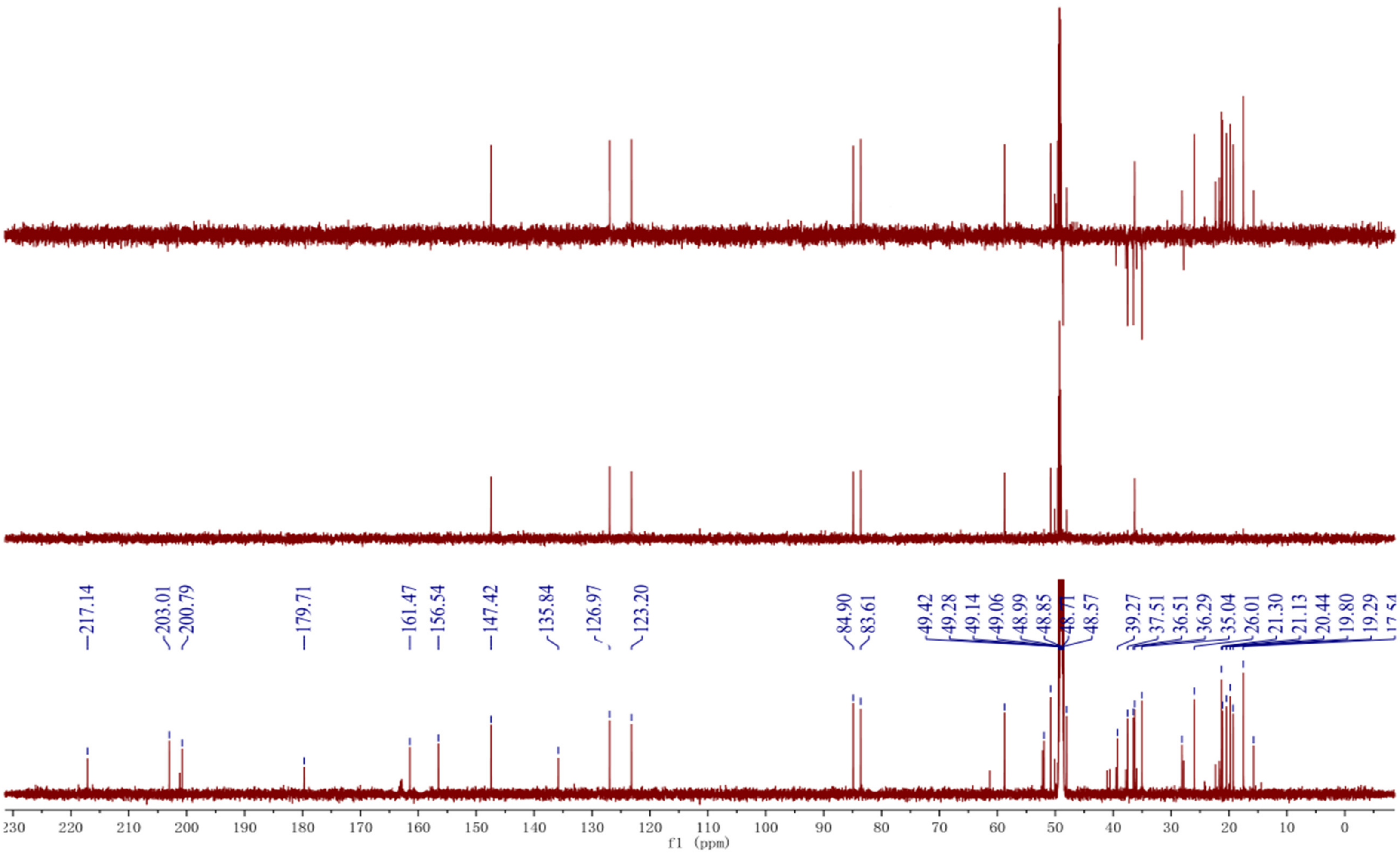

Figure S34. ^{1}H-^{1}H COSY (600 MHz, MeOH-d_4) spectrum of **5**.

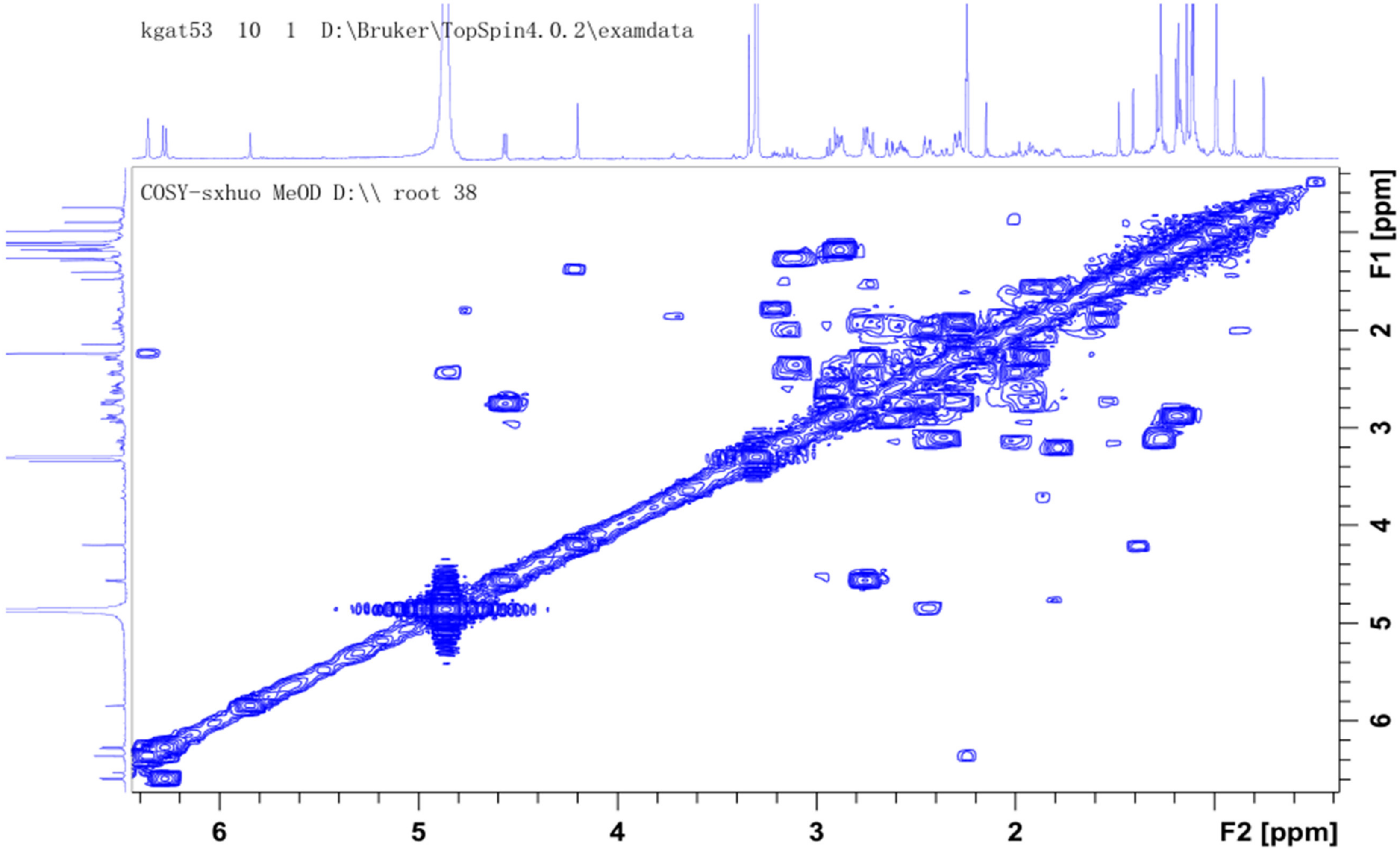

Figure S35. HSQC (600/150 MHz, MeOH-d_4) spectrum of 5.

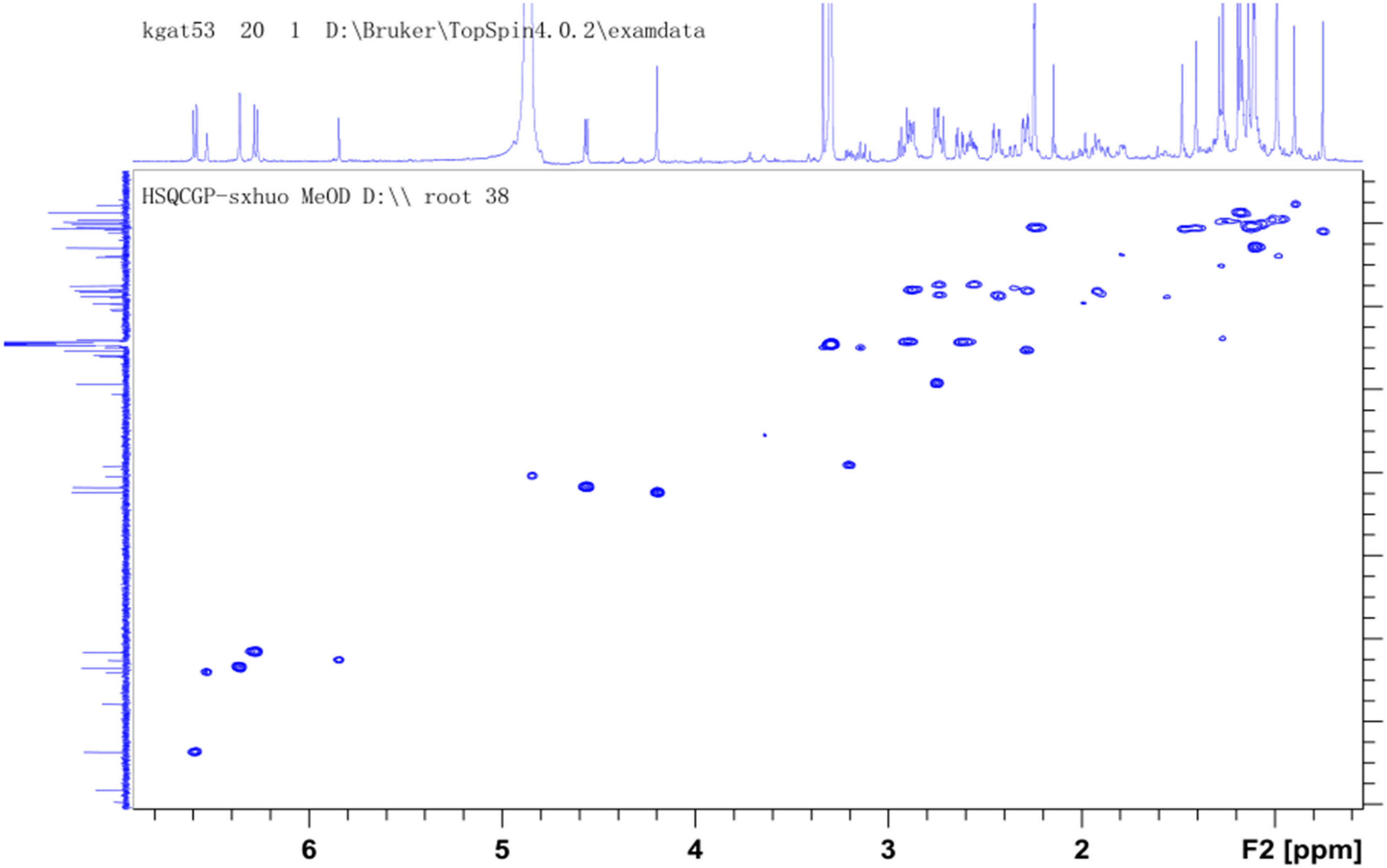

Figure S36. HMBC (600/150 MHz, MeOH-d_4) spectrum of 5.

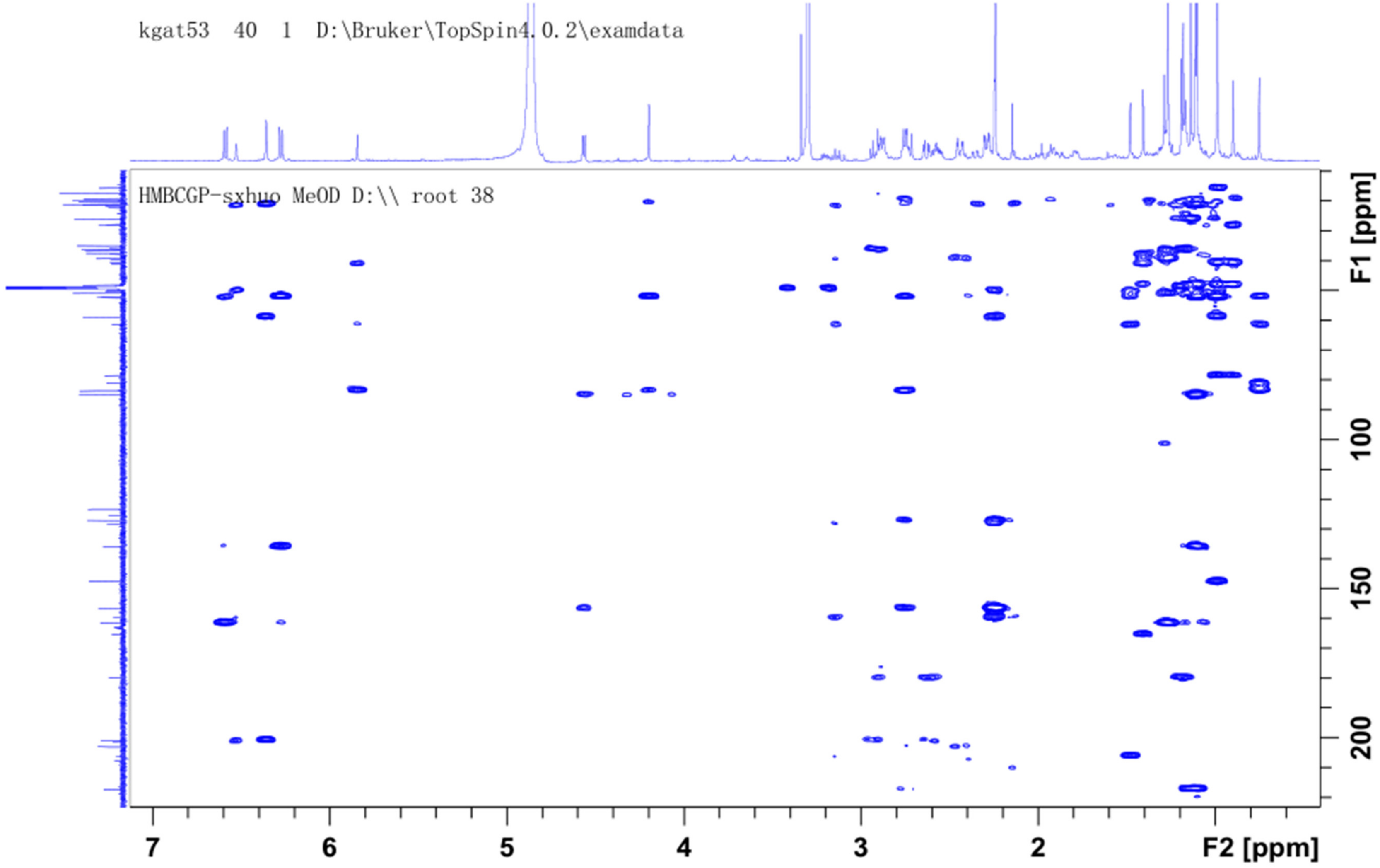

Figure S37. ROESY (600/150 MHz, MeOH-d_4) spectrum of 5.

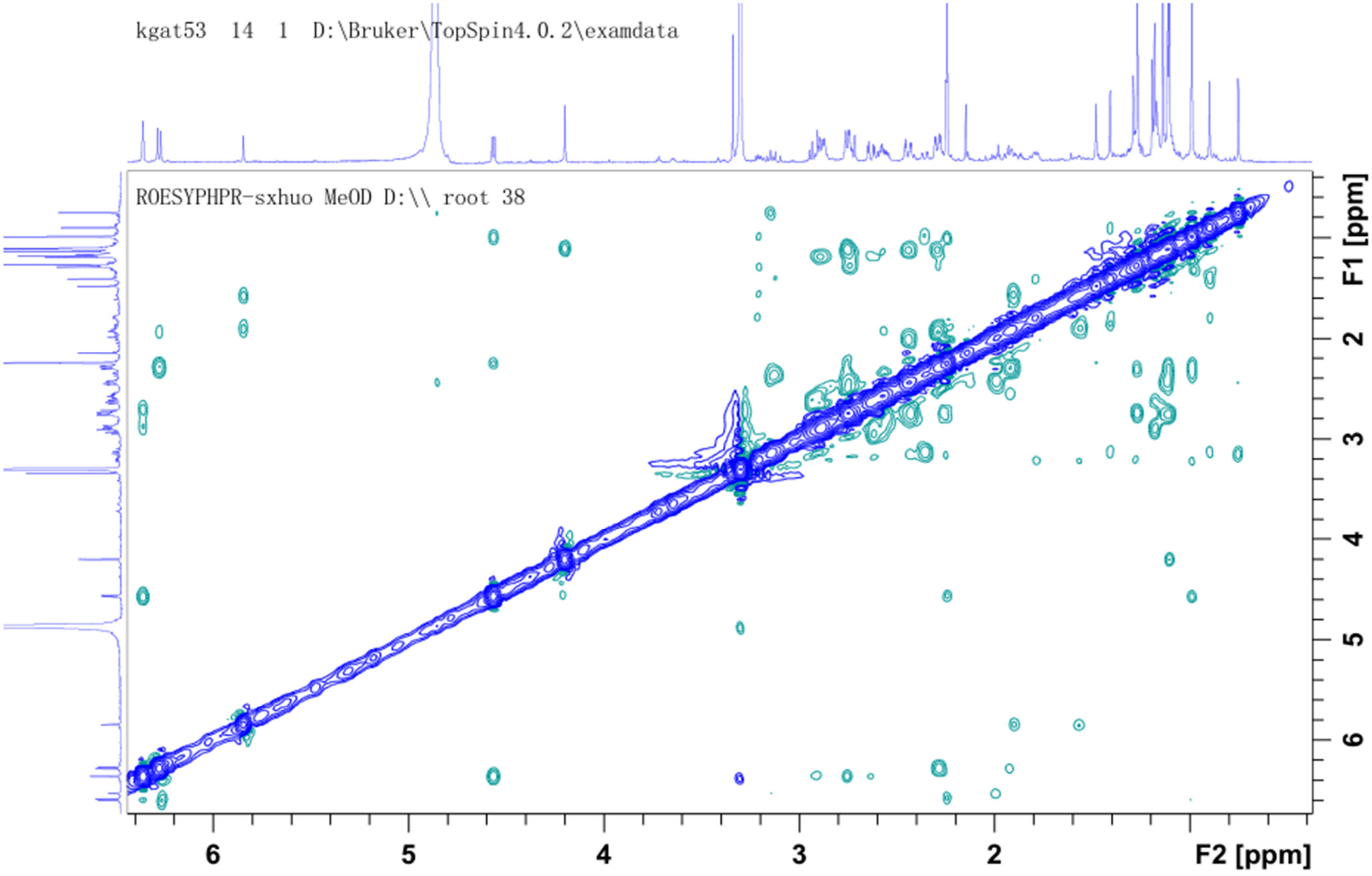

Section S12: HRESIMS spectrum of 5

Figure S38. HRESIMS spectrum of 5.

Qualitative Analysis Report

**Data Filename**	kgat53.d	**Sample Name**	kgat53
**Sample Type**	Sample	**Position**	P1-A2
**Instrument Name**	Instrument 1	**User Name**	
**Acq Method**	s-.m	**Acquired Time**	7/9/2021 2:52:30 PM
**IRM Calibration Status**	Success	**DA Method**	Default.m
**Comment**			

Sample Group **Info.**
Acquisition SW 6200 series TOF/6500 series
Version Q-TOF B.05.01 (B5125.2)

User Spectra

Fragmentor Voltage	Collision Energy	Ionization Mode
180	0	ESI

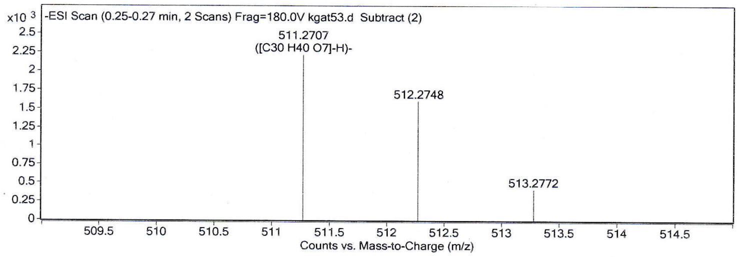

Peak List

m/z	z	Abund	Formula	Ion
113.9895		422.89		
511.2707		2213.88	C30 H40 O7	(M-H)-
512.2748	1	1590.76		
513.2772	1	409.76		
515.3022		448.46		
525.2506		447.94		
527.2635	1	789.87		
529.281		2487.08		
530.2848	1	1967.59		
551.2611		398.29		
579.2579	1	496.07		
643.2744	1	433.25		

Formula Calculator Element Limits

Element	Min	Max
C	3	60
H	0	120
O	0	30

Formula Calculator Results

Formula	CalculatedMass	CalculatedMz	Mz	Diff. (mDa)	Diff. (ppm)	DBE
C30 H40 O7	512.2774	511.2701	511.2707	-0.60	-1.17	11.0000

--- End Of Report ---

Agilent Technologies Page 1 of 1 Printed at: 3:12 PM on: 7/9/2021

Section S13: 1D and 2D NMR spectra of compound 6

Figure S39. ^{1}H **NMR (600 MHz, MeOH-d_4) spectrum of 6.**

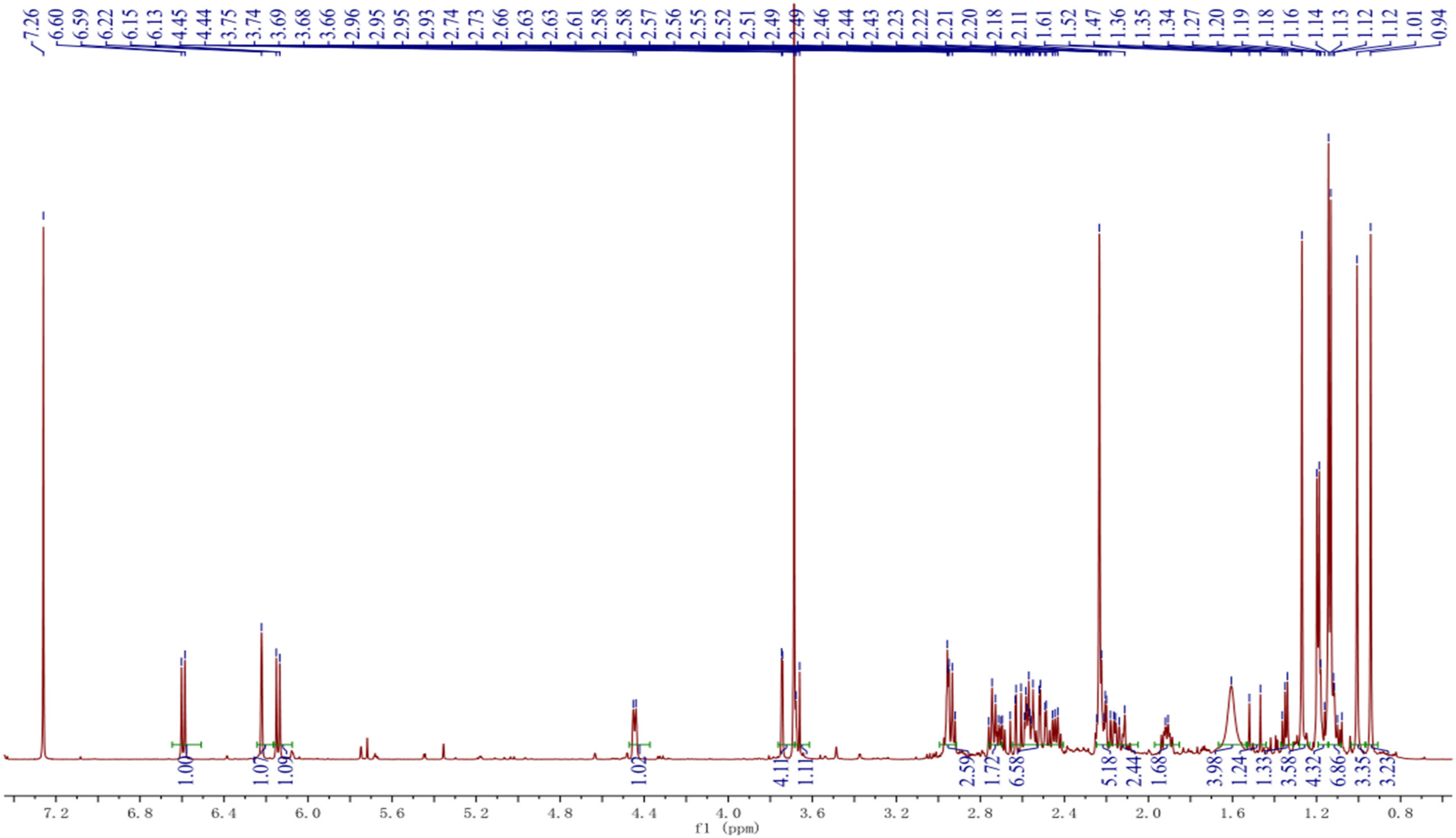

Figure S40. ^{13}C **NMR (150 MHz, MeOH-*d*$_4$) spectrum of 6.**

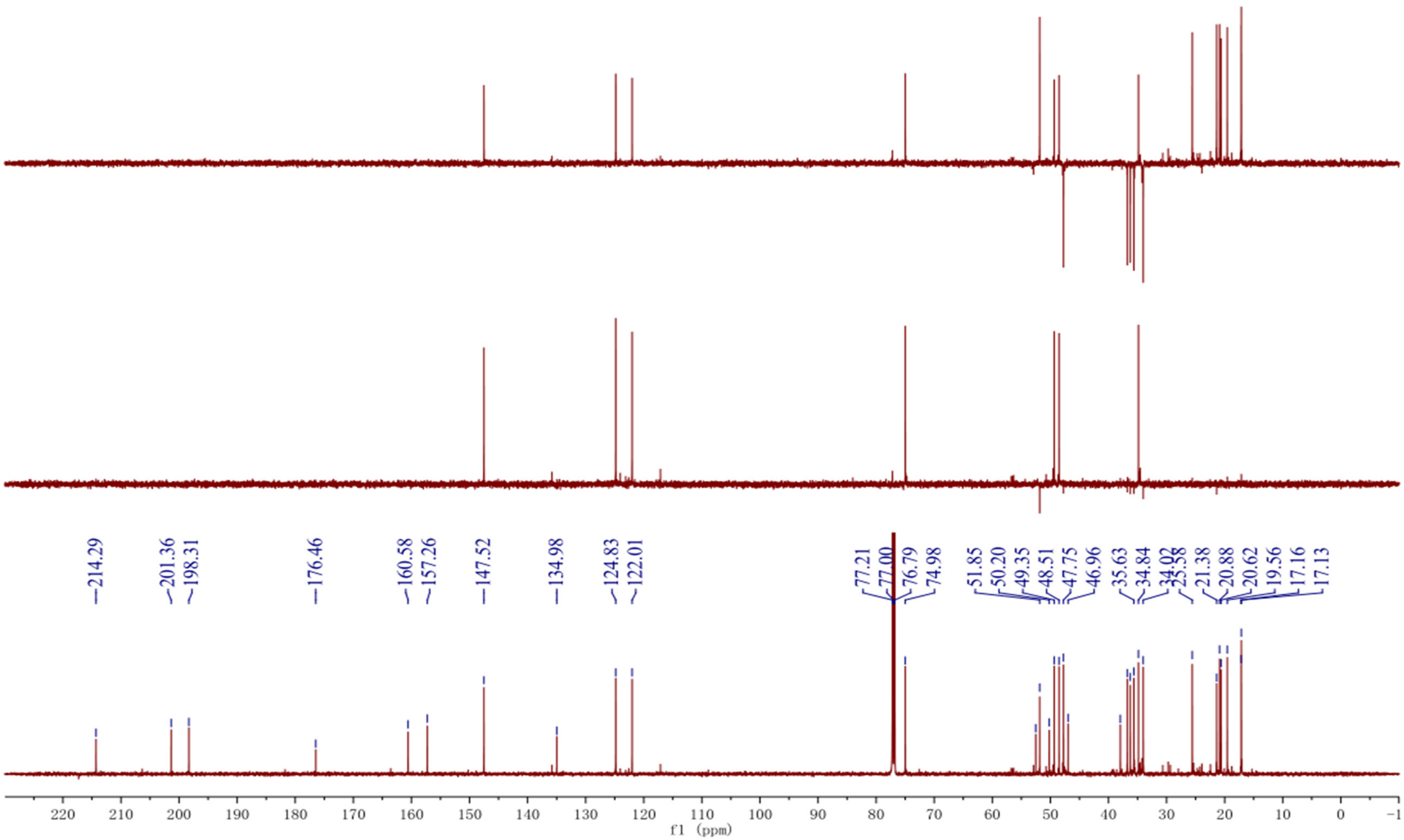

Figure S41. ^{1}H-^{1}H **COSY (600 MHz, MeOH-*d*$_4$) spectrum of 6.**

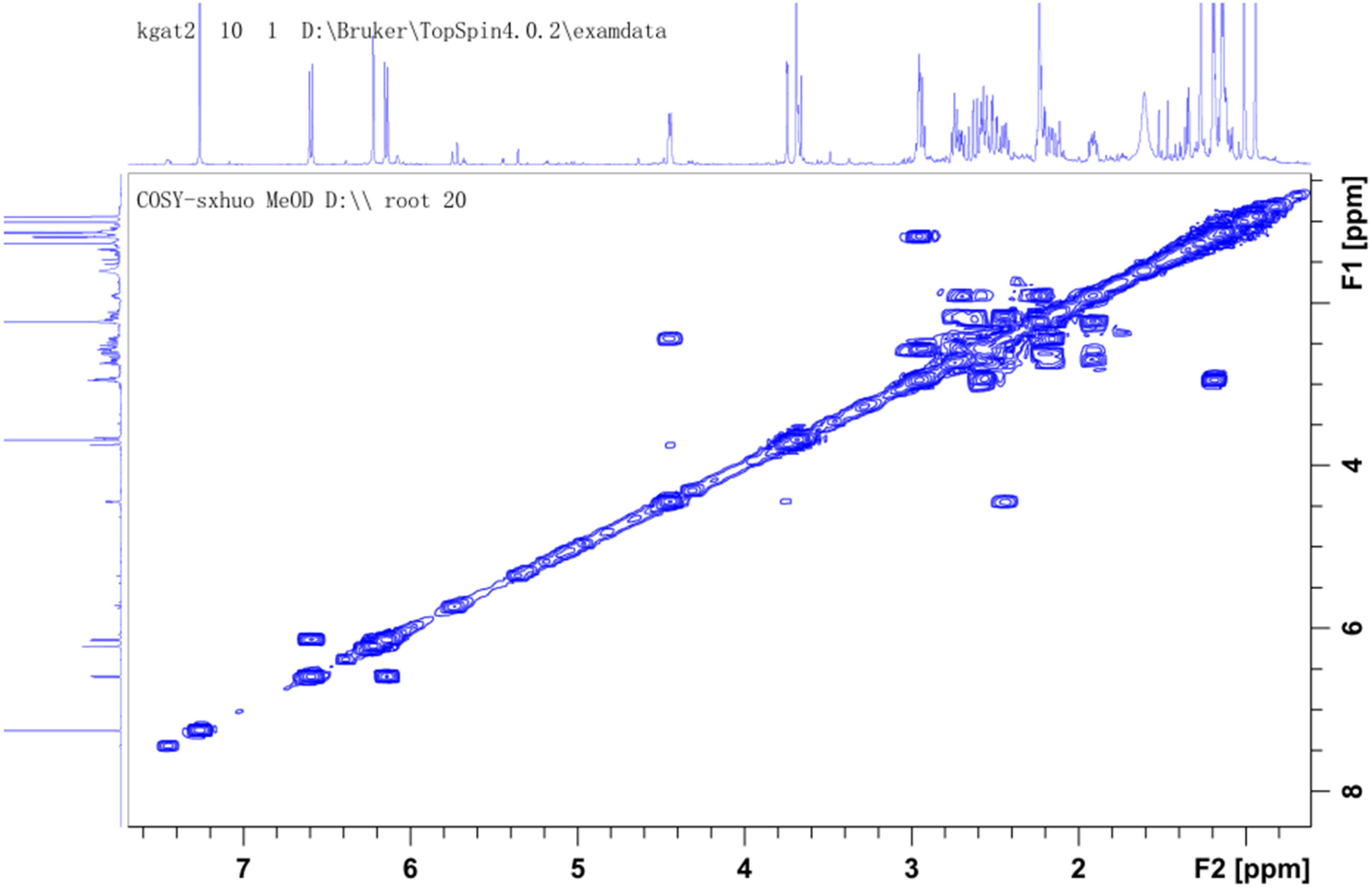

Figure S42. HSQC (600/150 MHz, MeOH-d_4) spectrum of 6.

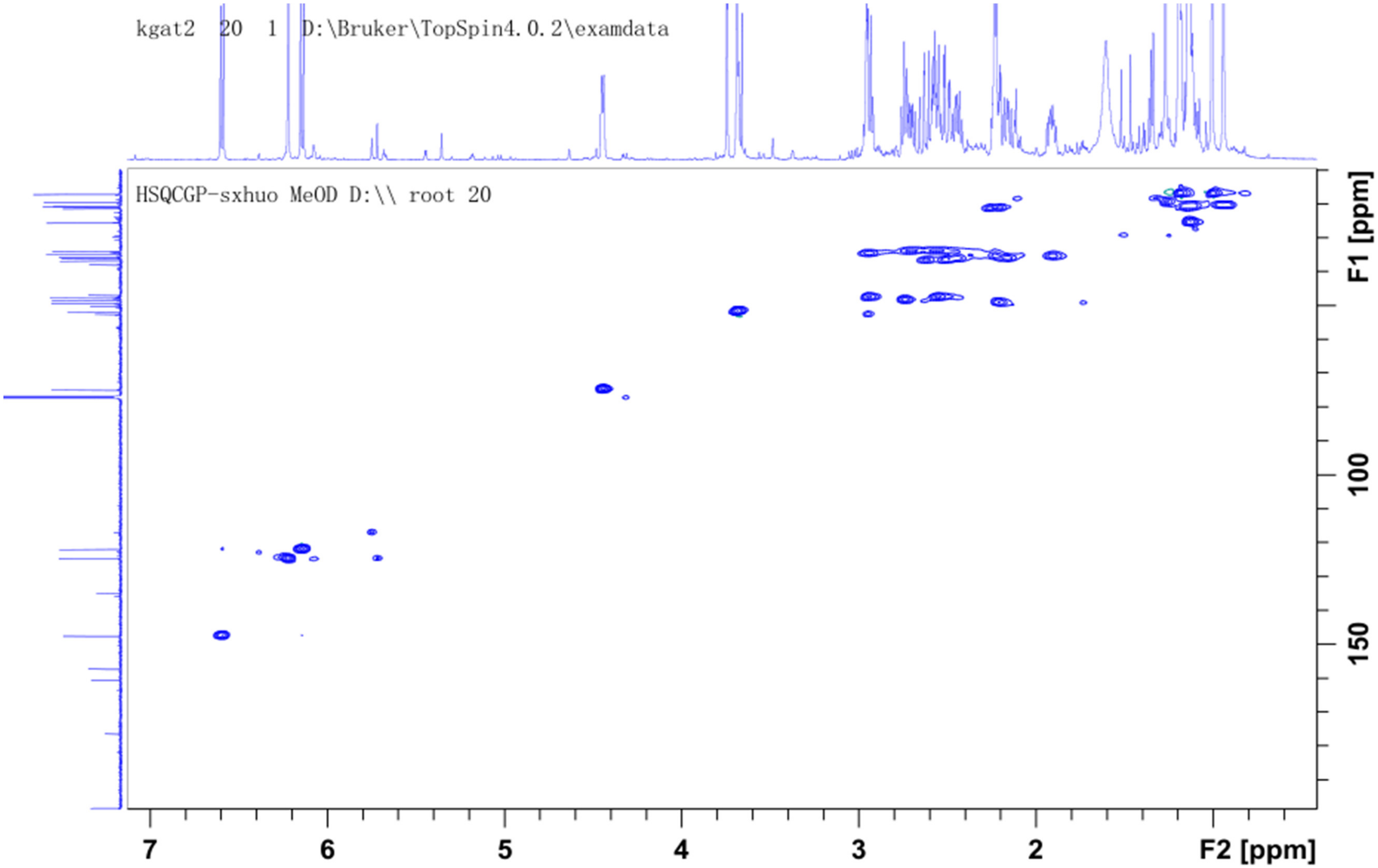

Figure S43. HMBC (600/150 MHz, MeOH-d_4) spectrum of 6.

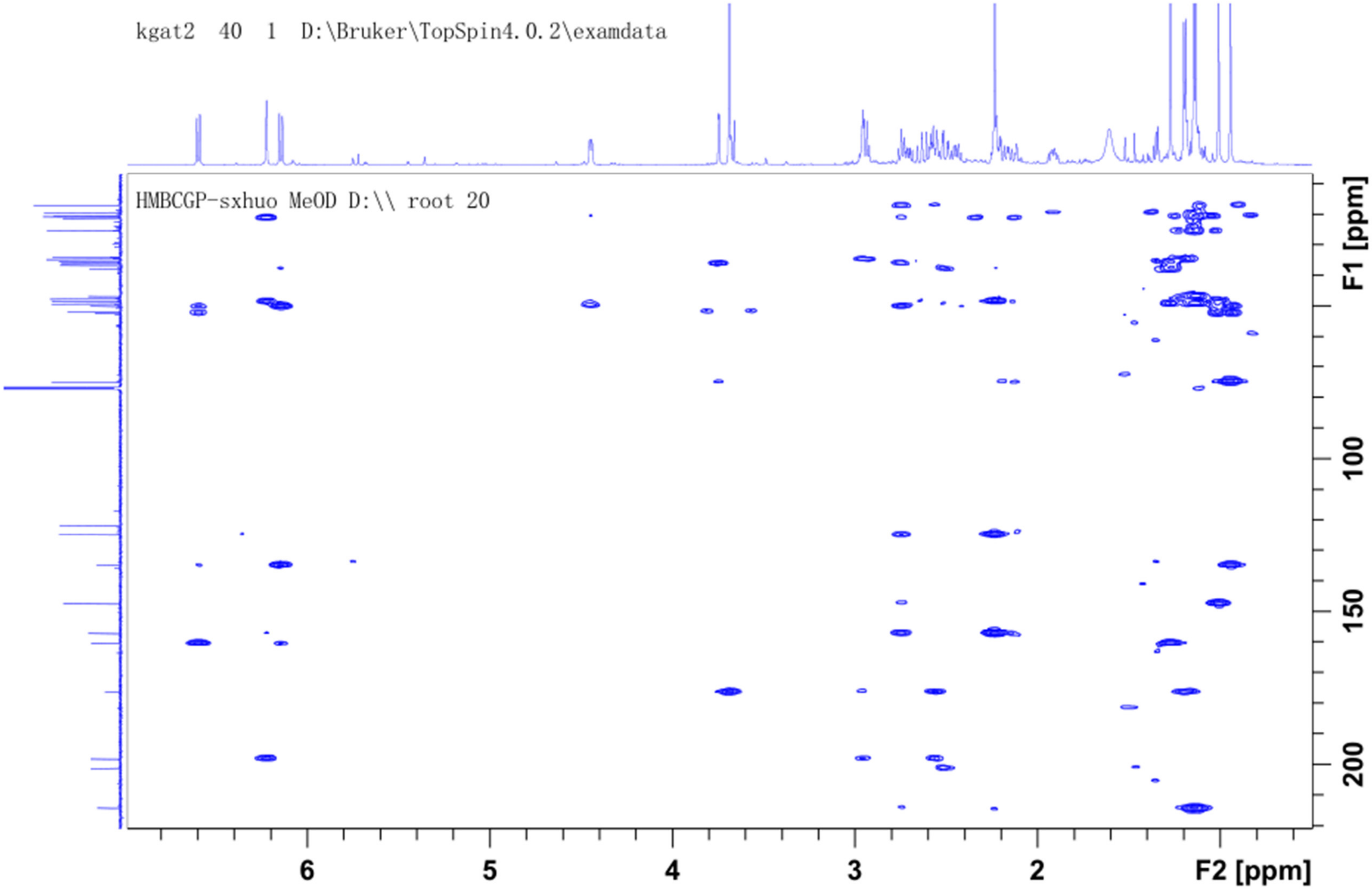

Figure S44. ROESY (600/150 MHz, MeOH-d_4) spectrum of 6.

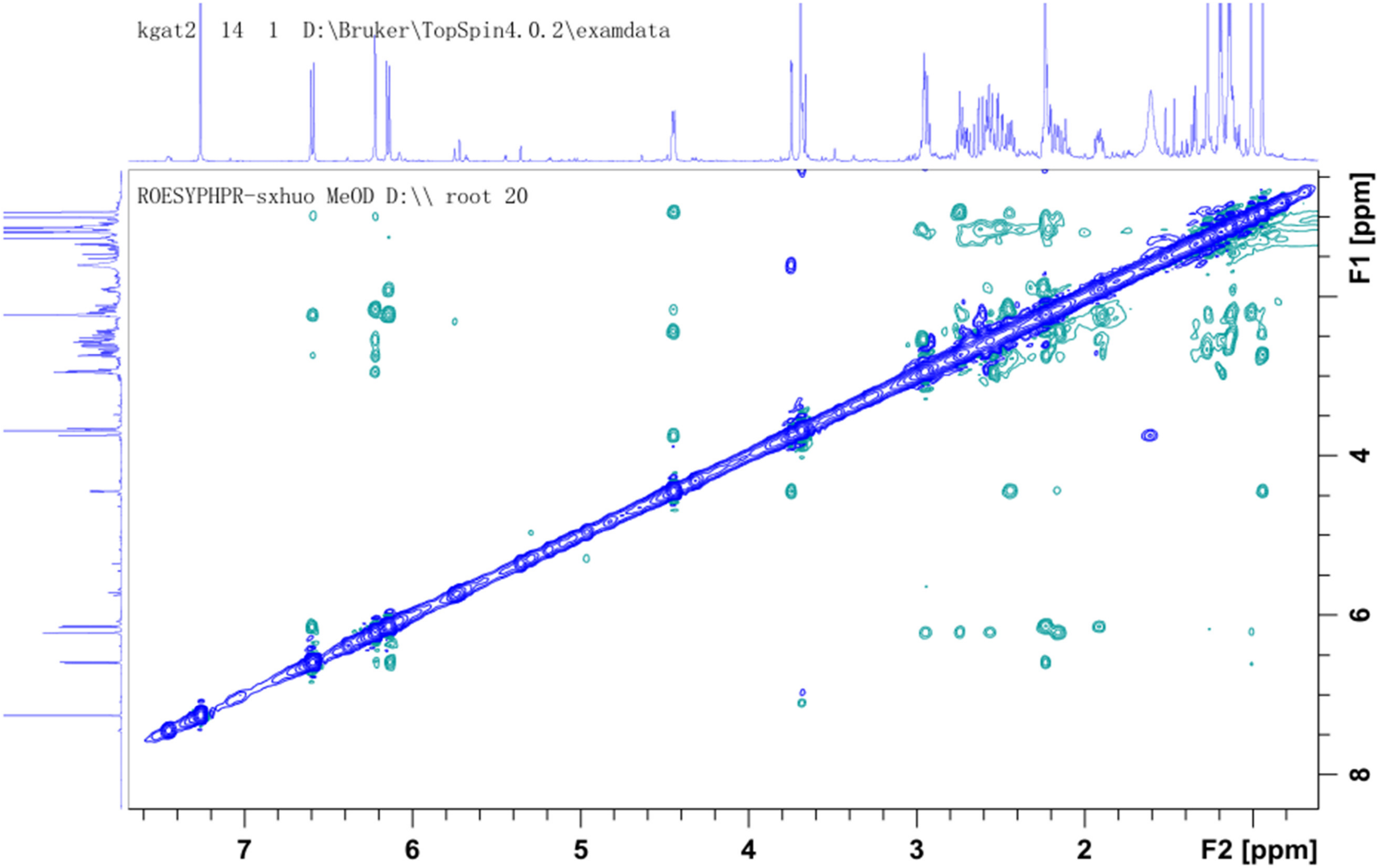

Figure S45. HRESIMS spectrum of 6.

Qualitative Analysis Report

**Data Filename**	kgat2.d	**Sample Name**	kgat2
**Sample Type**	Sample	**Position**	P1-A3
**Instrument Name**	Instrument 1	**User Name**	
**Acq Method**	s.m	**Acquired Time**	2/25/2021 11:39:22 AM
**IRM Calibration Status**	Success	**DA Method**	Default.m
**Comment**			

Sample Group **Info.**
Acquisition SW Version 6200 series TOF/6500 series Q-TOF B.05.01 (B5125.2)

User Spectra

Fragmentor Voltage	Collision Energy	Ionization Mode
150	0	ESI

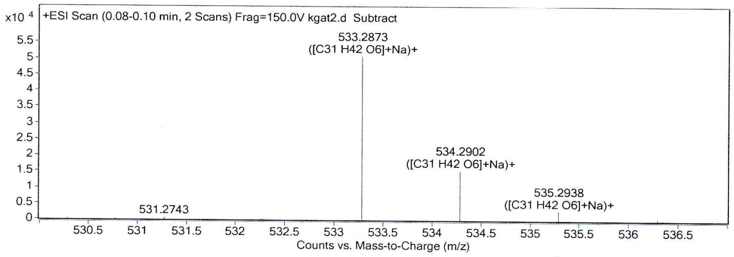

Peak List

m/z	z	Abund	Formula	Ion
102.1274	1	37320.11		
511.3058	1	14331.96		
533.2873	1	50725.95	C31 H42 O6	(M+Na)+
534.2902	1	15496.51	C31 H42 O6	(M+Na)+
547.2663	1	16946.77		
549.27	1	16798.7		
1043.5859	1	26370.52		
1044.5892	1	16678.78		
1057.5643	1	10931.84		
1059.5744	1	7625.36		

Formula Calculator Element Limits

Element	Min	Max
C	3	60
H	0	120
O	0	30

Formula Calculator Results

Formula	CalculatedMass	CalculatedMz	Mz	Diff. (mDa)	Diff. (ppm)	DBE
C31 H42 O6	510.2981	533.2874	533.2873	0.10	0.19	11.0000

--- End Of Report ---

Agilent Technologies Page 1 of 1 Printed at: 1:58 PM on: 2/25/2021

Section S15: 1D and 2D NMR spectra of compound 7

Figure S46. ^{1}H NMR (600 MHz, MeOH-d_4) spectrum of 7.

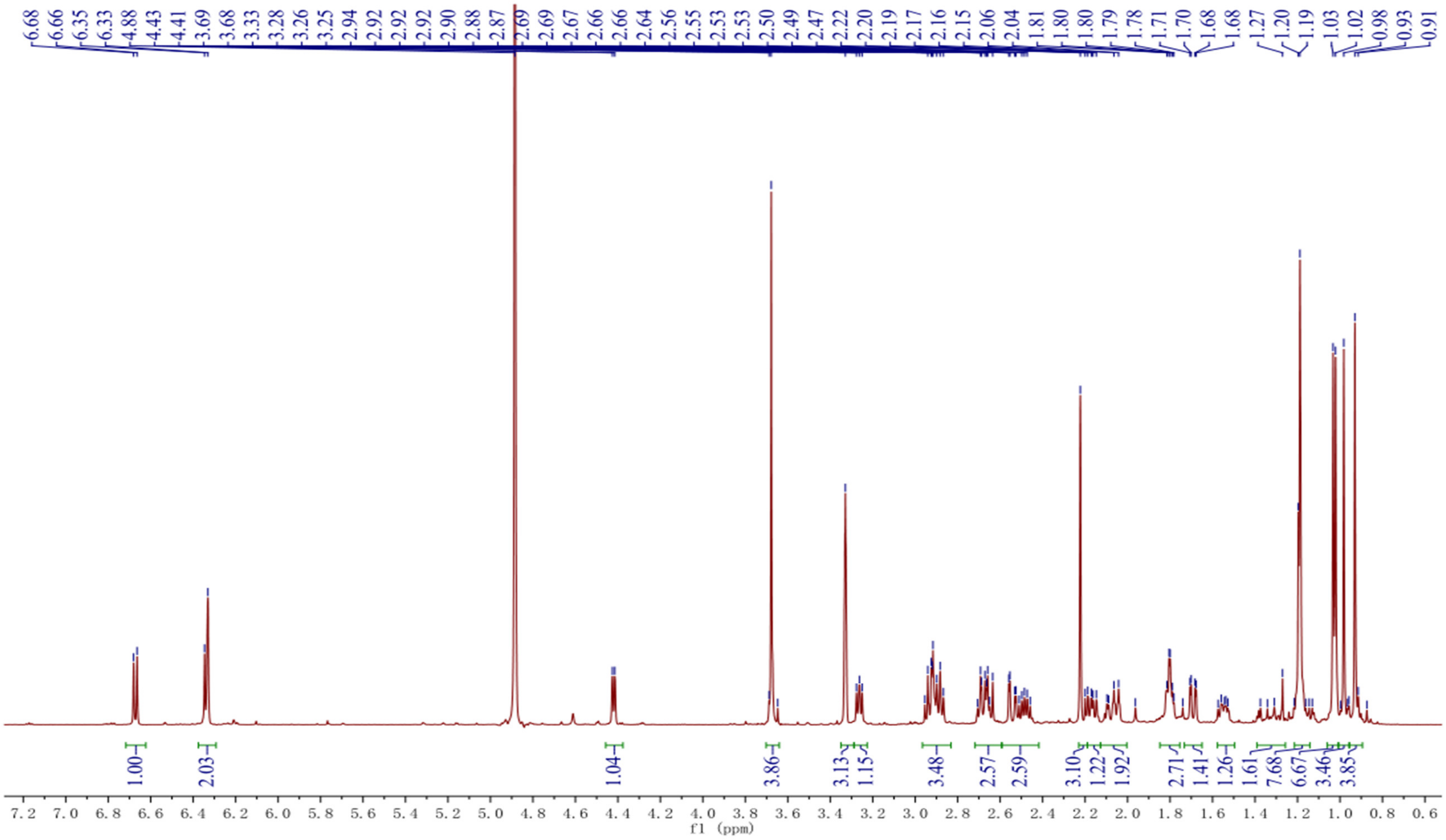

Figure S47. ^{13}**C NMR (150 MHz, MeOH-*d*$_4$) spectrum of 7.**

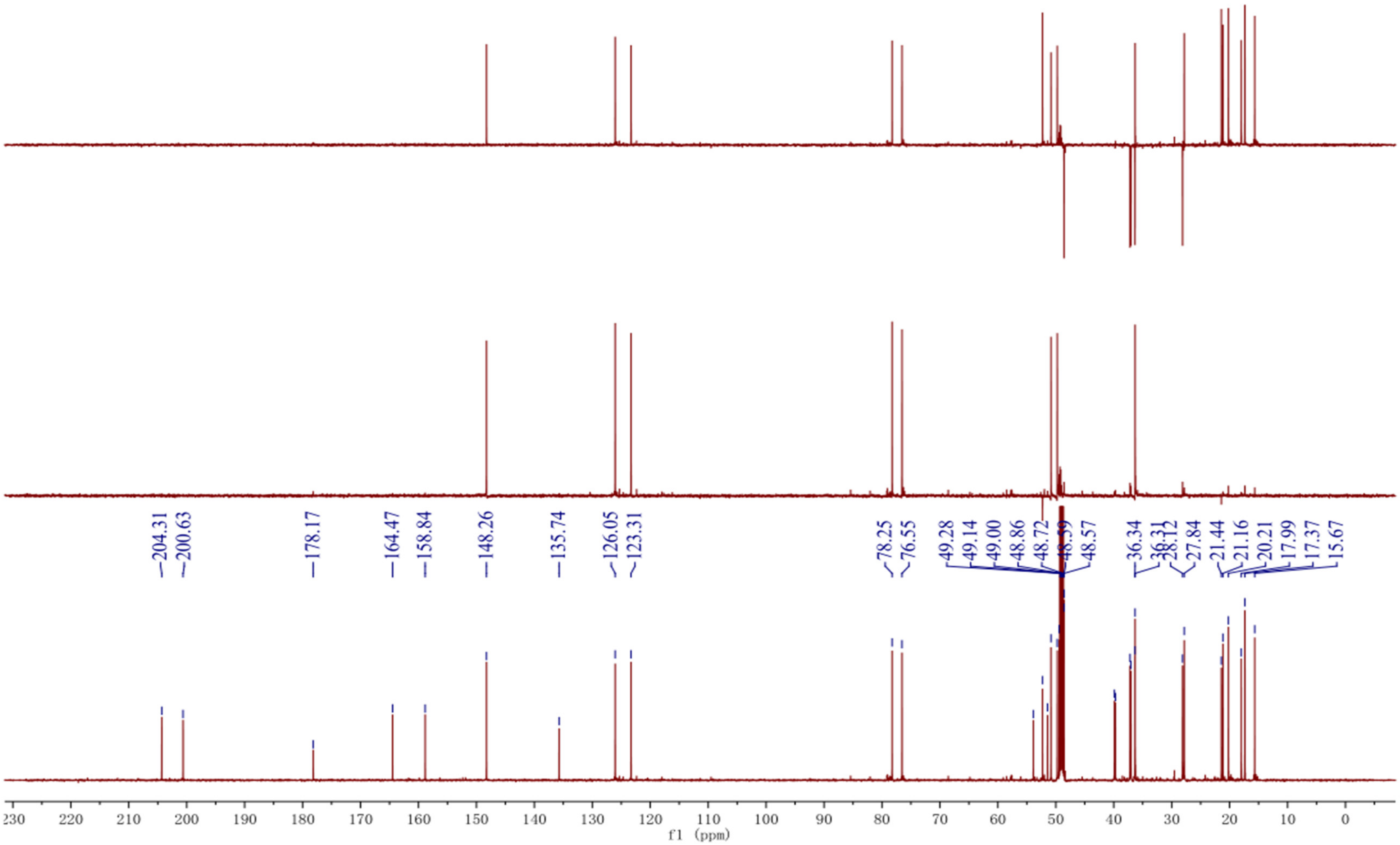

Figure S48. ^{1}H-^{1}H COSY (600 MHz, MeOH-d_4) spectrum of 7.

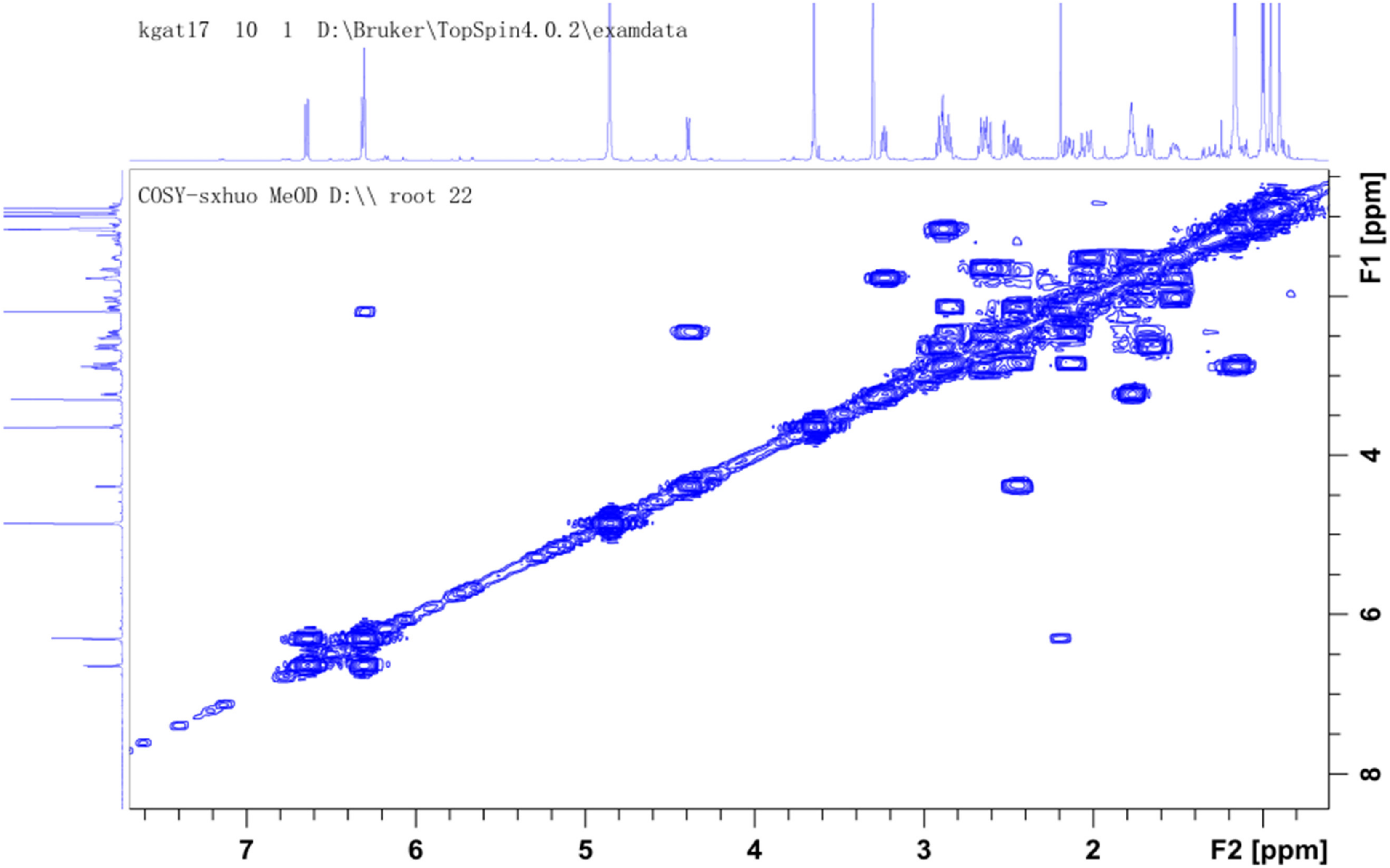

Figure S49. HSQC (600/150 MHz, MeOH-d_4) spectrum of 7.

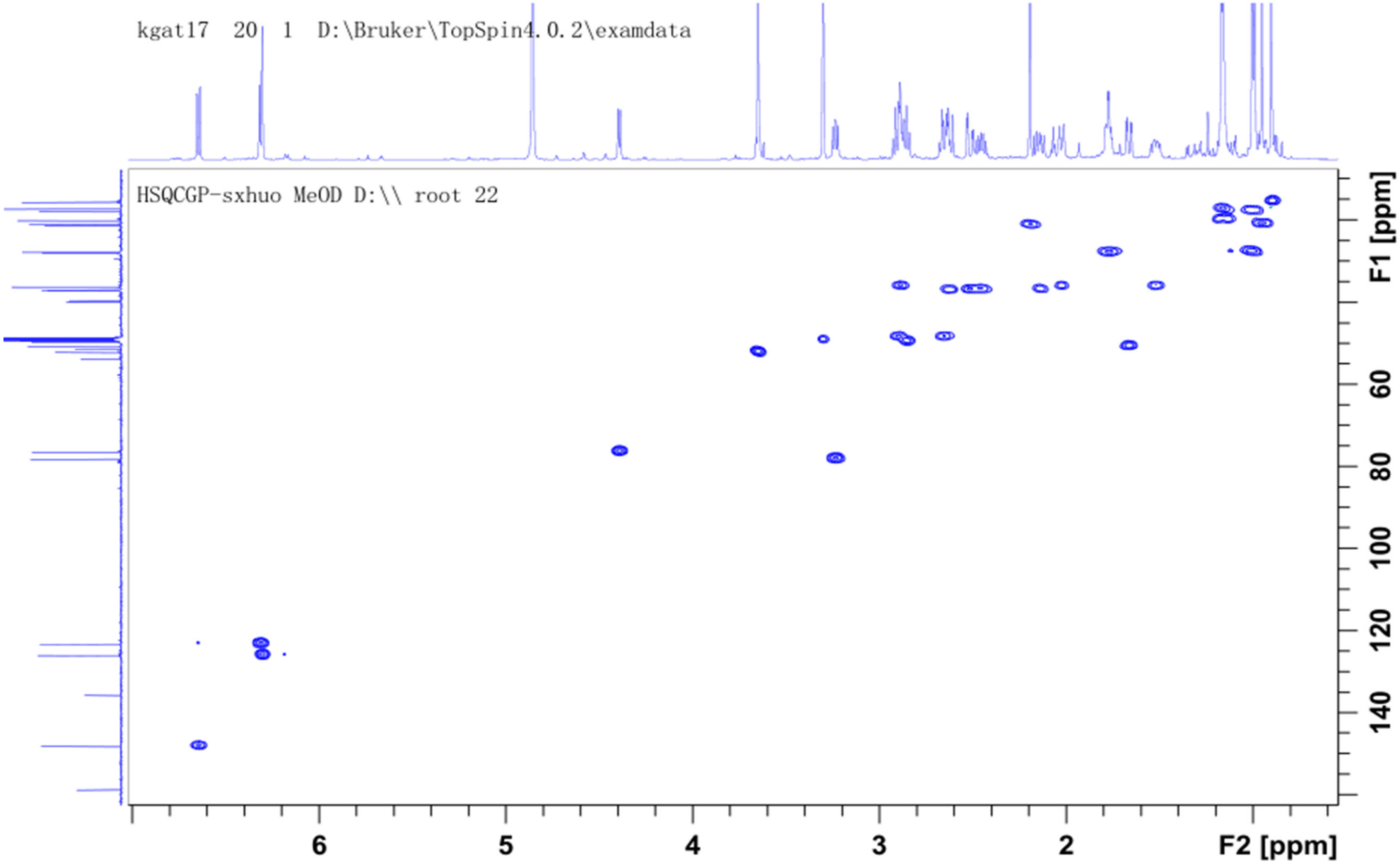

Figure S50. HMBC (600/150 MHz, MeOH-d_4) spectrum of 7.

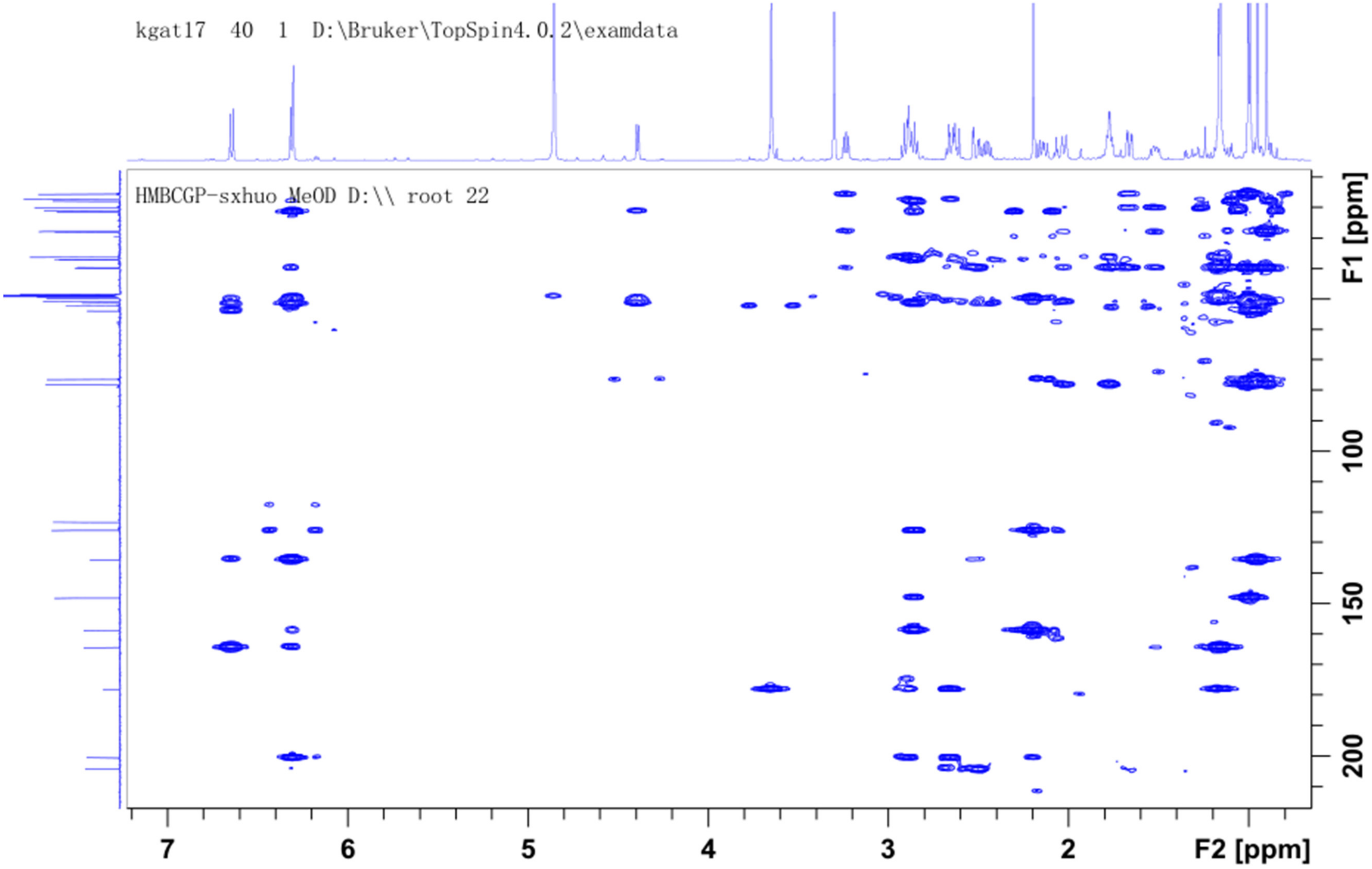

Figure S51. ROESY (600/150 MHz, MeOH-d_4) spectrum of 7.

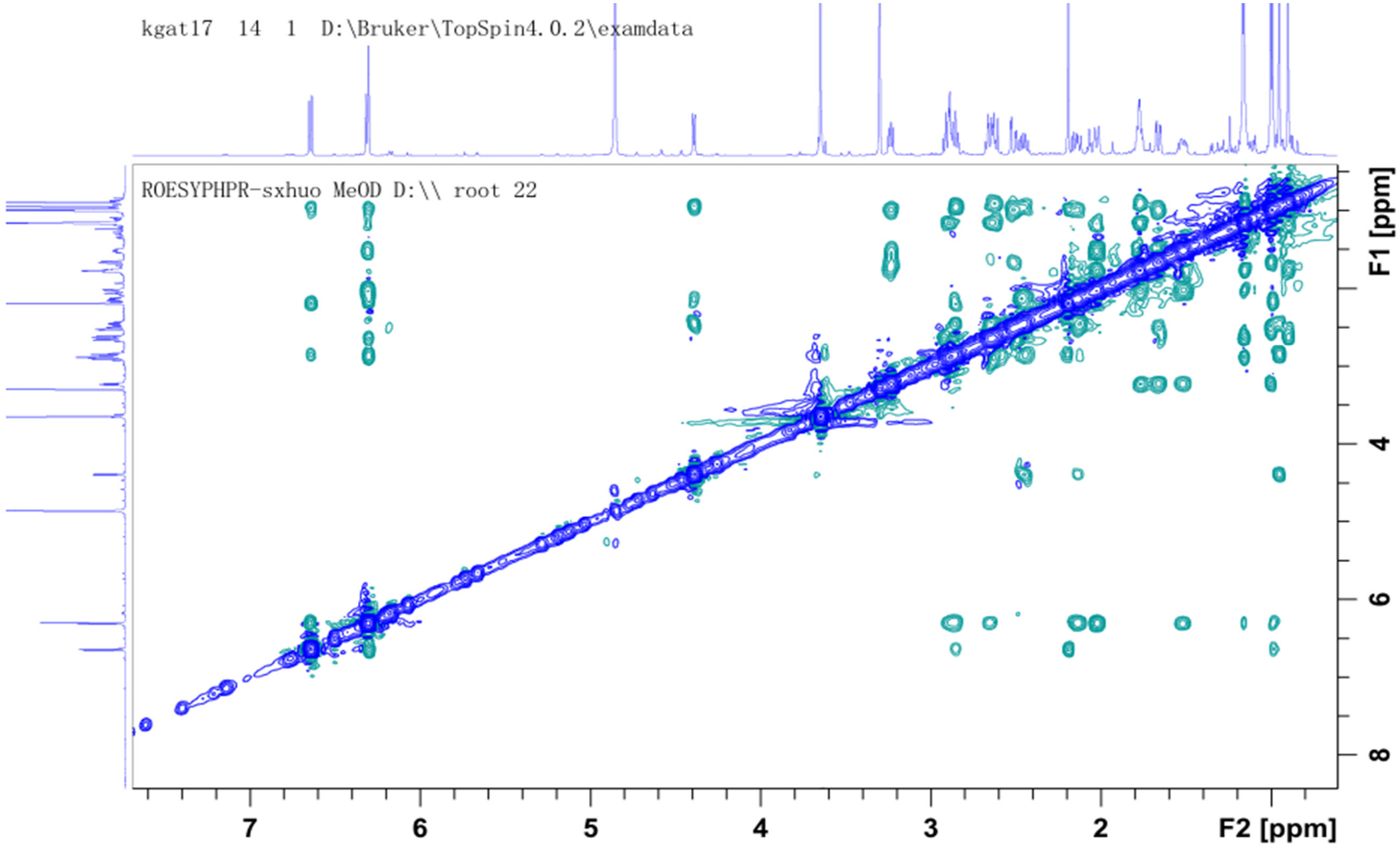

Section S16: HRESIMS spectrum of 7

Figure S52. HRESIMS spectrum of 7.

Qualitative Analysis Report

**Data Filename**	kgat17.d	**Sample Name**	kgat17
**Sample Type**	Sample	**Position**	P1-A6
**Instrument Name**	Instrument 1	**User Name**	
**Acq Method**	s.m	**Acquired Time**	2/25/2021 11:42:54 AM
**IRM Calibration Status**	Success	**DA Method**	Default.m
**Comment**			

Sample Group **Info.**
Acquisition SW 6200 series TOF/6500 series
Version Q-TOF B.05.01 (B5125.2)

User Spectra

Fragmentor Voltage	Collision Energy	Ionization Mode
150	0	ESI

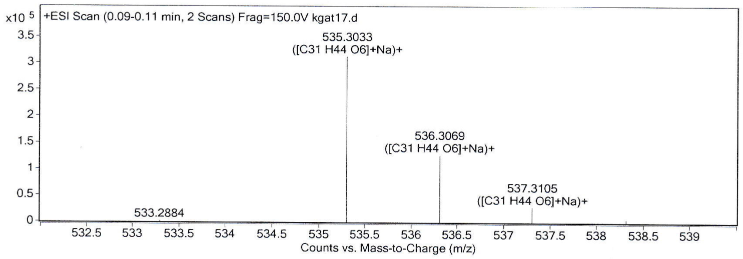

Peak List

m/z	z	Abund	Formula	Ion
102.1274	1	86095.78		
146.0803	1	29545.42		
172.0936	1	51061.72		
513.3209	1	77645.1		
514.3248	1	33235.68		
535.3033	1	312486.22	C31 H44 O6	(M+Na)+
536.3069	1	127098.86	C31 H44 O6	(M+Na)+
1047.6183	1	225229.94		
1048.622	1	183508.3		
1049.625	1	76631.34		

Formula Calculator Element Limits

Element	Min	Max
C	3	60
H	0	120
O	0	30

Formula Calculator Results

Formula	CalculatedMass	CalculatedMz	Mz	Diff. (mDa)	Diff. (ppm)	DBE
C31 H44 O6	512.3138	535.3030	535.3033	-0.30	-0.56	10.0000

--- End Of Report ---

Agilent Technologies Page 1 of 1 Printed at: 2:00 PM on: 2/25/2021

Section S17: 1D and 2D NMR spectra of compound 8

Figure S53. ^{1}H NMR (600 MHz, MeOH-d_4) spectrum of 8.

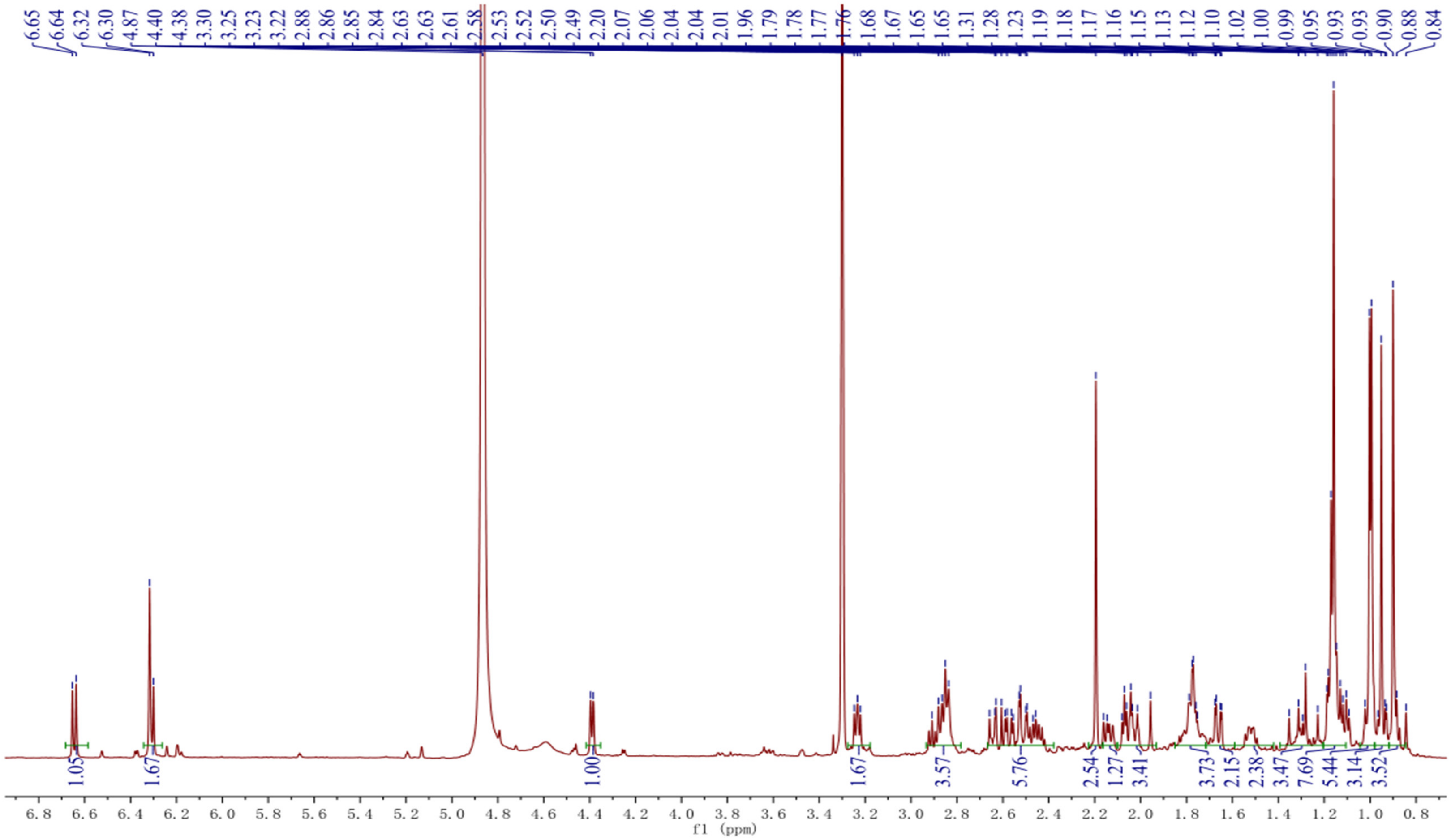

Figure S54. ^{13}C NMR (150 MHz, MeOH-d_4) spectrum of **8.**

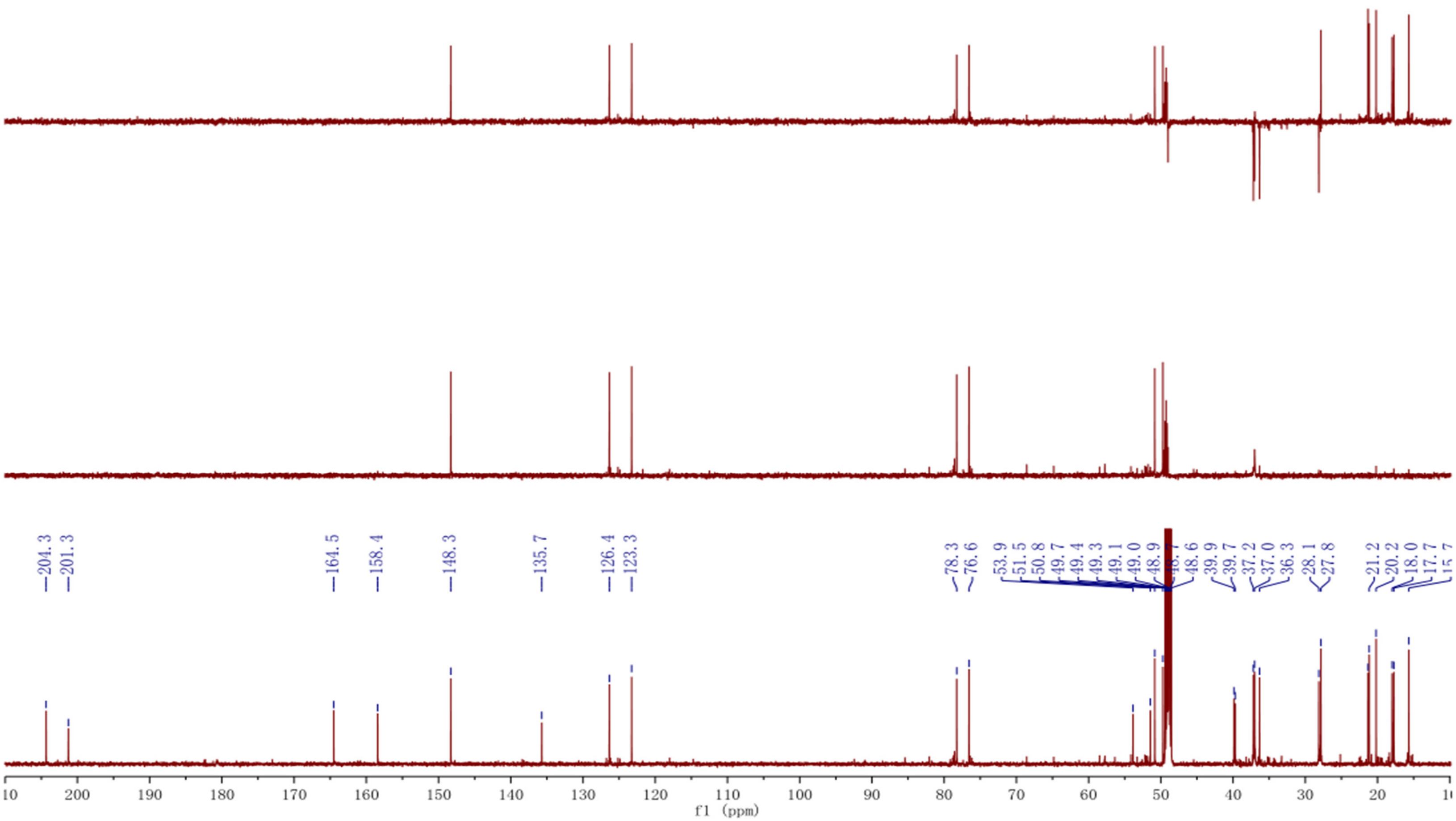

Figure S55. ^{1}H-^{1}H COSY (600 MHz, MeOH-d_4) spectrum of 8.

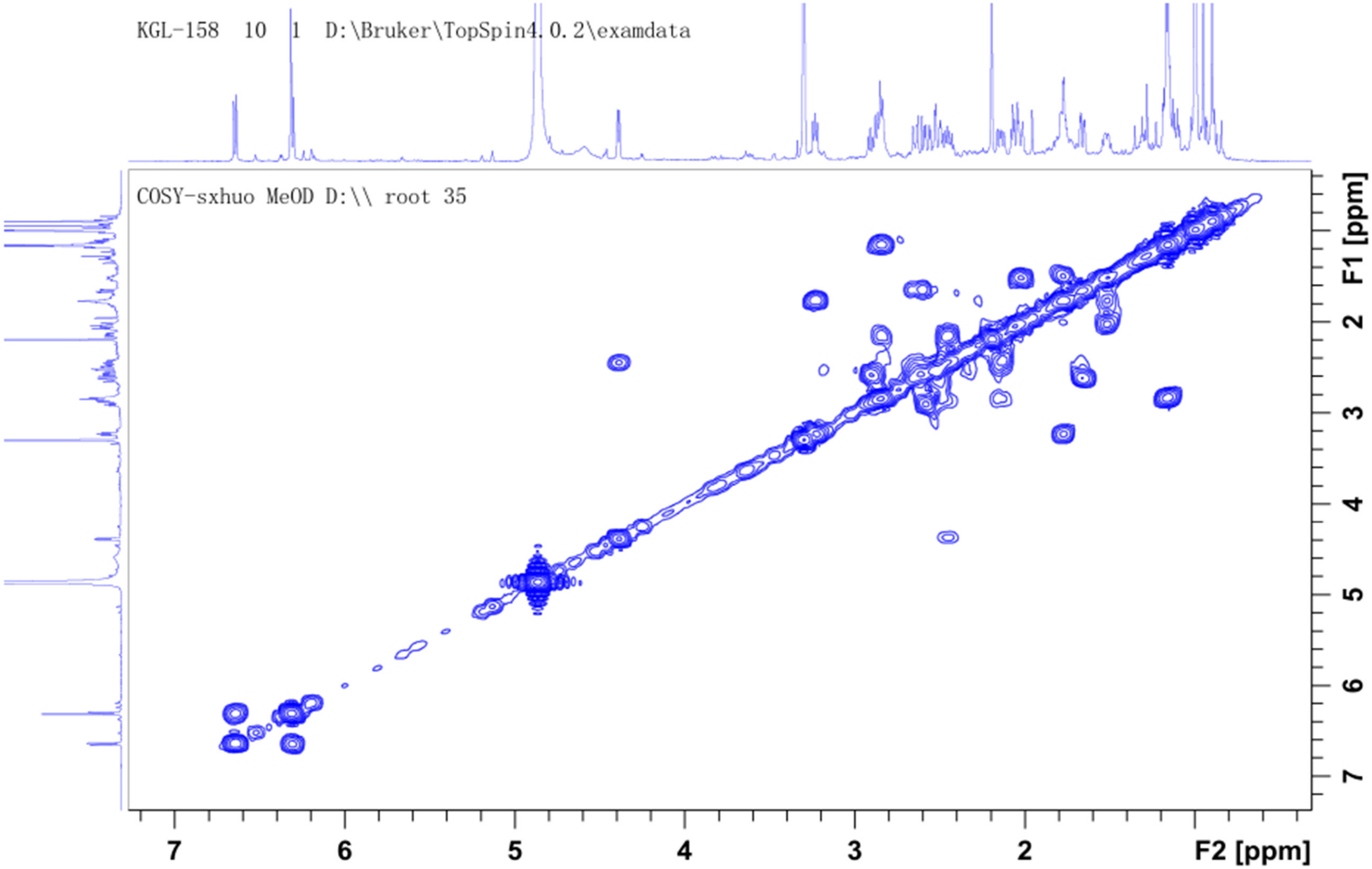

Figure S56. HSQC (600/150 MHz, MeOH-d_4) spectrum of 8.

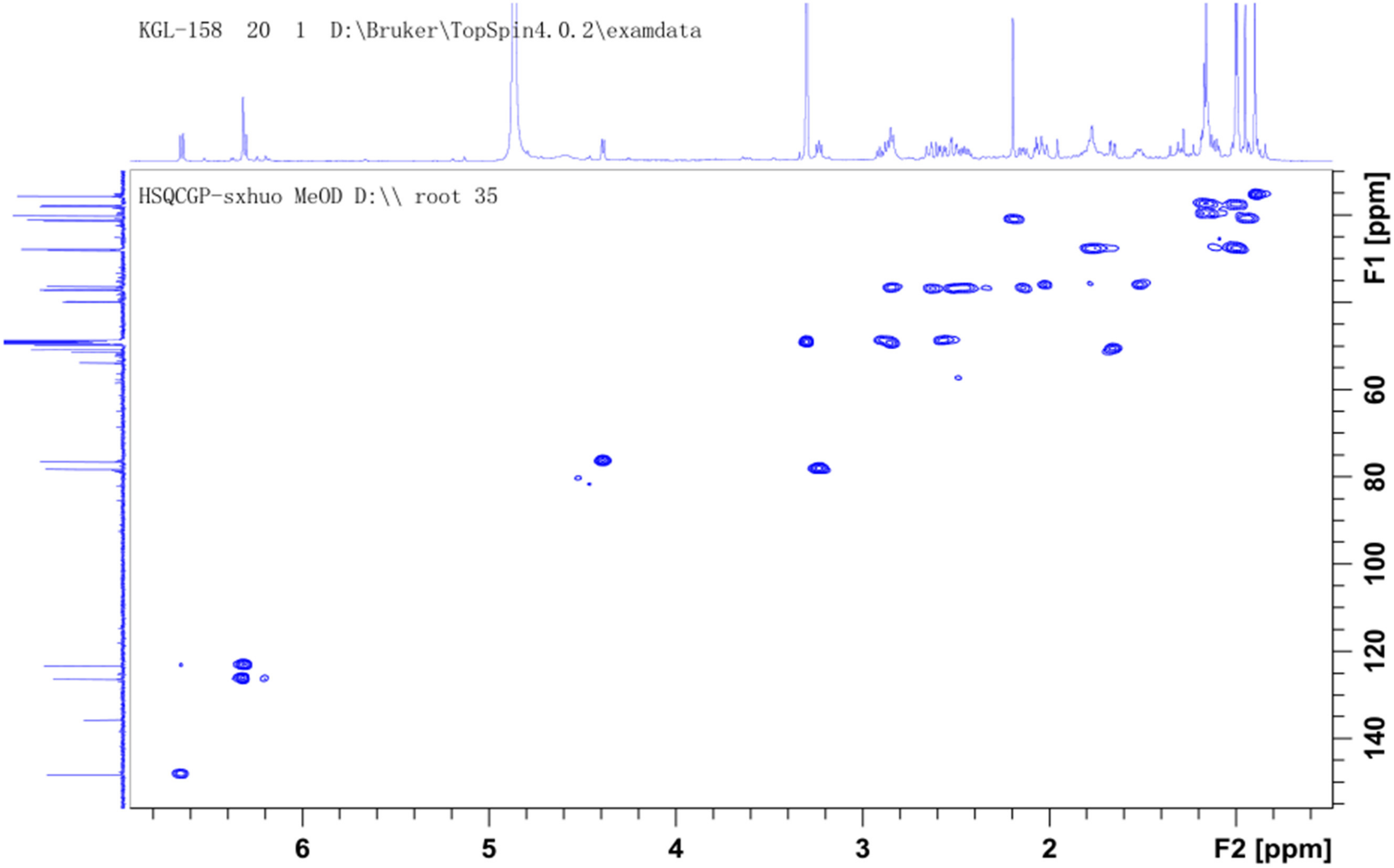

Figure S57. HMBC (600/150 MHz, MeOH-d_4) spectrum of 8.

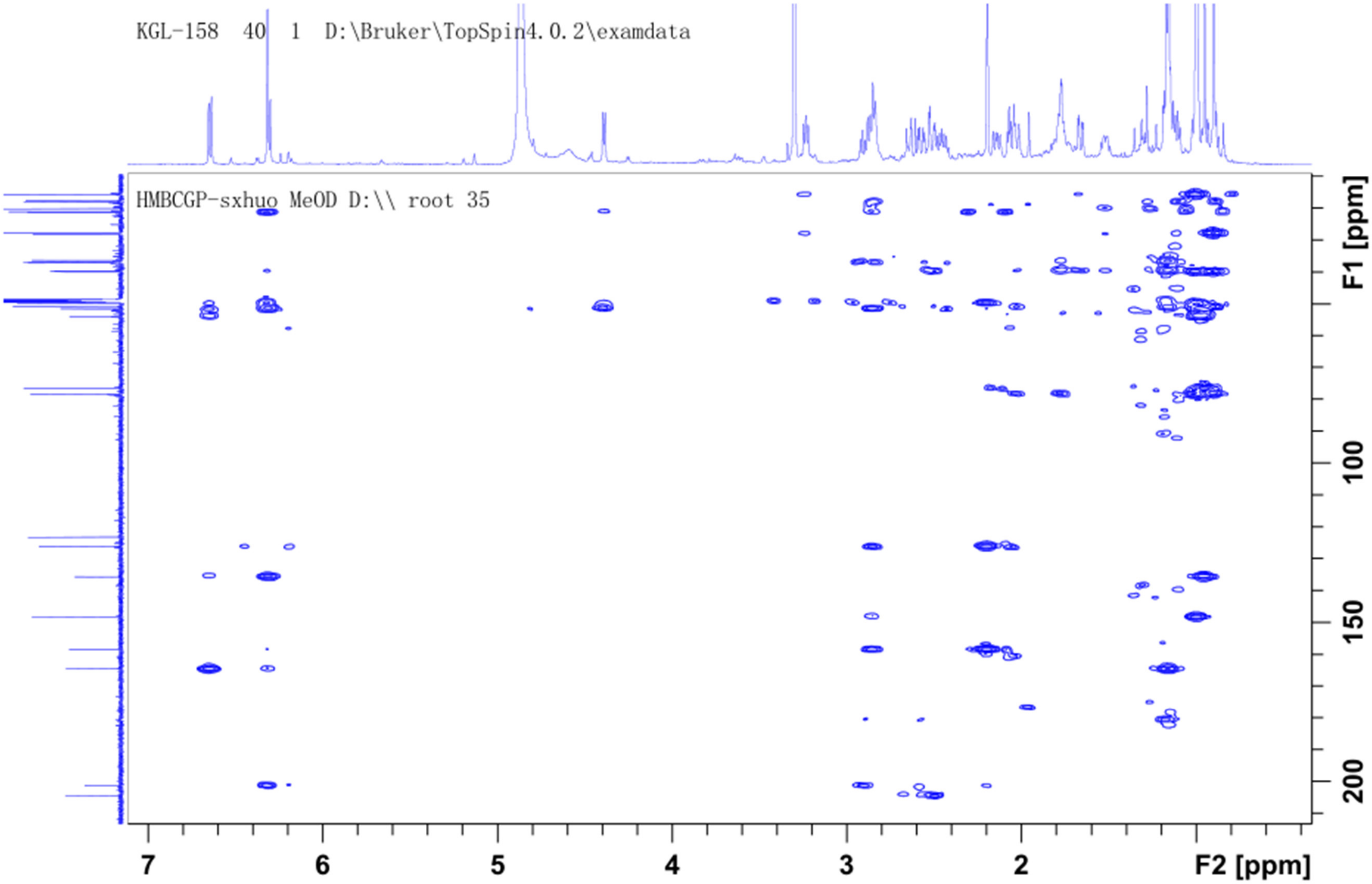

Figure S58. ROESY (600 MHz, MeOH-d_4) spectrum of 8.

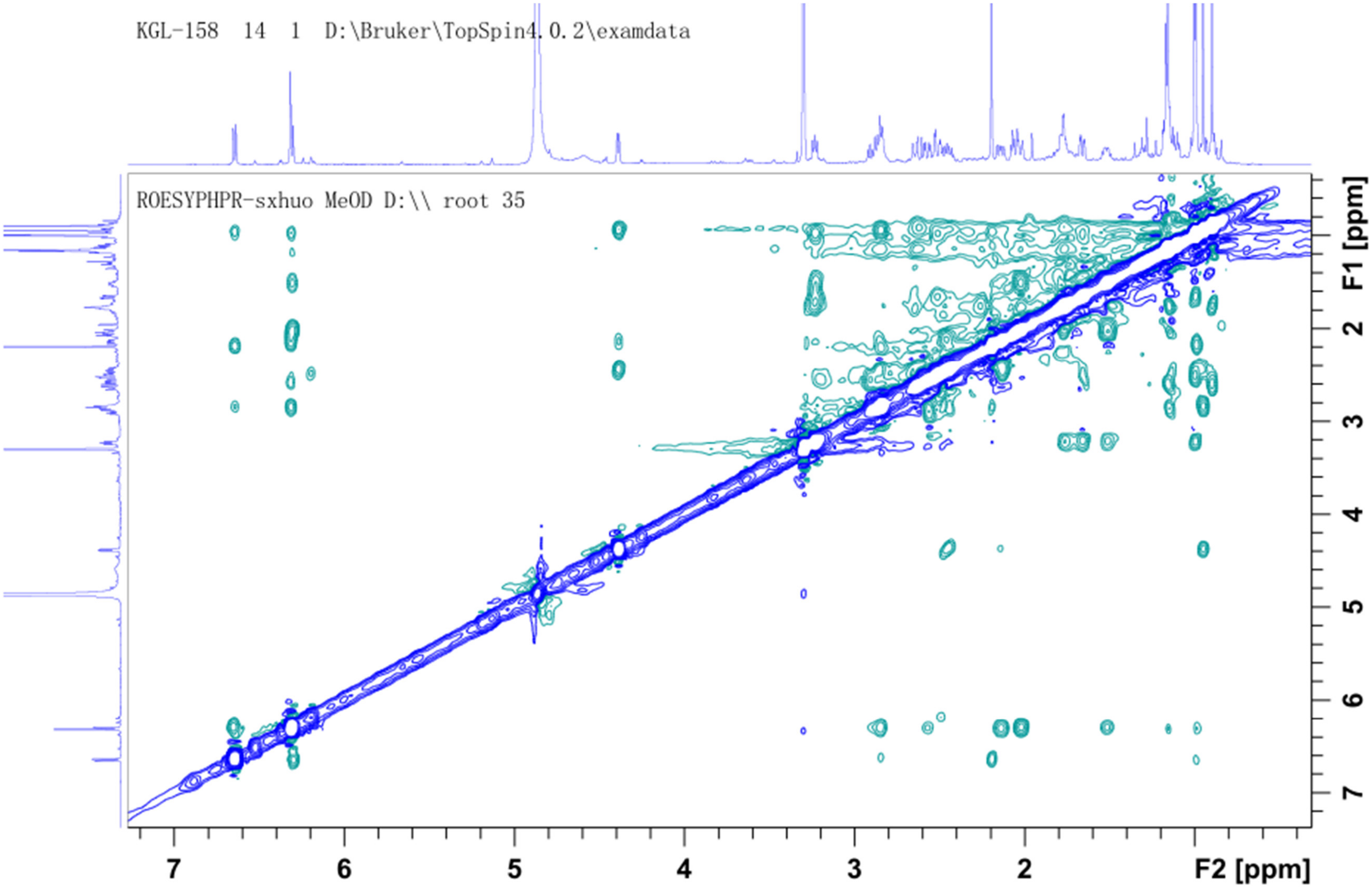

Section S18: HRESIMS spectrum of 8

Figure S59. HRESIMS spectrum of 8.

Qualitative Analysis Report

**Data Filename**	kgl-158.d	**Sample Name**	kgl-158
**Sample Type**	Sample	**Position**	P1-A2
**Instrument Name**	Instrument 1	**User Name**	
**Acq Method**	s-.m	**Acquired Time**	10/12/2020 11:08:38 AM
**IRM Calibration Status**	Success	**DA Method**	ZRR3.m
**Comment**			

Sample Group **Info.**

Acquisition SW Version 6200 series TOF/6500 series Q-TOF B.05.01 (B5125.2)

User Spectra

Fragmentor Voltage	Collision Energy	Ionization Mode
175	0	ESI

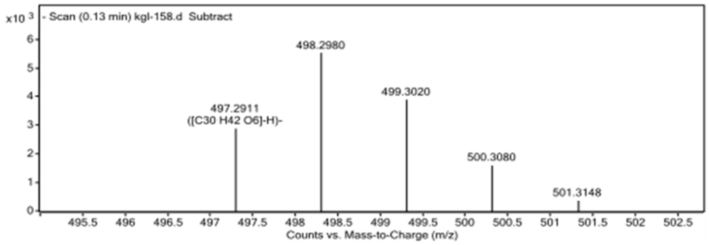

Peak List

m/z	z	Abund	Formula	Ion
497.2911		2925.91	C30 H42 O6	(M-H)-
498.298		5542.1		
499.302	1	3925.09		
500.308	1	1642.42		
512.2784		921.76		
513.2855		1117.4		
514.292	1	1240.95		
515.3006	1	692.01		
566.2904	1	797.03		
1033.9881		1762.93		

Formula Calculator Element Limits

Element	Min	Max
C	3	60
H	0	120
O	0	30

Formula Calculator Results

Formula	CalculatedMass	CalculatedMz	Mz	Diff. (mDa)	Diff. (ppm)	DBE
C30 H42 O6	498.2981	497.2909	497.2911	-0.20	-0.40	10.0000

--- End Of Report ---

Agilent Technologies   Page 1 of 1   Printed at: 11:10 AM on: 10/12/2020

Section S19: 1D and 2D NMR spectra of compound 9

Figure S60. ^1H **NMR (600 MHz,** $CDCl_3$**) spectrum of 9.**

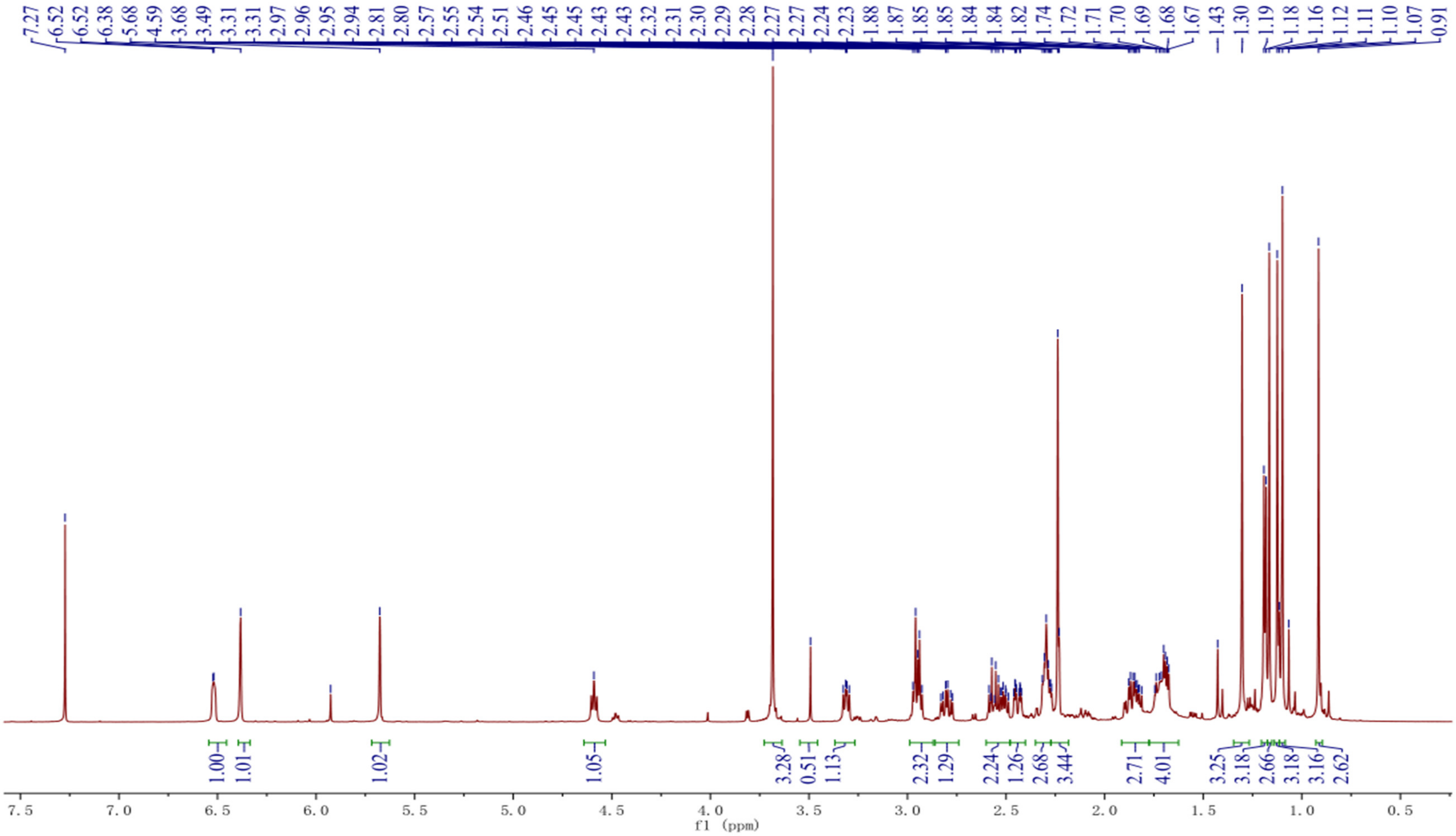

Figure S61. ^{13}**C NMR (150 MHz,** $CDCl_3$**) spectrum of 9.**

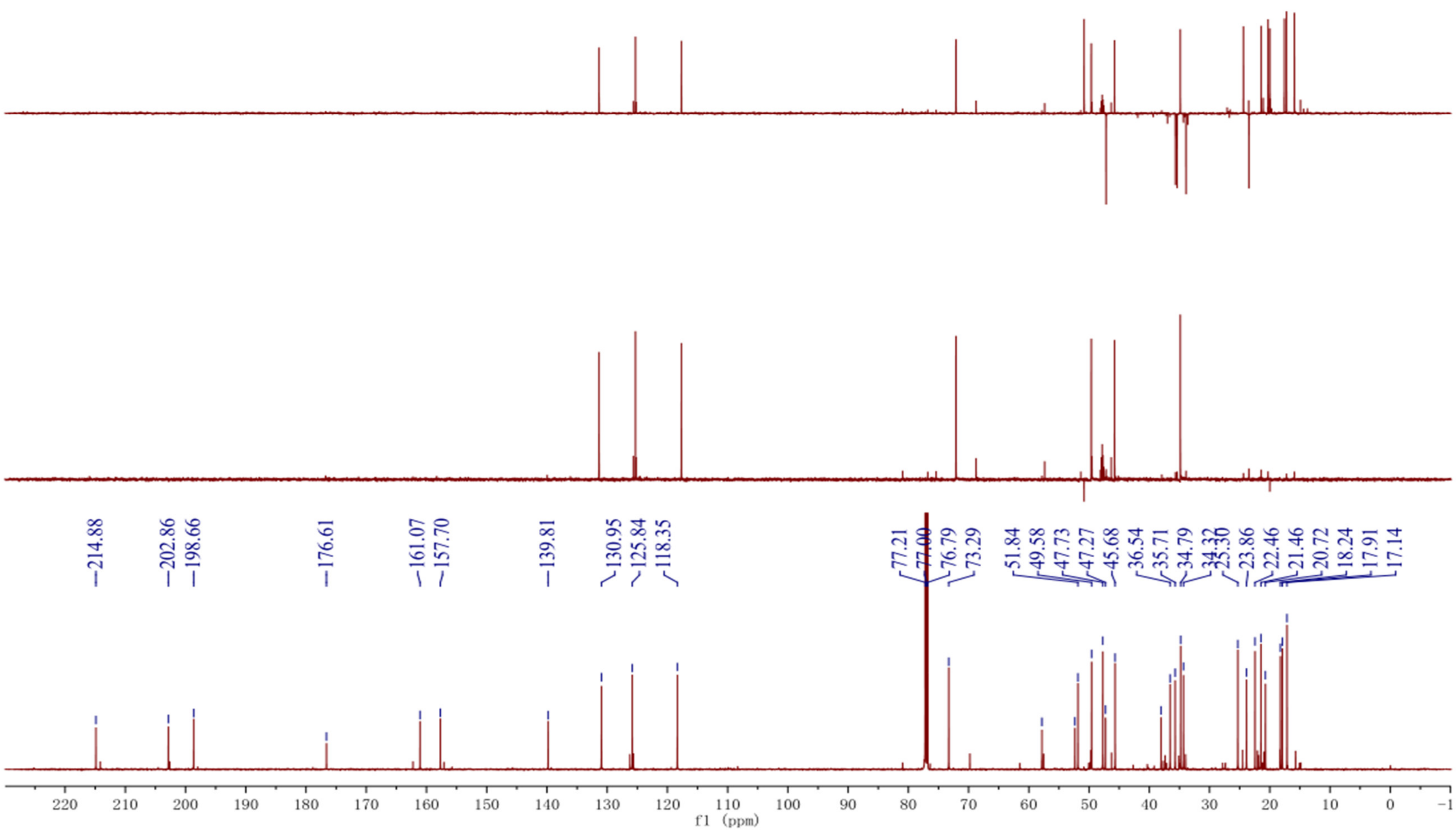

Figure S62. ^1H-^1H **COSY (600 MHz, $CDCl_3$) spectrum of 9.**

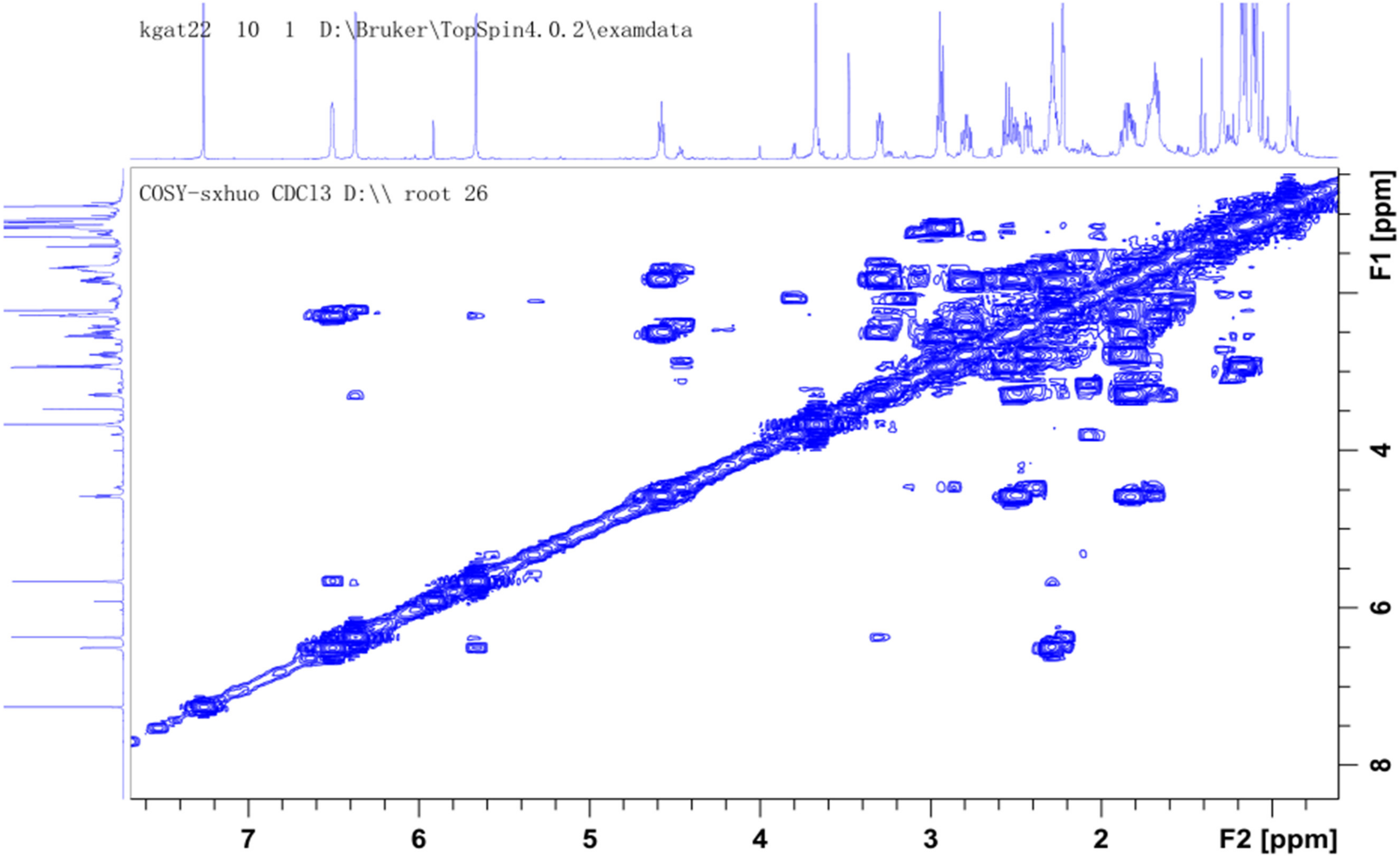

Figure S63. HSQC (600/150 MHz, $CDCl_3$) spectrum of 9.

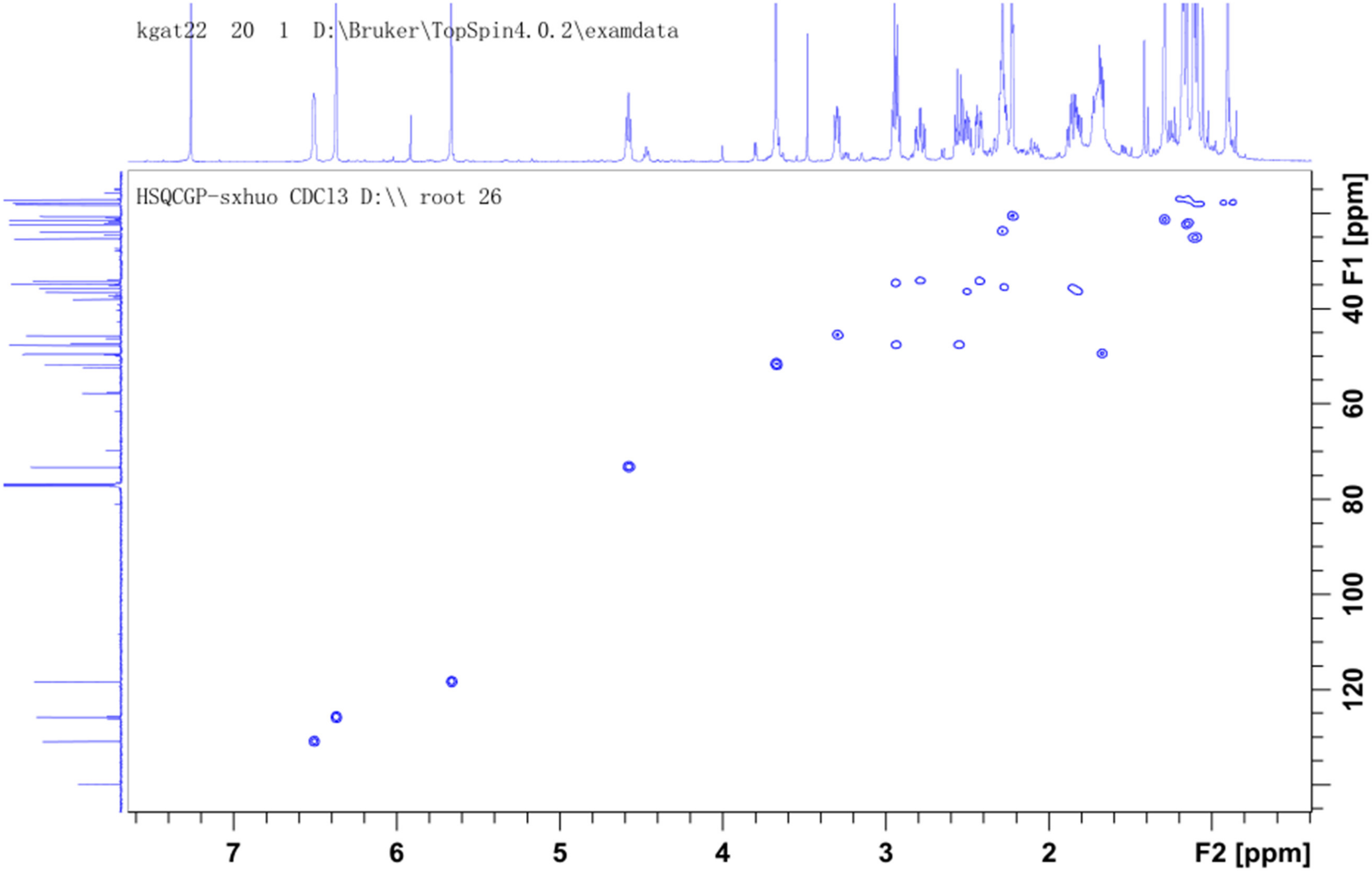

Figure S64. HMBC (600/150 MHz, $CDCl_3$) spectrum of 9.

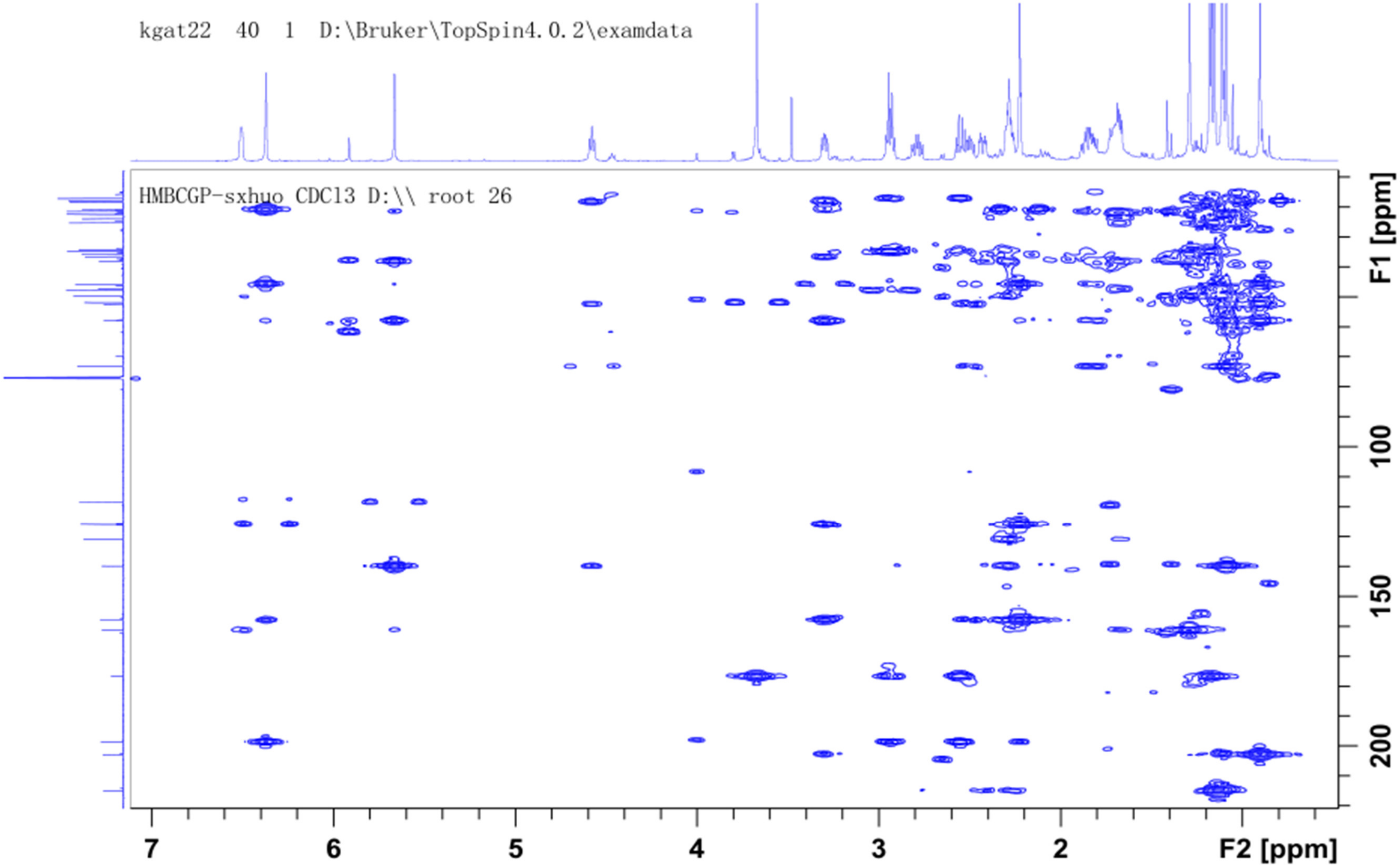

Figure S65. ROESY (600/150 MHz, $CDCl_3$) spectrum of 9.

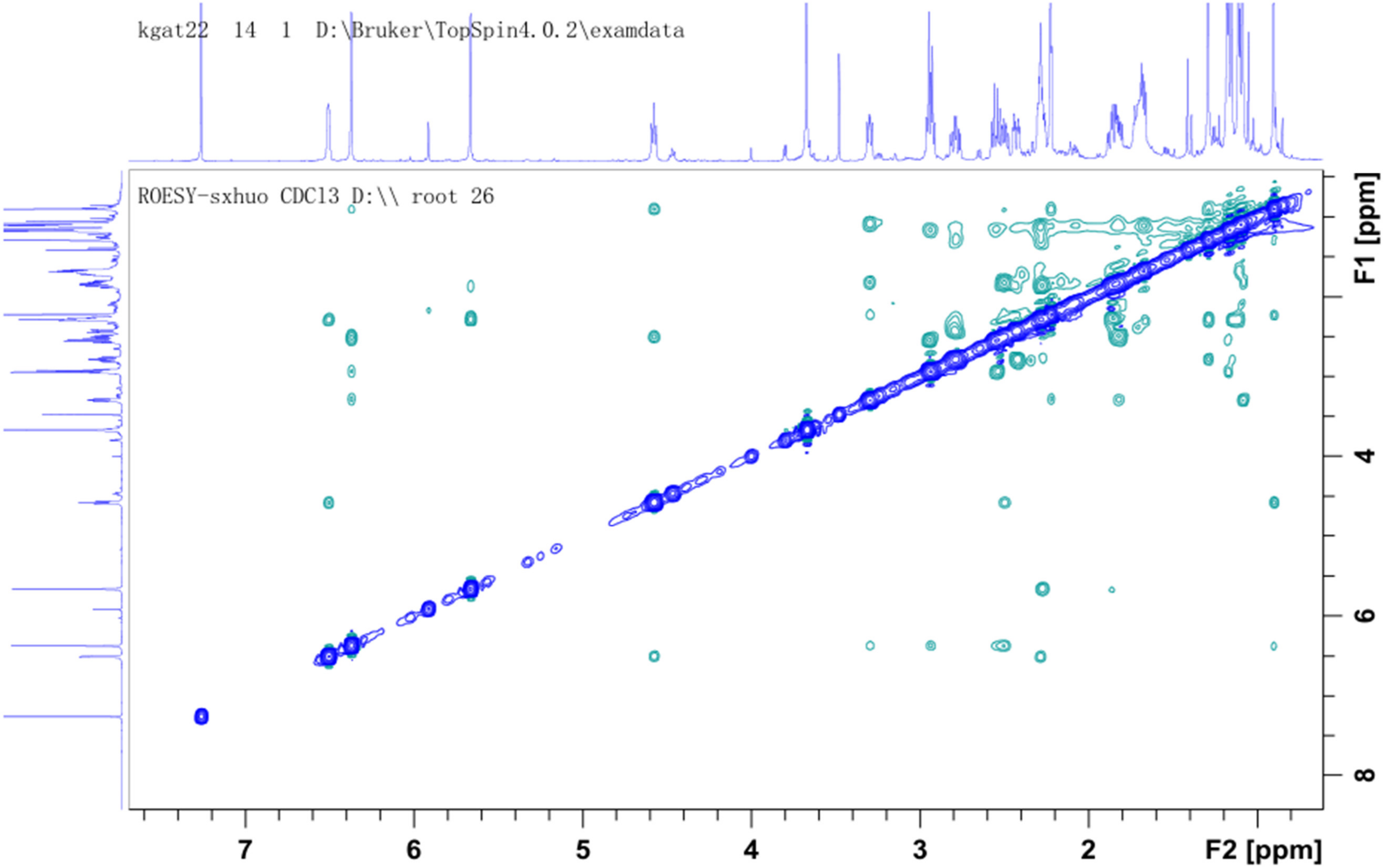

Section S20: HRESIMS spectrum of 9

Figure S66. HRESIMS spectrum of 9.

Qualitative Analysis Report

**Data Filename**	kgat22.d	**Sample Name**	kgat22
**Sample Type**	Sample	**Position**	P1-A9
**Instrument Name**	Instrument 1	**User Name**	
**Acq Method**	s.m	**Acquired Time**	2/25/2021 11:46:27 AM
**IRM Calibration Status**	Success	**DA Method**	Default.m
**Comment**			

Sample Group **Info.**
Acquisition SW Version 6200 series TOF/6500 series Q-TOF B.05.01 (B5125.2)

User Spectra

Fragmentor Voltage	Collision Energy	Ionization Mode
150	0	ESI

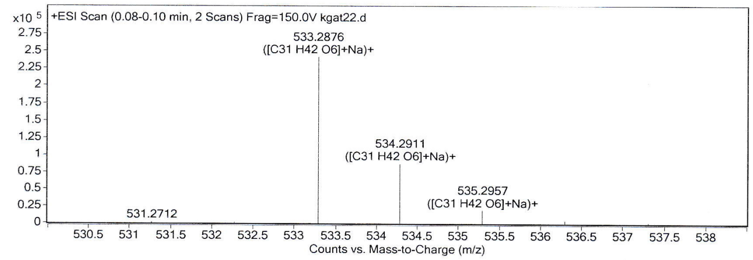

Peak List

m/z	z	Abund	Formula	Ion
102.1274	1	24207.48		
172.0936	1	56103.34		
511.3051	1	46827.01		
533.2876	1	242101.48	C31 H42 O6	(M+Na)+
534.2911	1	87653.3	C31 H42 O6	(M+Na)+
549.2734	1	36785.6		
1043.5863	1	125190.95		
1044.5899	1	93311.35		
1045.5928	1	41897.05		
1059.5807	1	31313.65		

Formula Calculator Element Limits

Element	Min	Max
C	3	60
H	0	120
O	0	30

Formula Calculator Results

Formula	CalculatedMass	CalculatedMz	Mz	Diff. (mDa)	Diff. (ppm)	DBE
C31 H42 O6	510.2981	533.2874	533.2876	-0.20	-0.38	11.0000

--- End Of Report ---

Agilent Technologies Page 1 of 1 Printed at: 2:02 PM on: 2/25/2021

Section S21: 1D and 2D NMR spectra of compound 10

Figure S67. ^{1}H NMR (600 MHz, MeOH-d_4) spectrum of 10.

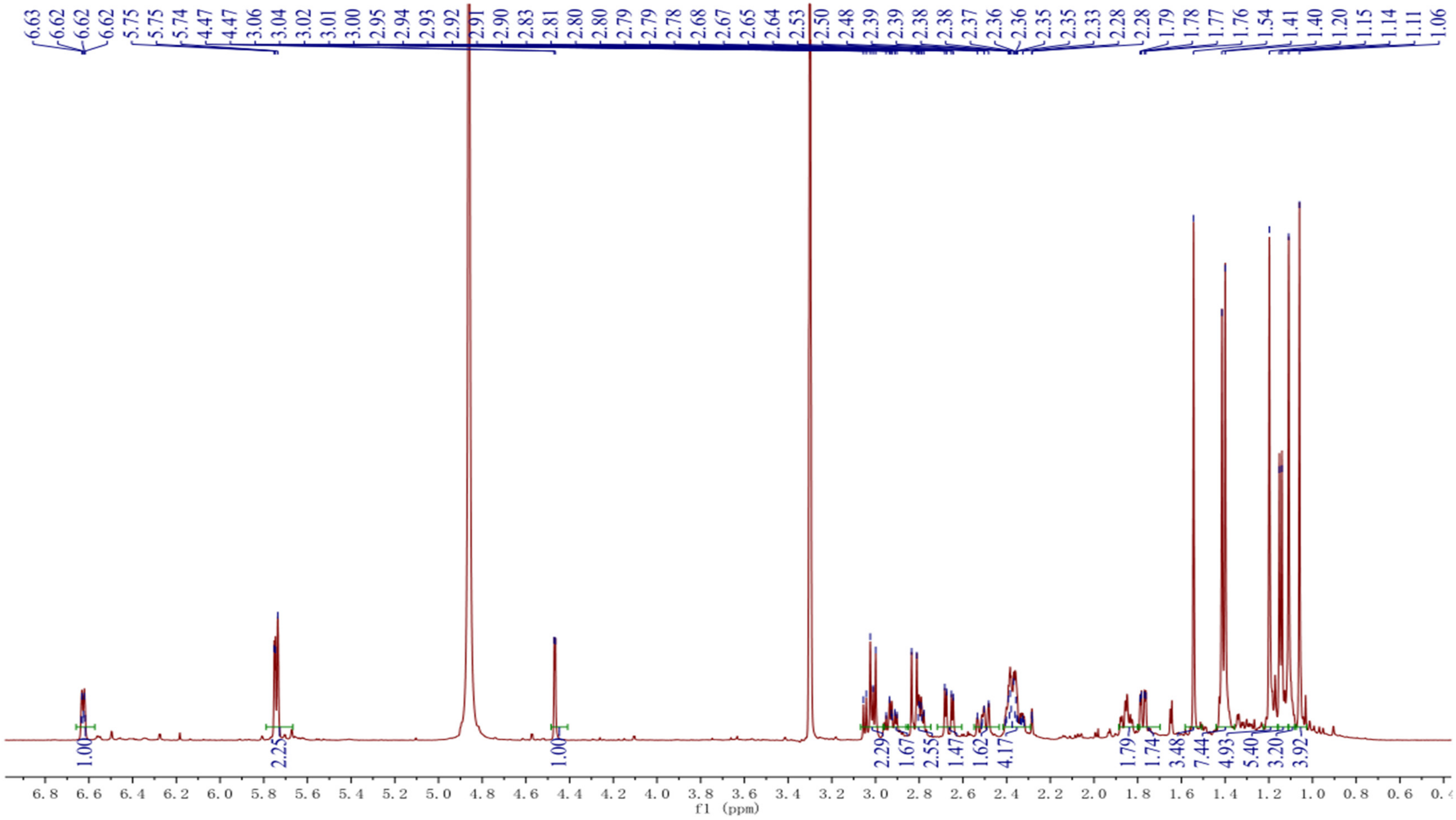

Figure S68. ^{13}**C NMR (150 MHz, MeOH-*d*$_4$) spectrum of 10.**

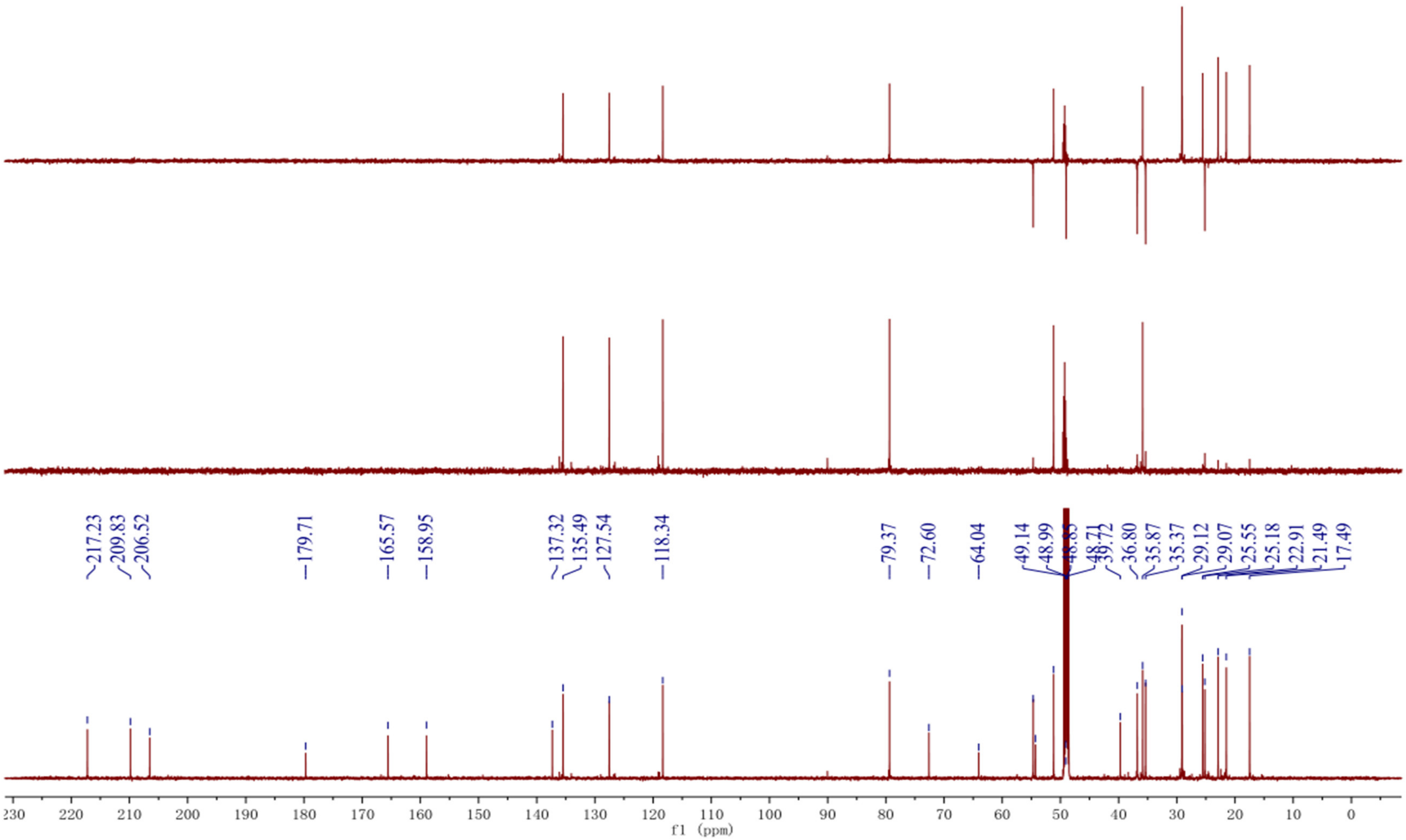

Figure S69. **^{1}H-^{1}H COSY (600 MHz, MeOH-d_4) spectrum of 10.**

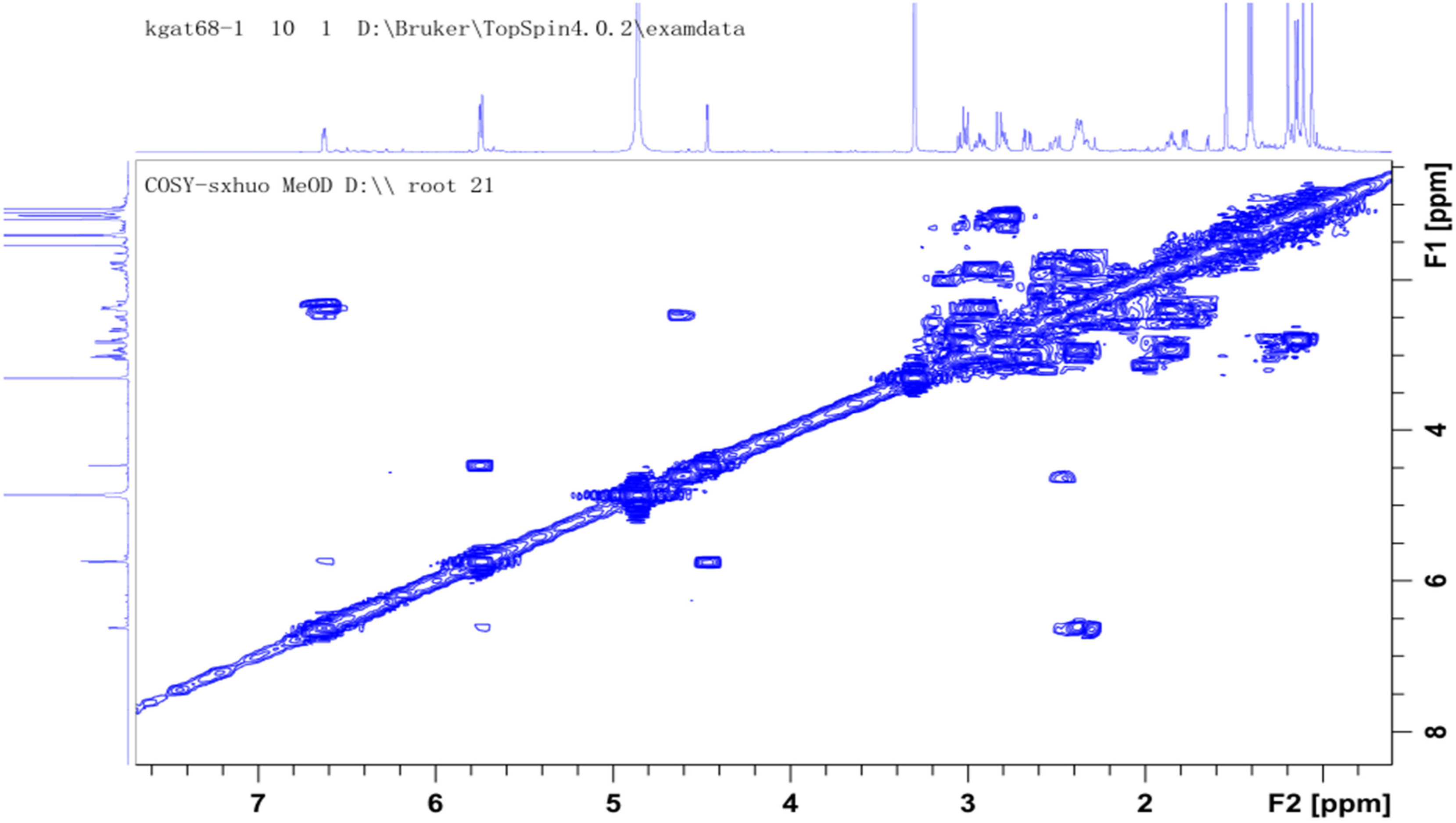

Figure S70. HSQC (600/150 MHz, MeOH-d_4) spectrum of 10.

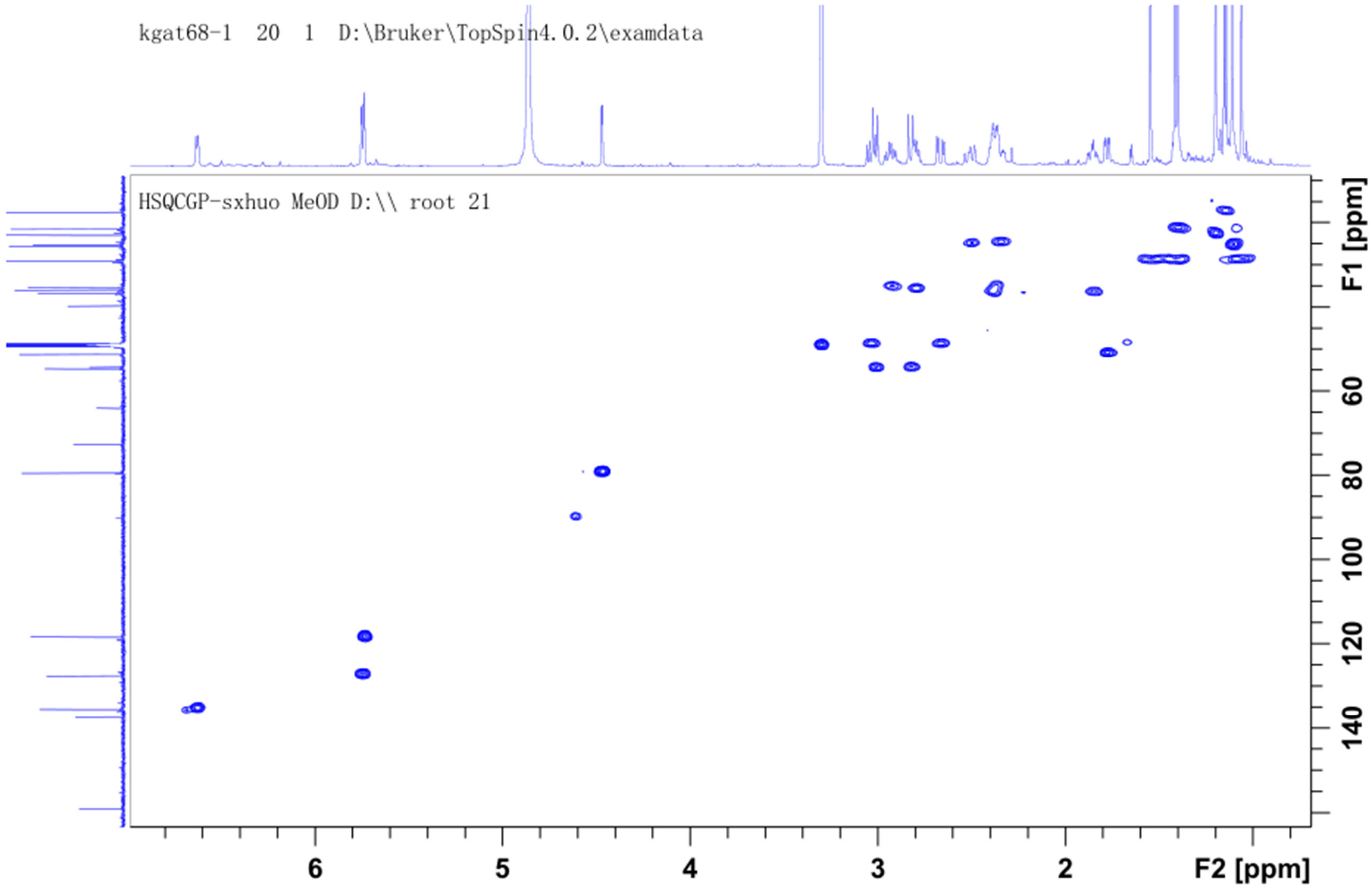

Figure S71. HMBC (600/150 MHz, MeOH-d_4) spectrum of 10.

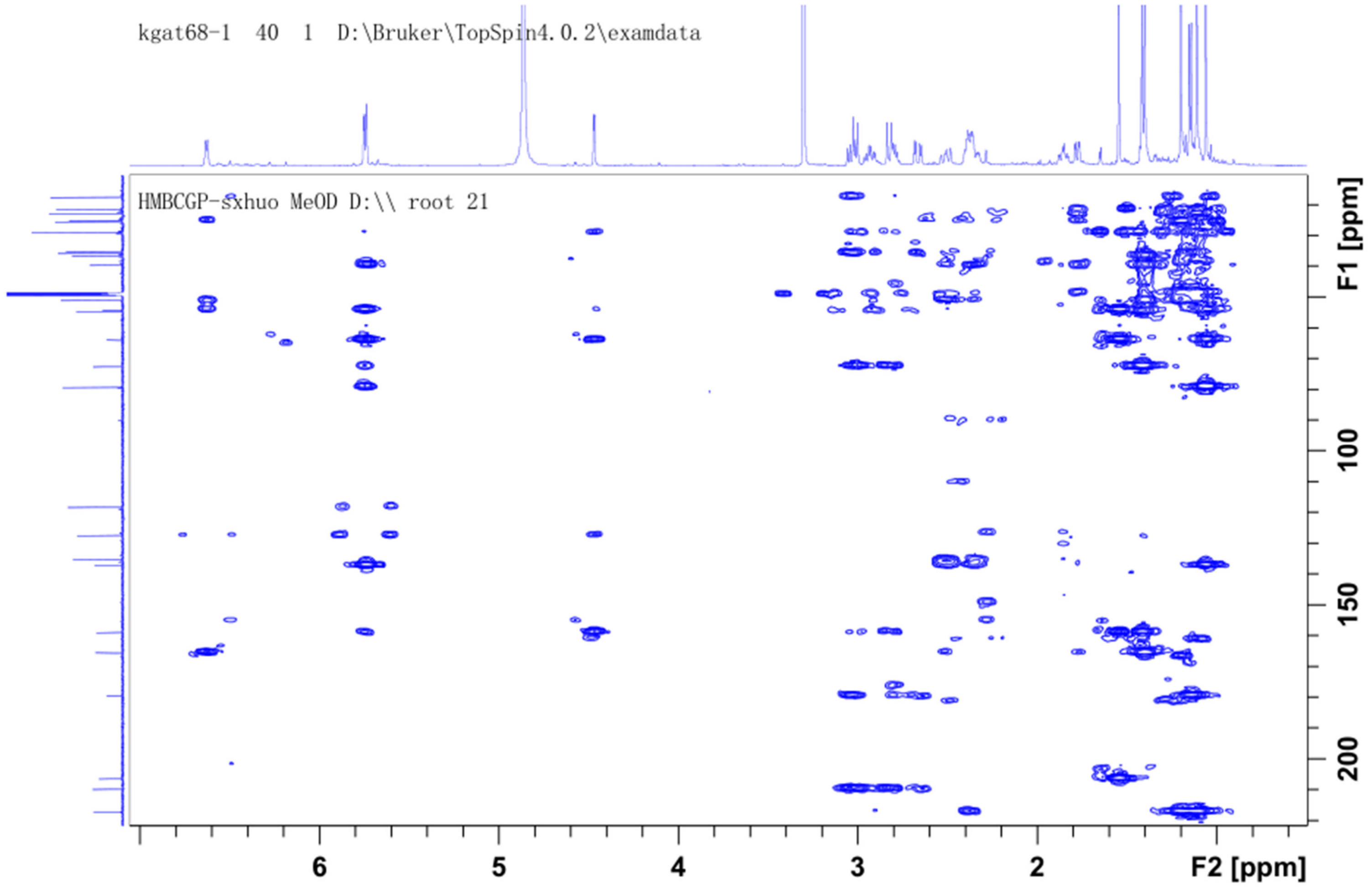

Figure S72. ROESY (600/150 MHz, MeOH-d_4) spectrum of 10.

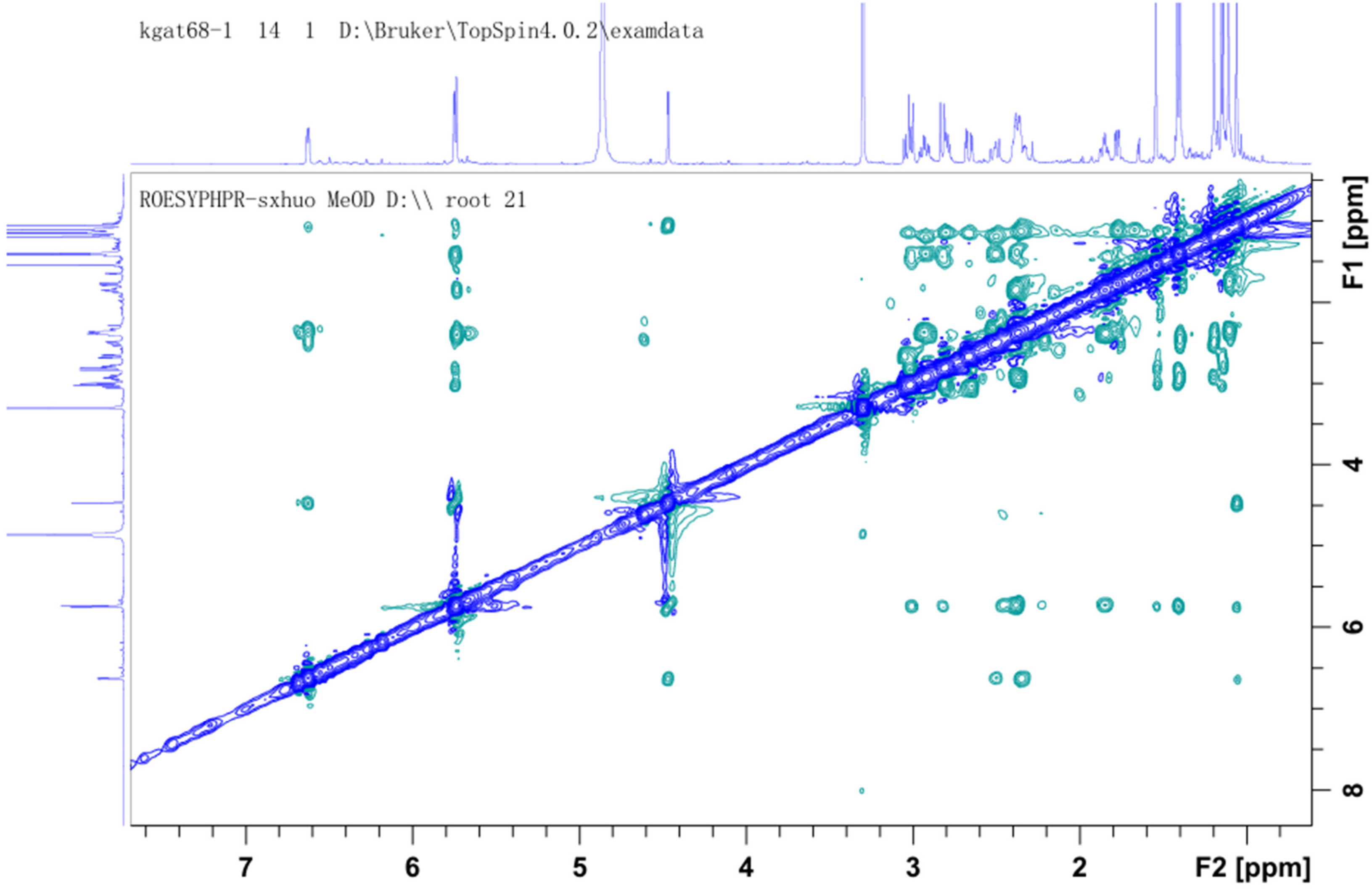

Section S22: HRESIMS spectrum of 10

Figure S73. HRESIMS spectrum of 10.

Qualitative Analysis Report

**Data Filename**	kgat68-1.d	**Sample Name**	kgat68-1
**Sample Type**	Sample	**Position**	P1-A6
**Instrument Name**	Instrument 1	**User Name**	
**Acq Method**	s-.m	**Acquired Time**	7/9/2021 3:17:33 PM
**IRM Calibration Status**	Success	**DA Method**	Default.m
**Comment**			

**Sample Group**		**Info.**
**Acquisition SW Version**	6200 series TOF/6500 series Q-TOF B.05.01 (B5125.2)	

User Spectra

Fragmentor Voltage	Collision Energy	Ionization Mode
180	0	ESI

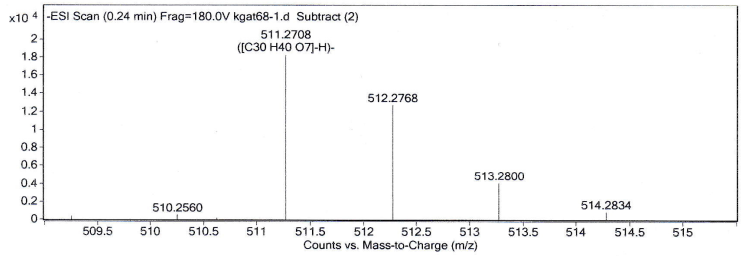

Peak List

m/z	z	Abund	Formula	Ion
129.0554		2320		
493.2601		1402.9		
494.2642	1	1299.74		
511.2708		18227.04	C30 H40 O7	(M-H)-
512.2768	1	12667.26		
513.28	1	4049.29		
527.2673		3307.64		
528.2705	1	2243.17		
528.3024	1	1029.55		
529.2735	1	1051.21		
579.2585	1	2825.05		
580.2648	1	1943.69		

Formula Calculator Element Limits

Element	Min	Max
C	3	60
H	0	120
O	0	30

Formula Calculator Results

Formula	CalculatedMass	CalculatedMz	Mz	Diff. (mDa)	Diff. (ppm)	DBE
C30 H40 O7	512.2774	511.2701	511.2708	-0.70	-1.37	11.0000

--- End Of Report ---

Section S23: 1D and 2D NMR spectra of compound 11

Figure S74. ^{1}**H NMR (600 MHz, MeOH-**d_4**) spectrum of 11.**

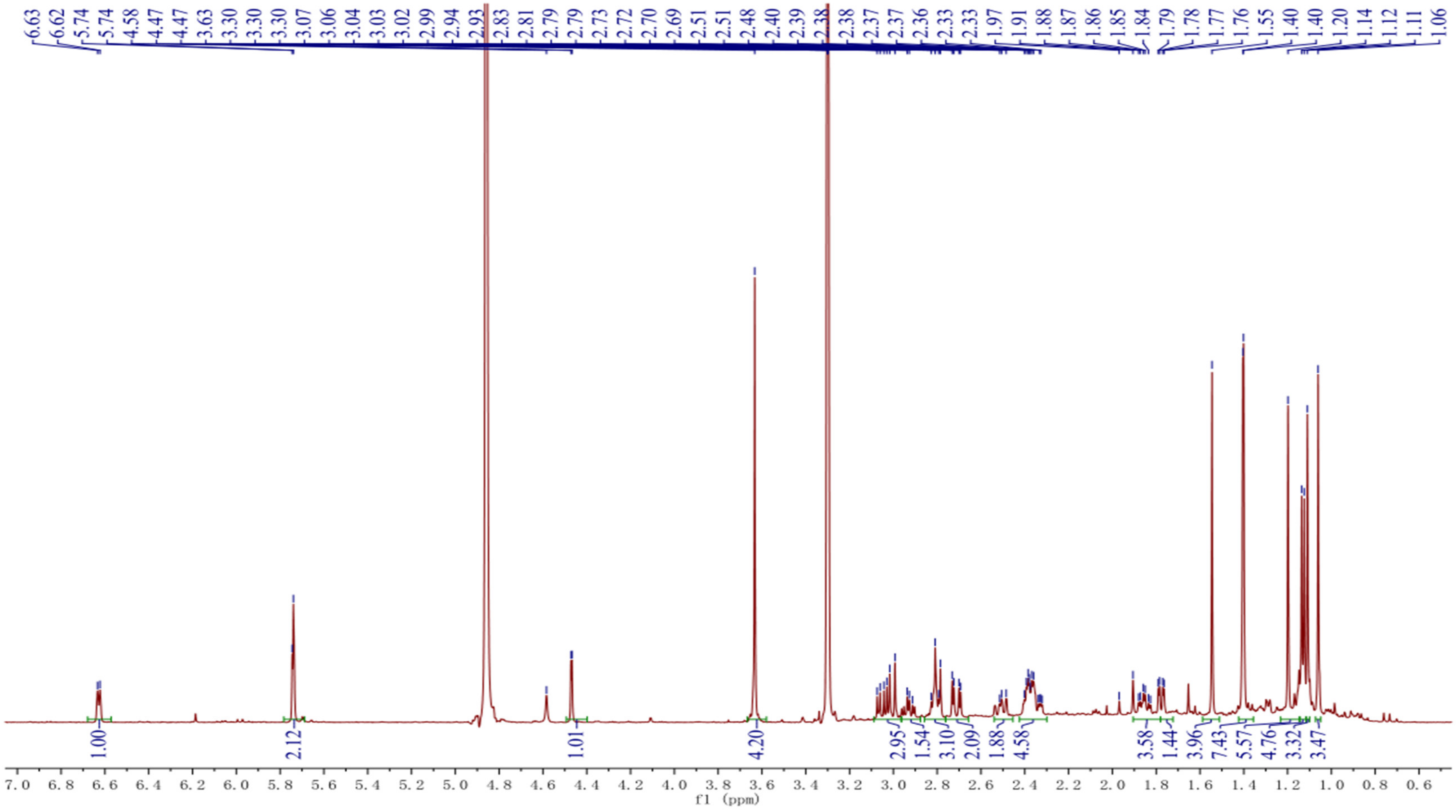

Figure S75. ^{13}C NMR (150 MHz, MeOH-d_4) spectrum of 11.

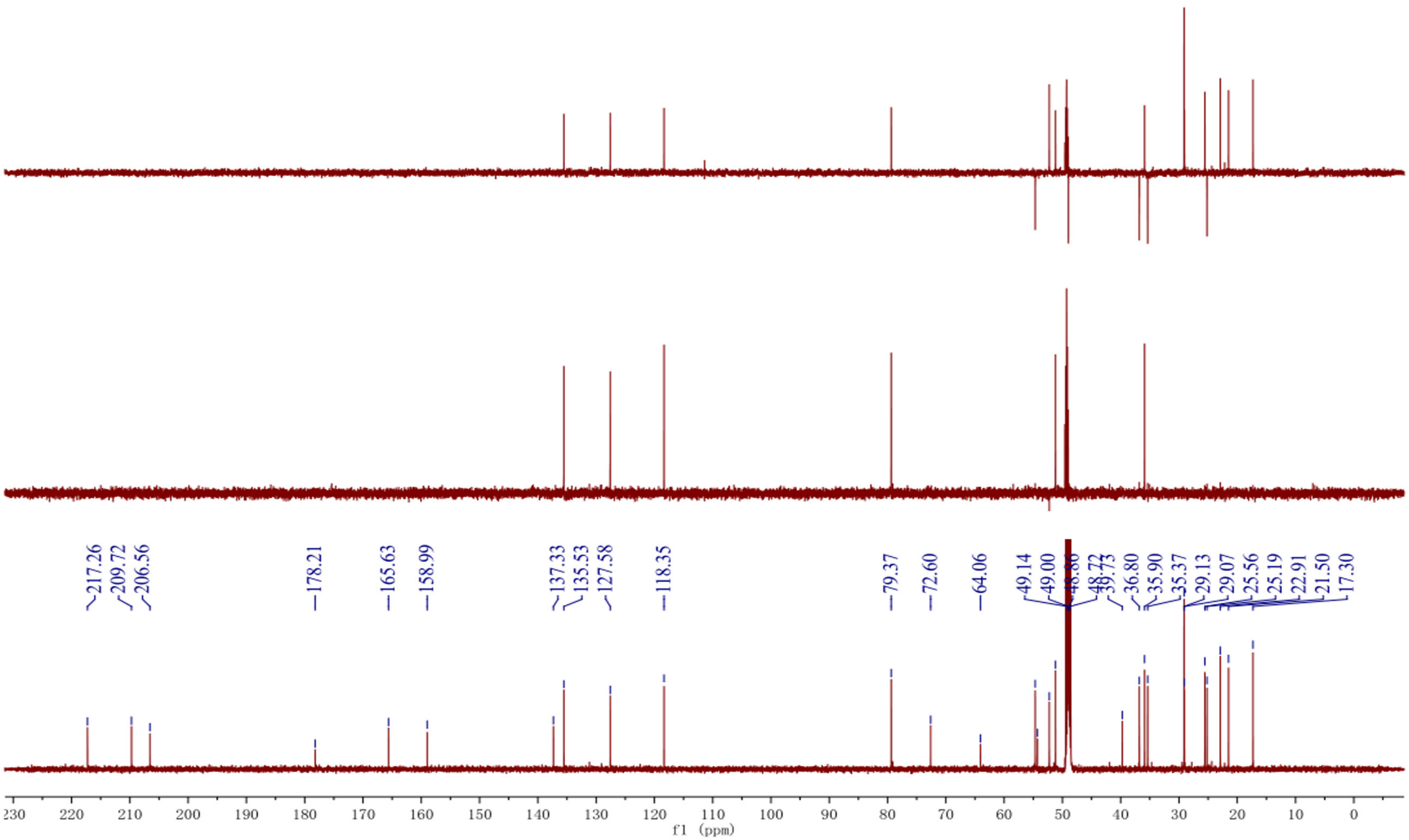

Figure S76. ^{1}H-^{1}H **COSY (600 MHz, MeOH-*d*$_4$) spectrum of 11.**

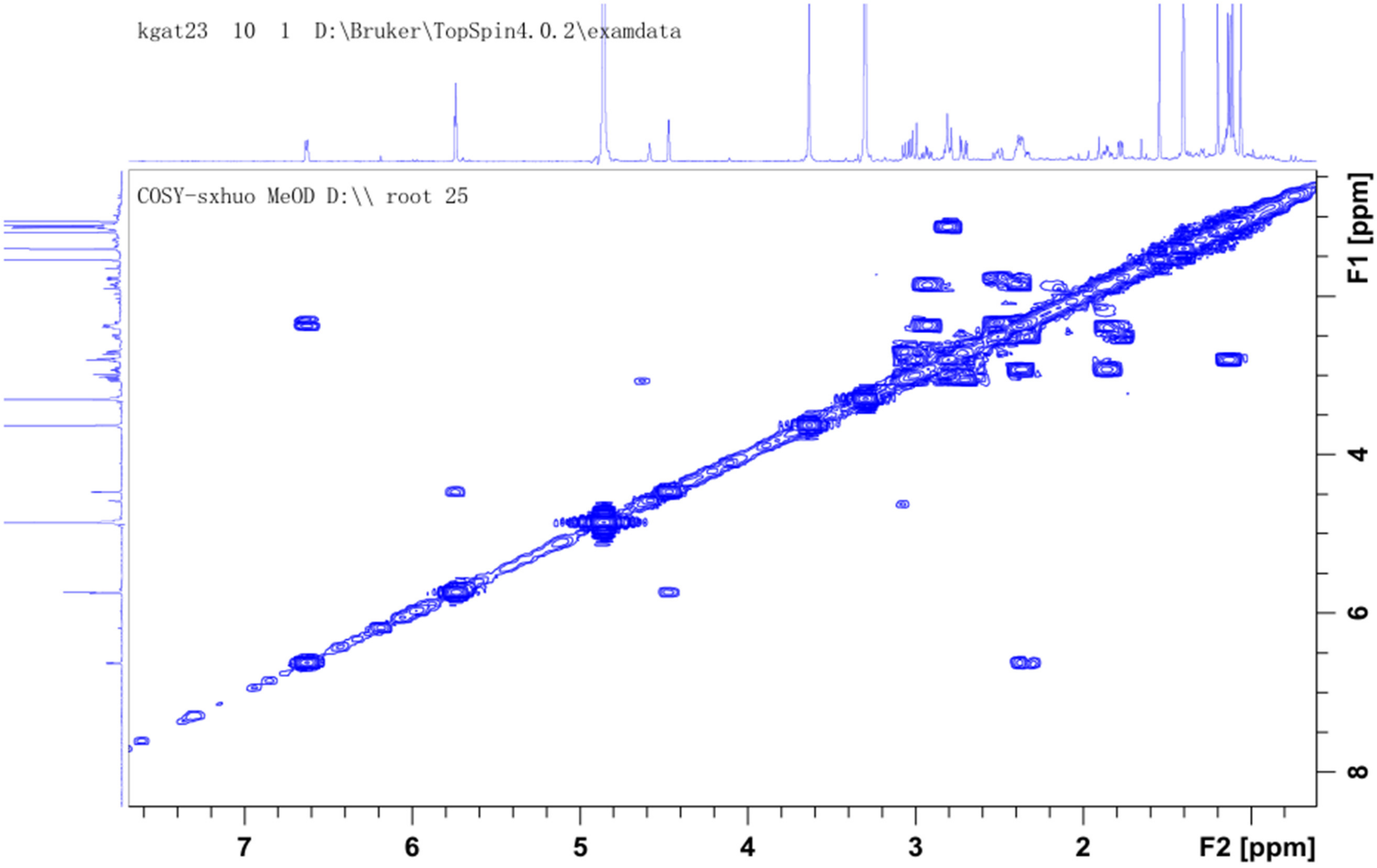

Figure S77. HSQC (600/150 MHz, MeOH-d_4) spectrum of 11.

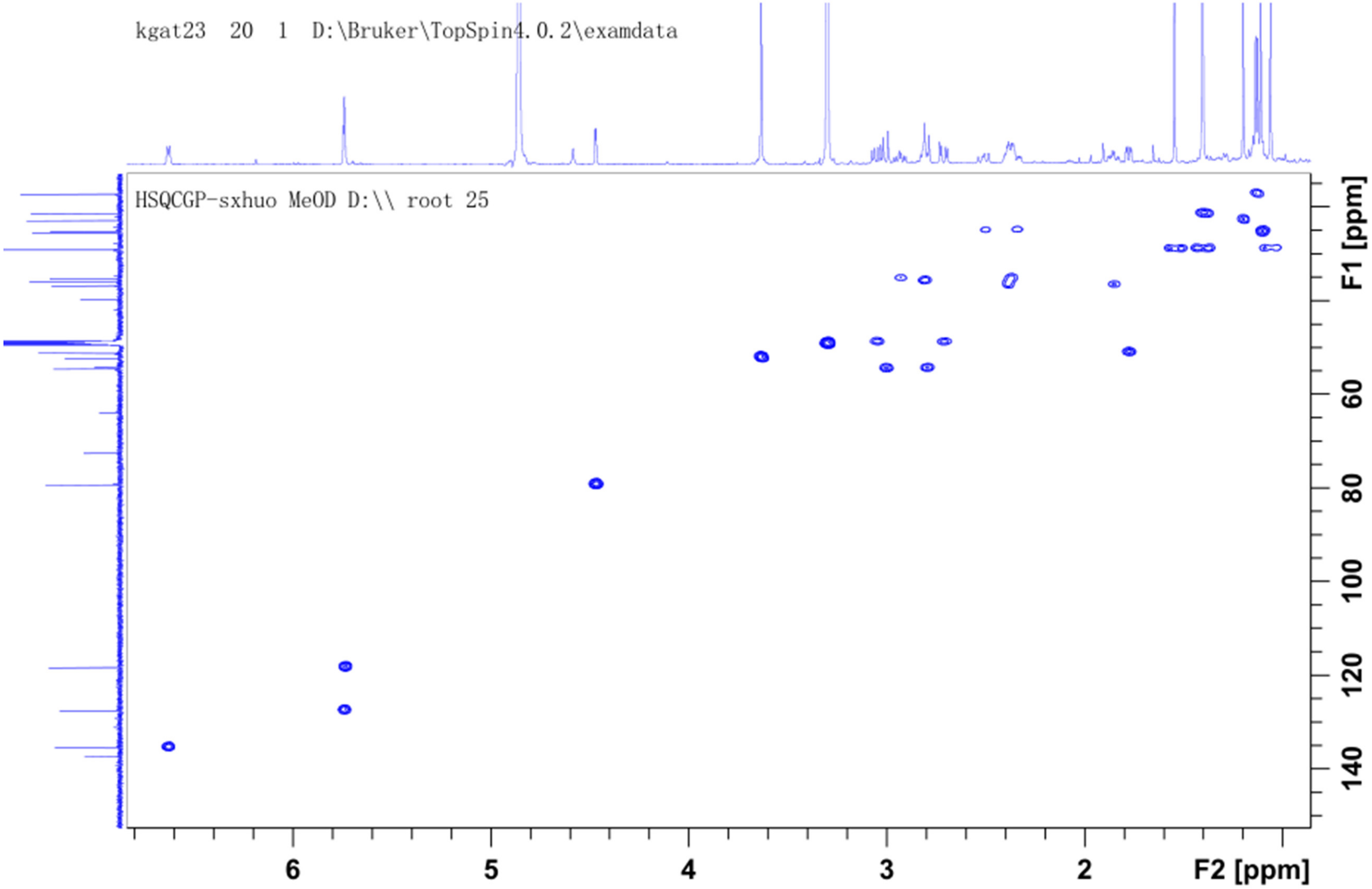

Figure S78. HMBC (600/150 MHz, MeOH-d_4) spectrum of 11.

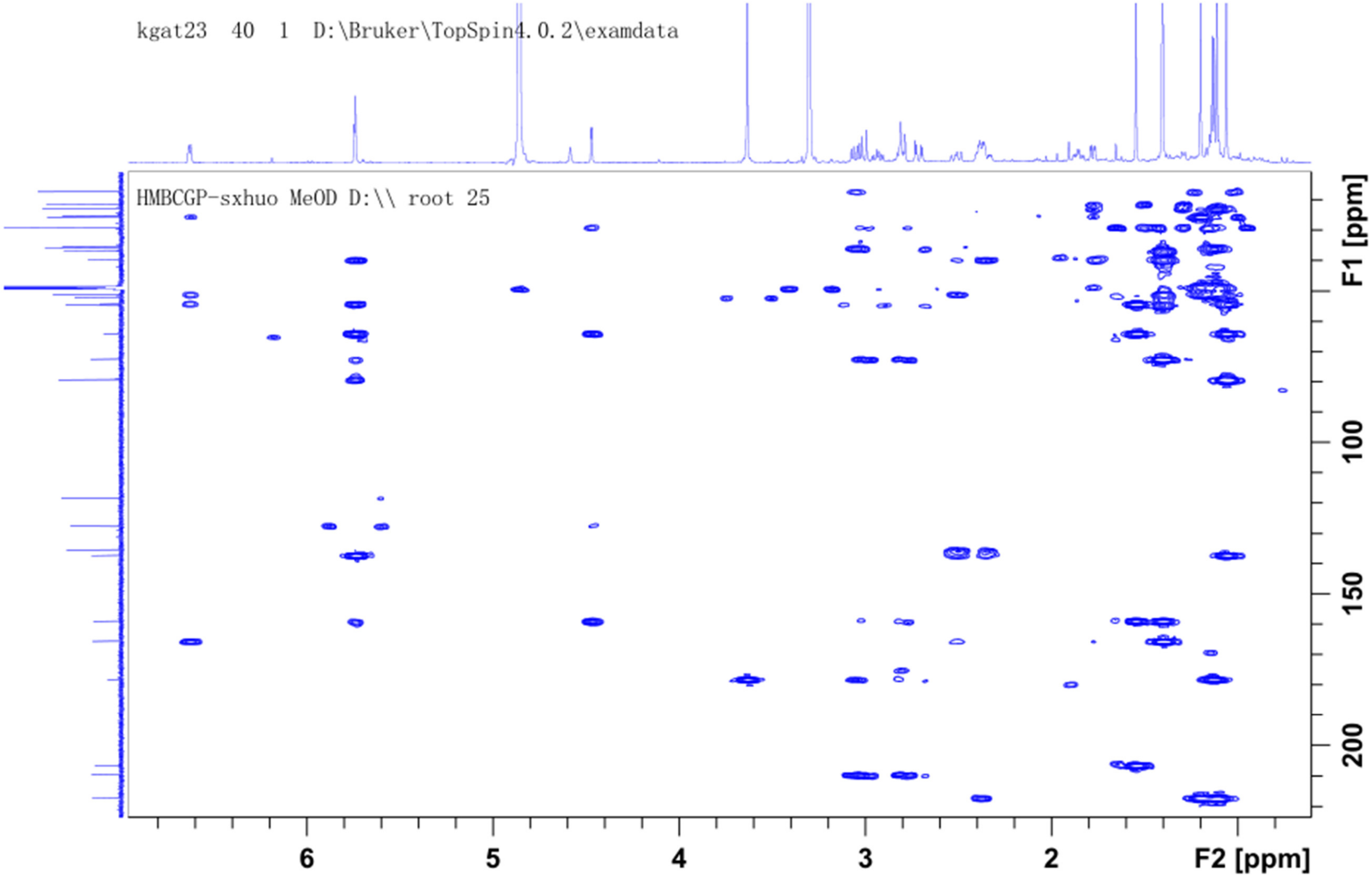

Figure S79. ROESY (600/150 MHz, MeOH-d_4) spectrum of 11.

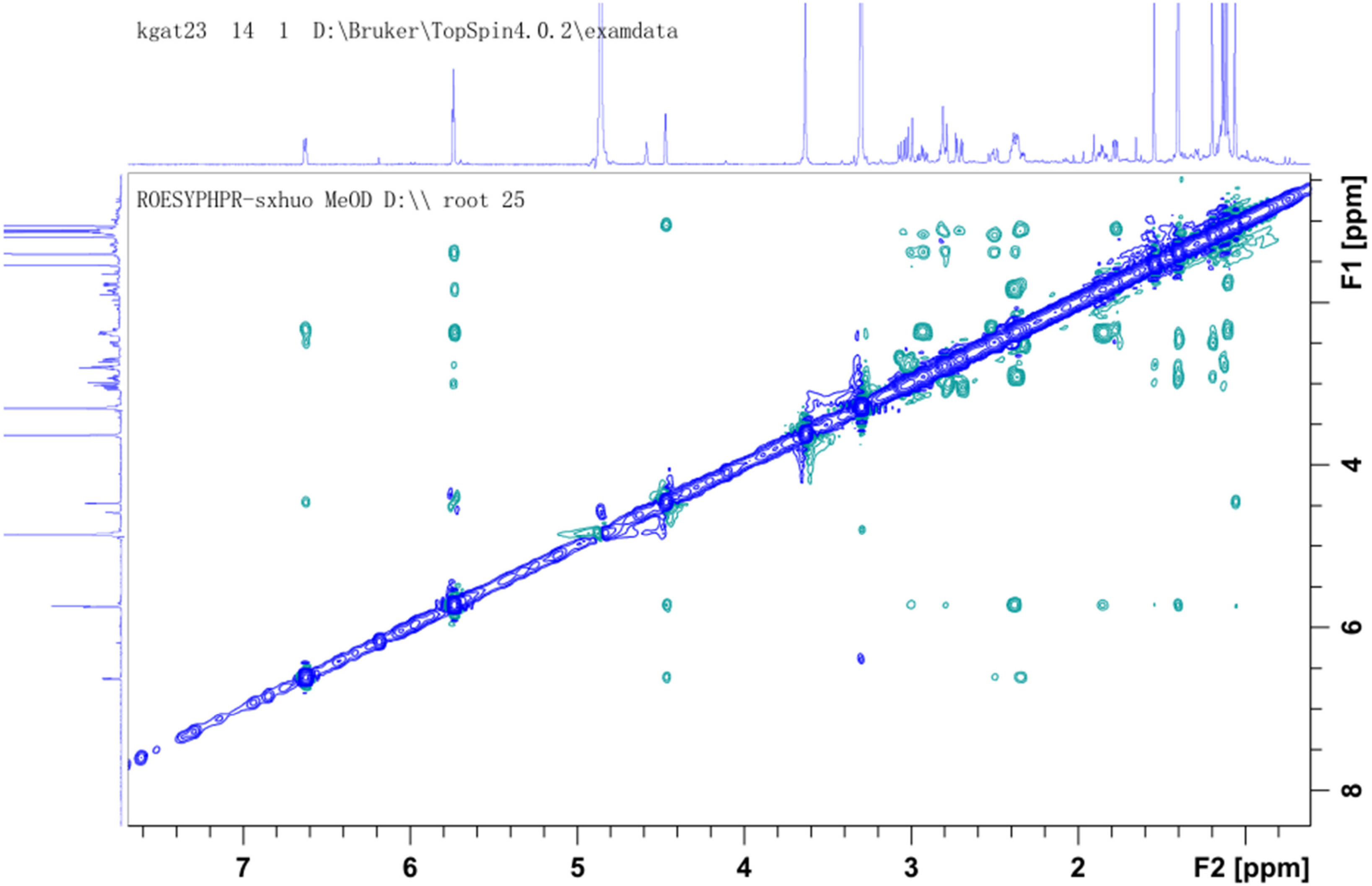

Section S24: HRESIMS spectrum of 11

Figure S80. HRESIMS spectrum of 11.

Qualitative Analysis Report

**Data Filename**	kgat23.d	**Sample Name**	kgat23
**Sample Type**	Sample	**Position**	P1-B1
**Instrument Name**	Instrument 1	**User Name**	
**Acq Method**	s.m	**Acquired Time**	2/25/2021 11:47:38 AM
**IRM Calibration Status**	Success	**DA Method**	Default.m
**Comment**			

**Sample Group**		**Info.**
**Acquisition SW**	6200 series TOF/6500 series	
**Version**	Q-TOF B.05.01 (B5125.2)	

User Spectra

Fragmentor Voltage	Collision Energy	Ionization Mode
150	0	ESI

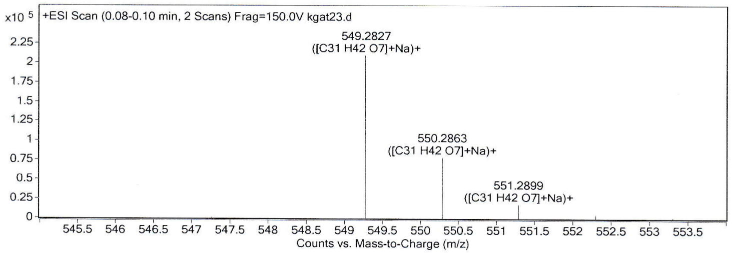

Peak List

m/z	z	Abund	Formula	Ion
146.0803	1	25692.33		
172.0937	1	58315.53		
527.3001	1	35446.52		
533.2871	1	34701.41		
549.2827	1	209133.06	C31 H42 O7	(M+Na)+
550.2863	1	78684.9	C31 H42 O7	(M+Na)+
698.3877	1	23987.68		
1075.577	1	124756.34		
1076.5805	1	99580.17		
1077.5836	1	41677.77		

Formula Calculator Element Limits

Element	Min	Max
C	3	60
H	0	120
O	0	30

Formula Calculator Results

Formula	CalculatedMass	CalculatedMz	Mz	Diff. (mDa)	Diff. (ppm)	DBE
C31 H42 O7	526.2931	549.2823	549.2827	-0.40	-0.73	11.0000

--- End Of Report ---

Agilent Technologies　Page 1 of 1　Printed at: 2:02 PM on: 2/25/2021

Section S25: 1D and 2D NMR spectra of compound 12

Figure S81. ^{1}H NMR (600 MHz, MeOH-d_4) spectrum of 12.

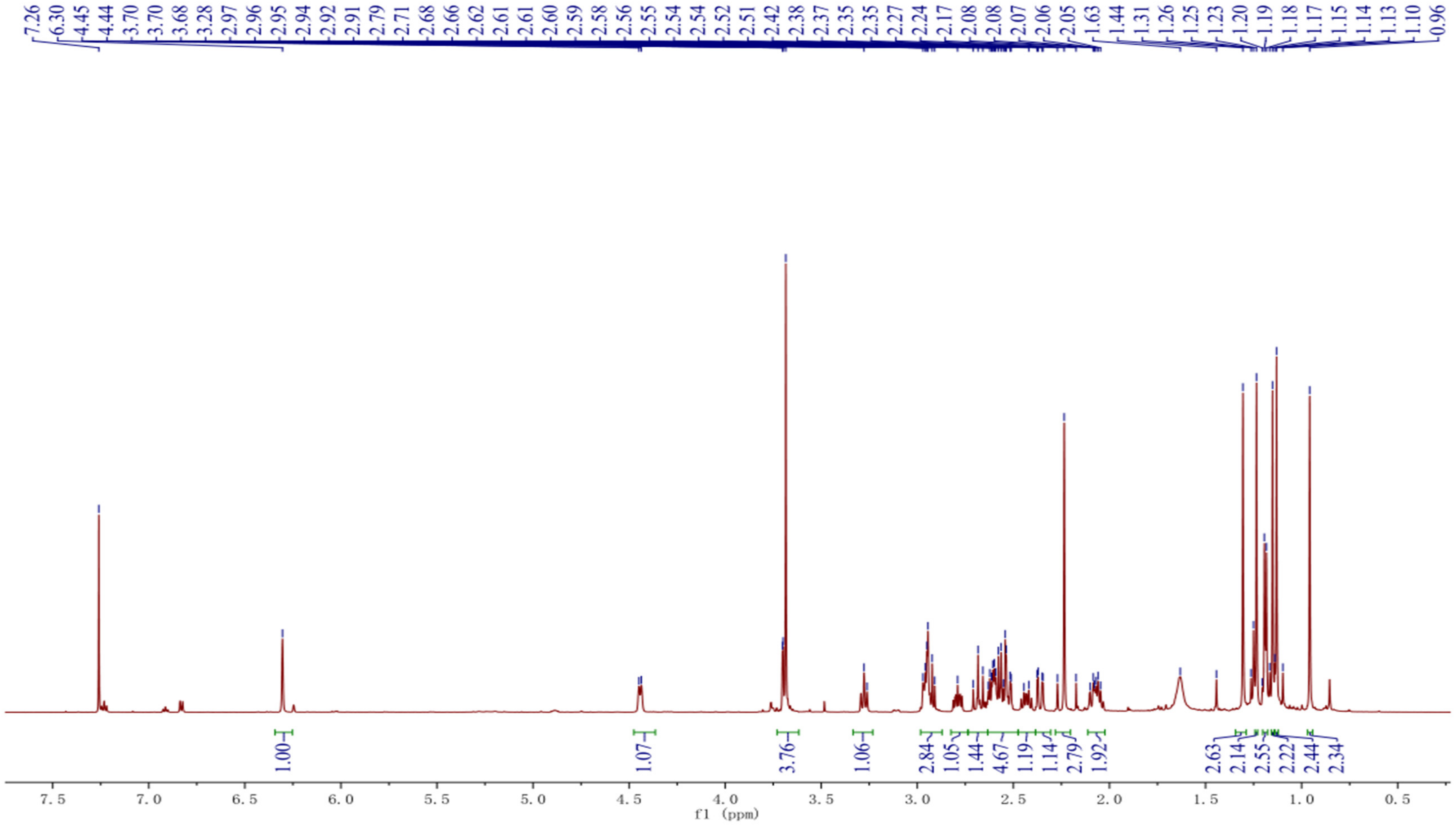

Figure S82. ^{13}C NMR (150 MHz, MeOH-d_4) spectrum of 12.

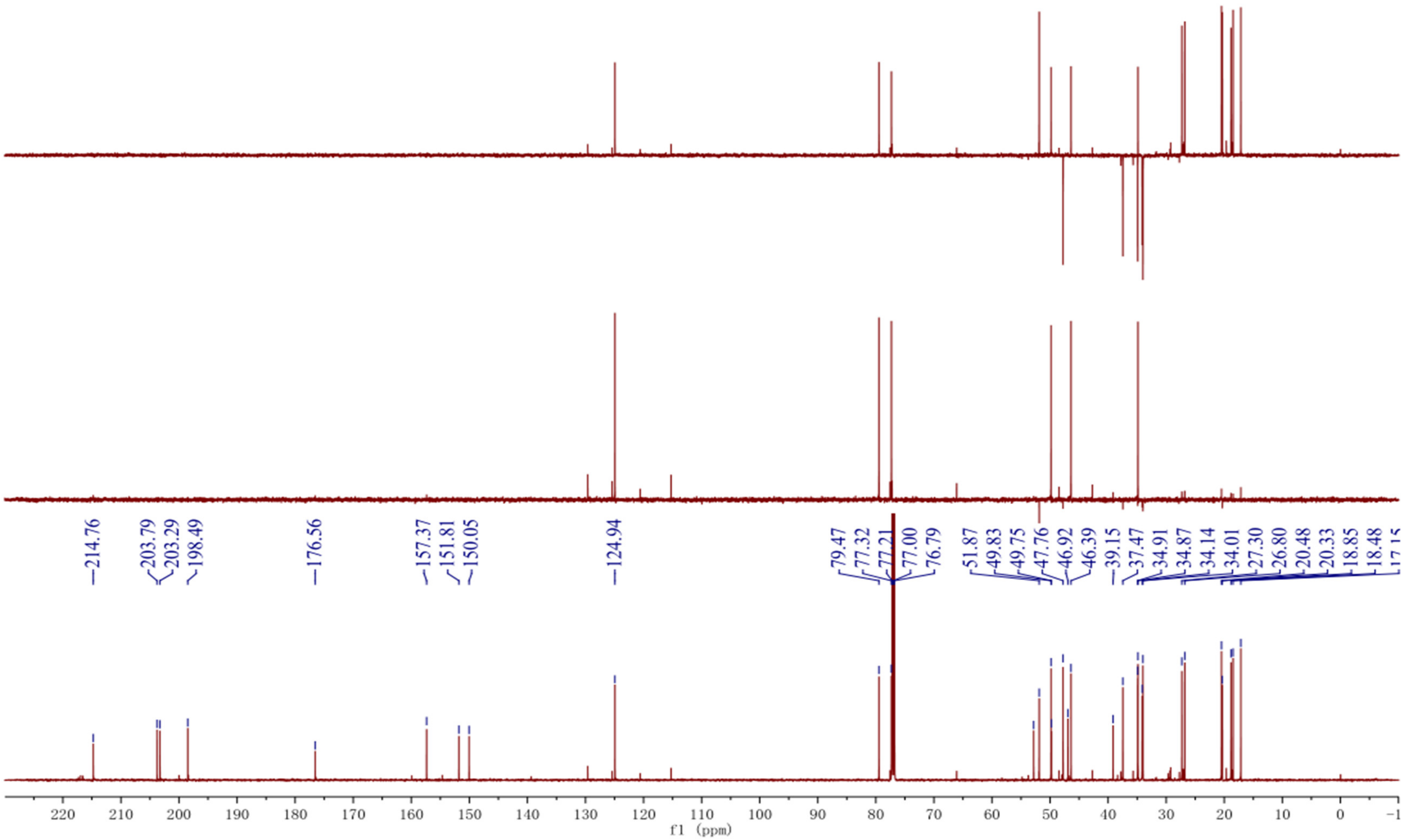

Figure S83. ^{1}**H-**^{1}**H COSY (600 MHz, MeOH-*d*$_4$) spectrum of 12.**

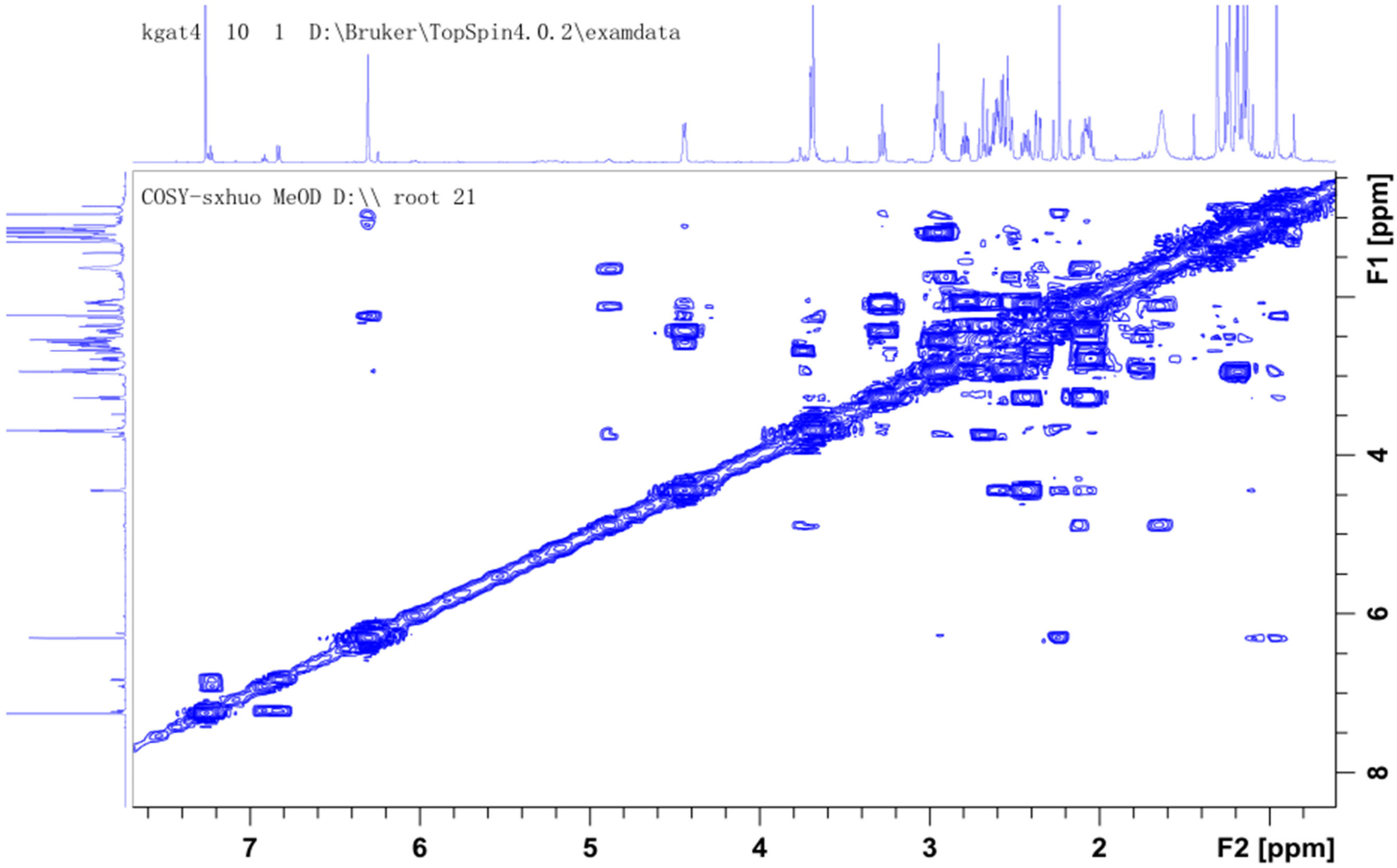

Figure S84. HSQC (600/150 MHz, MeOH-d_4) spectrum of 12.

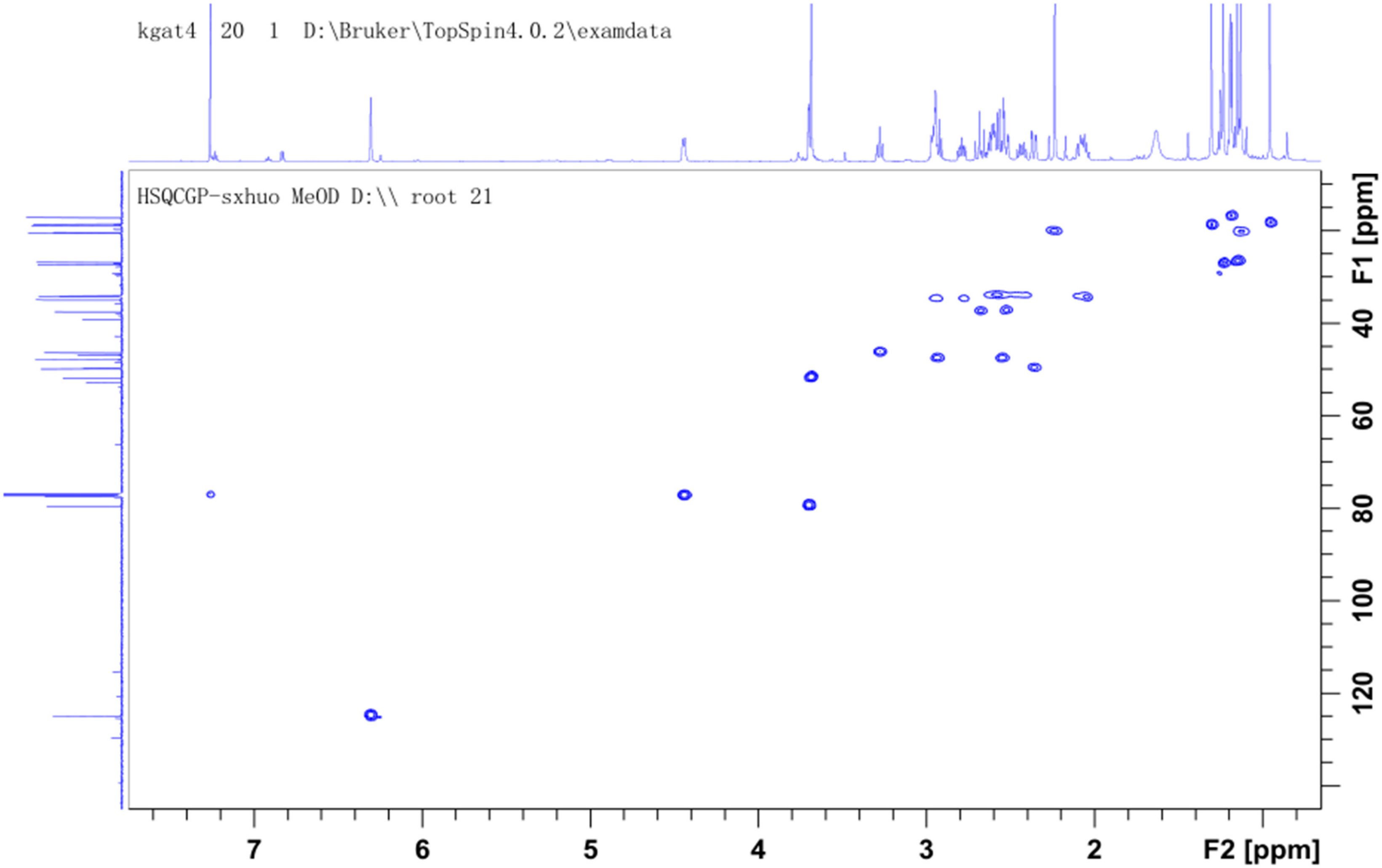

Figure S85. HMBC (600/150 MHz, MeOH-d_4) spectrum of 12.

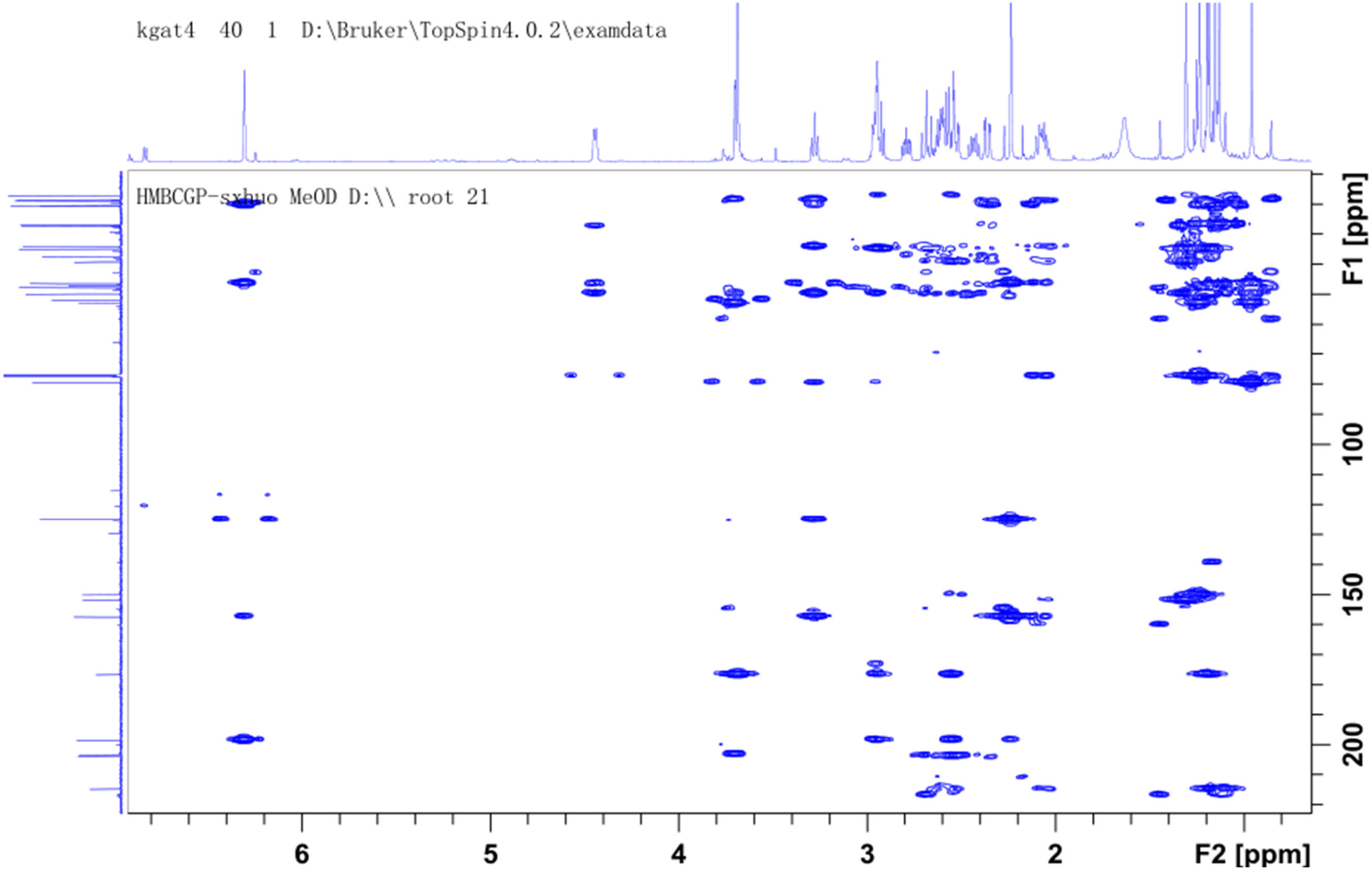

Figure S86. ROESY (600/150 MHz, MeOH-d_4) spectrum of 12.

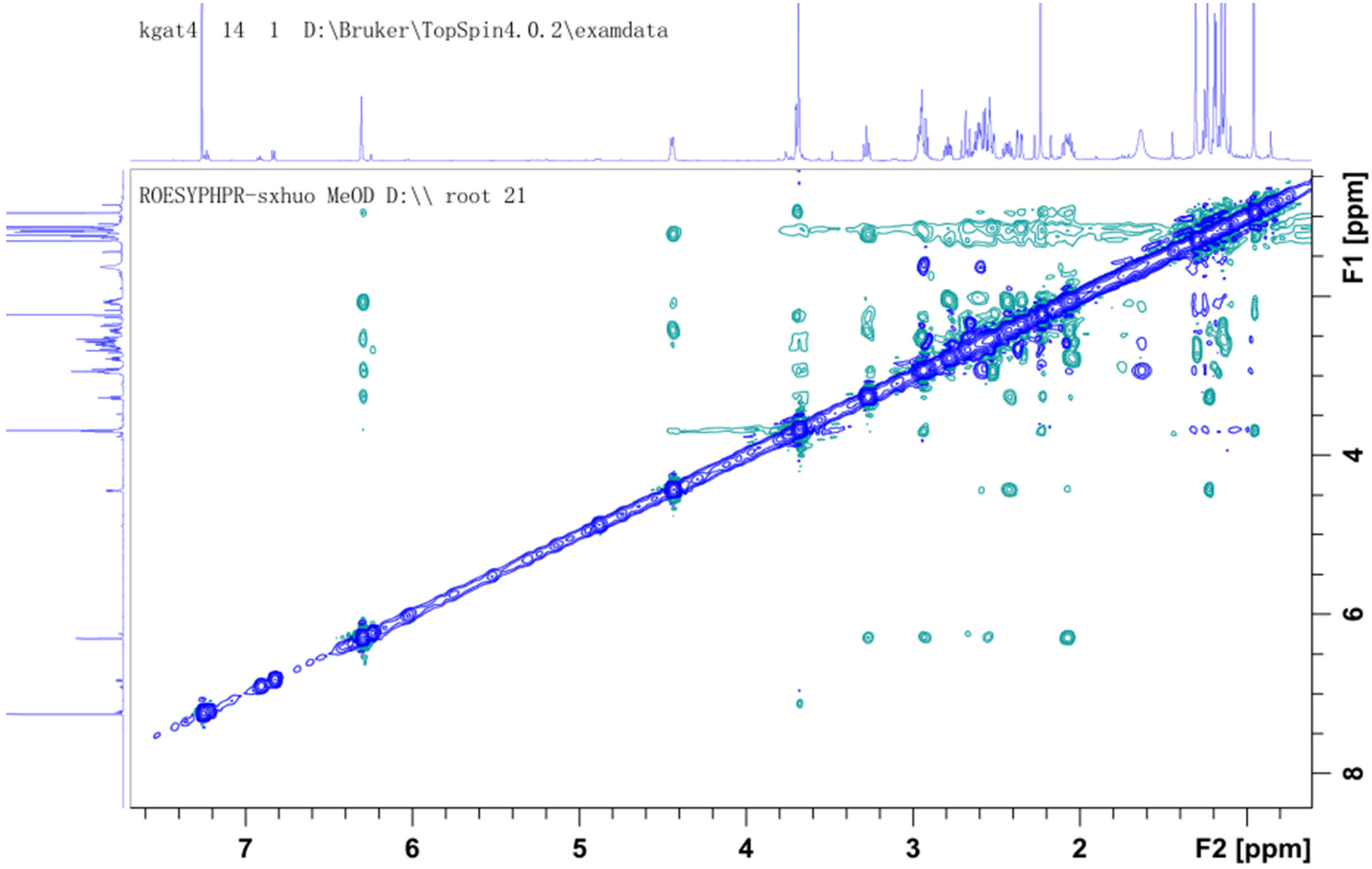

Section S26: HRESIMS spectrum of 12

Figure S87. HRESIMS spectrum of 12.

Qualitative Analysis Report

**Data Filename**	kgat4.d	**Sample Name**	kgat4
**Sample Type**	Sample	**Position**	P1-A4
**Instrument Name**	Instrument 1	**User Name**	
**Acq Method**	s.m	**Acquired Time**	2/25/2021 11:40:32 AM
**IRM Calibration Status**	Success	**DA Method**	Default.m
**Comment**			

Sample Group **Info.**

Acquisition SW 6200 series TOF/6500 series
Version Q-TOF B.05.01 (B5125.2)

User Spectra

Fragmentor Voltage	Collision Energy	Ionization Mode
150	0	ESI

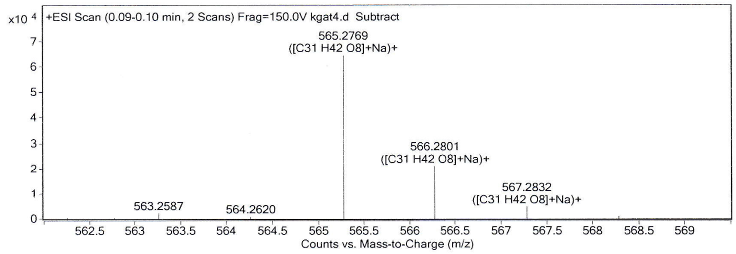

Peak List

m/z	z	Abund	Formula	Ion
102.1274	1	16389.11		
533.2869	1	4995.39		
543.2943	1	9074.71		
565.2769	1	64496.13	C31 H42 O8	(M+Na)+
566.2801	1	20900.9	C31 H42 O8	(M+Na)+
581.2508	1	13391.67		
582.2538	1	5103.63		
1107.5652	1	44161.14		
1108.5683	1	28777.86		
1109.5723	1	10624.63		

Formula Calculator Element Limits

Element	Min	Max
C	3	60
H	0	120
O	0	30

Formula Calculator Results

Formula	CalculatedMass	CalculatedMz	Mz	Diff. (mDa)	Diff. (ppm)	DBE
C31 H42 O8	542.2880	565.2772	565.2769	0.30	0.53	11.0000

--- End Of Report ---

Section S27: 1D and 2D NMR spectra of compound 13

Figure S88. ^{1}**H NMR (600 MHz, MeOH-**d_4**) spectrum of 13.**

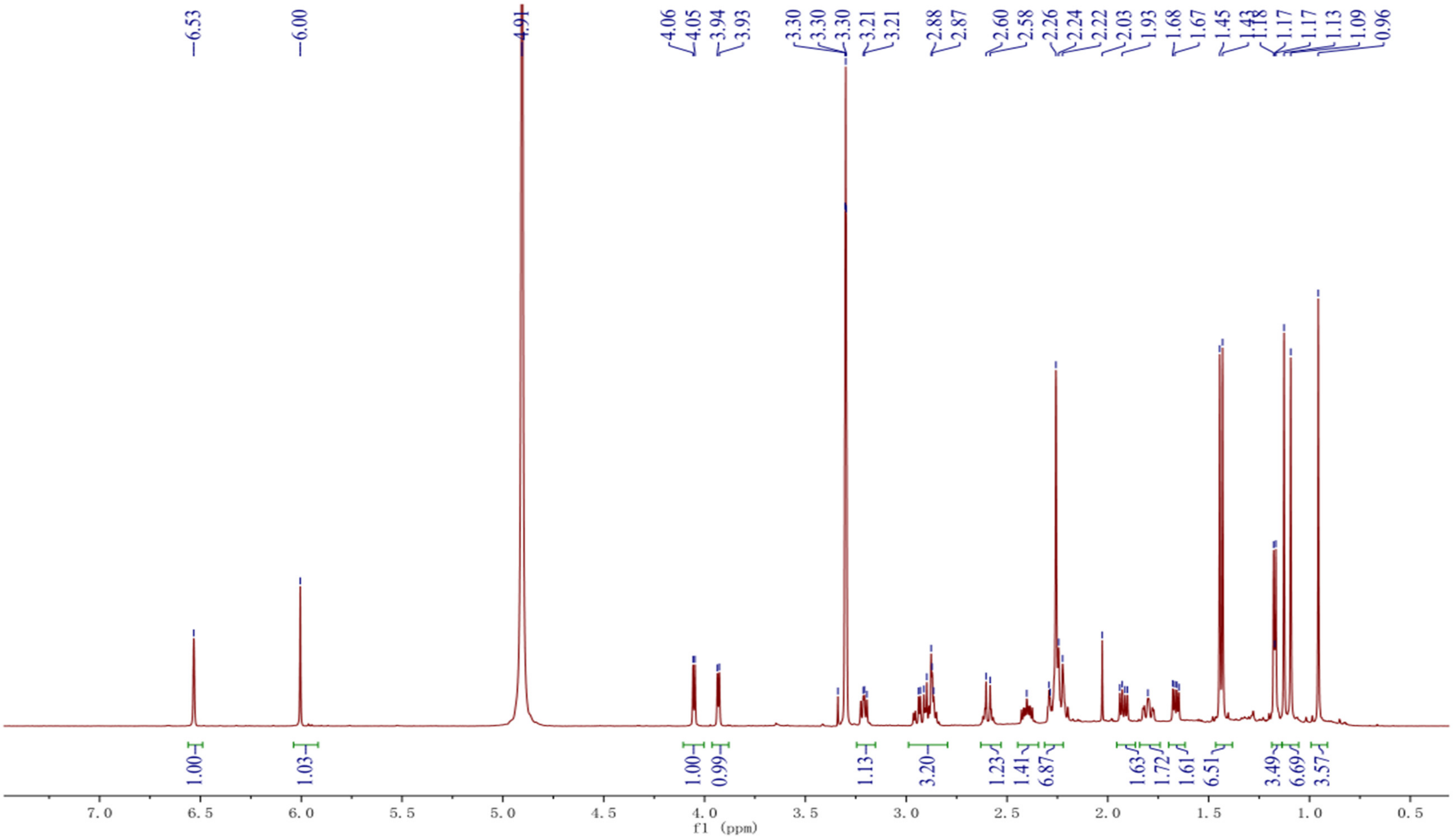

Figure S89. ^{13}**C NMR (150 MHz, MeOH-*d*$_4$) spectrum of 13.**

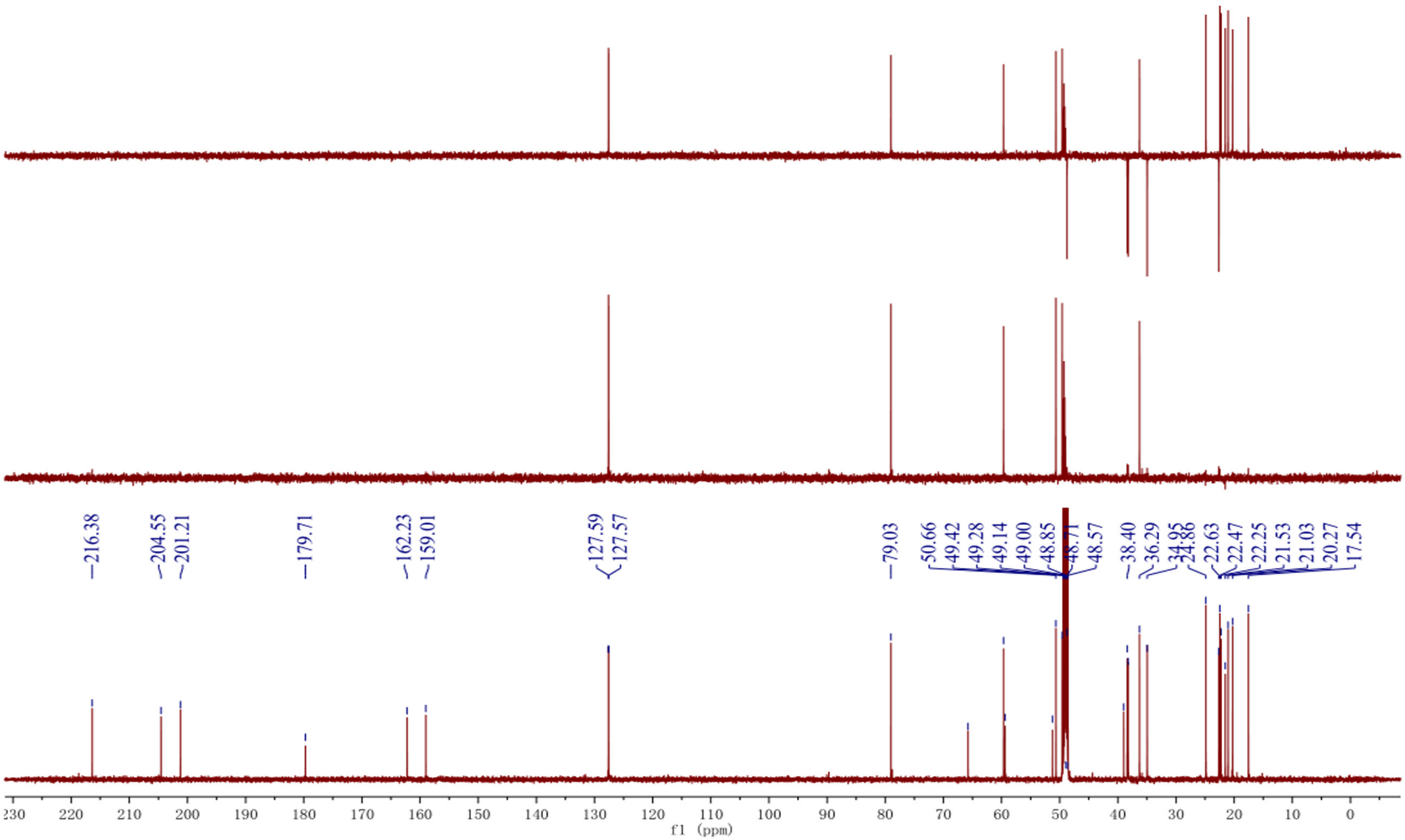

Figure S90. ^{1}H-^{1}H **COSY (600 MHz, MeOH-d_4) spectrum of 13.**

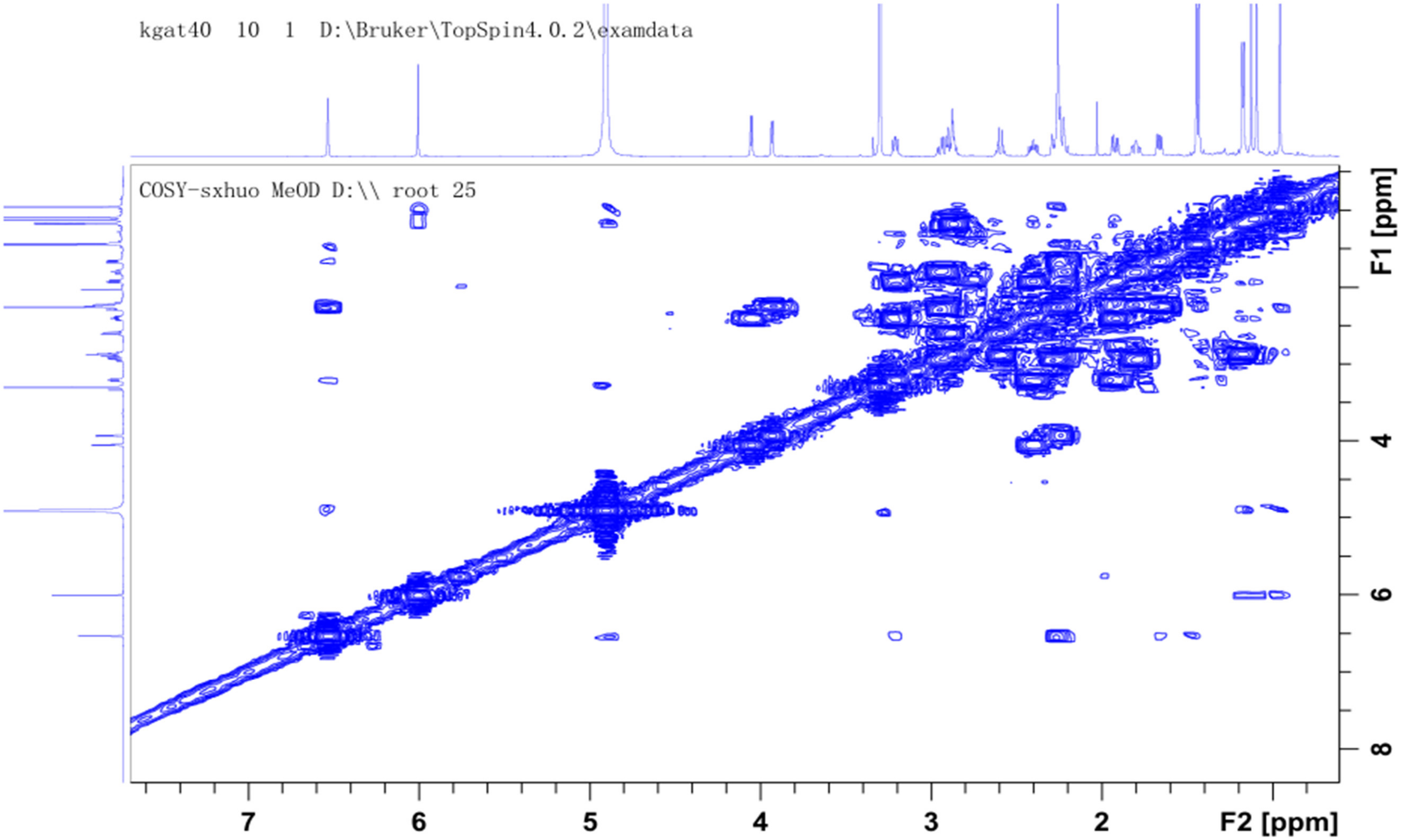

Figure S91. HSQC (600/150 MHz, MeOH-d_4) spectrum of 13.

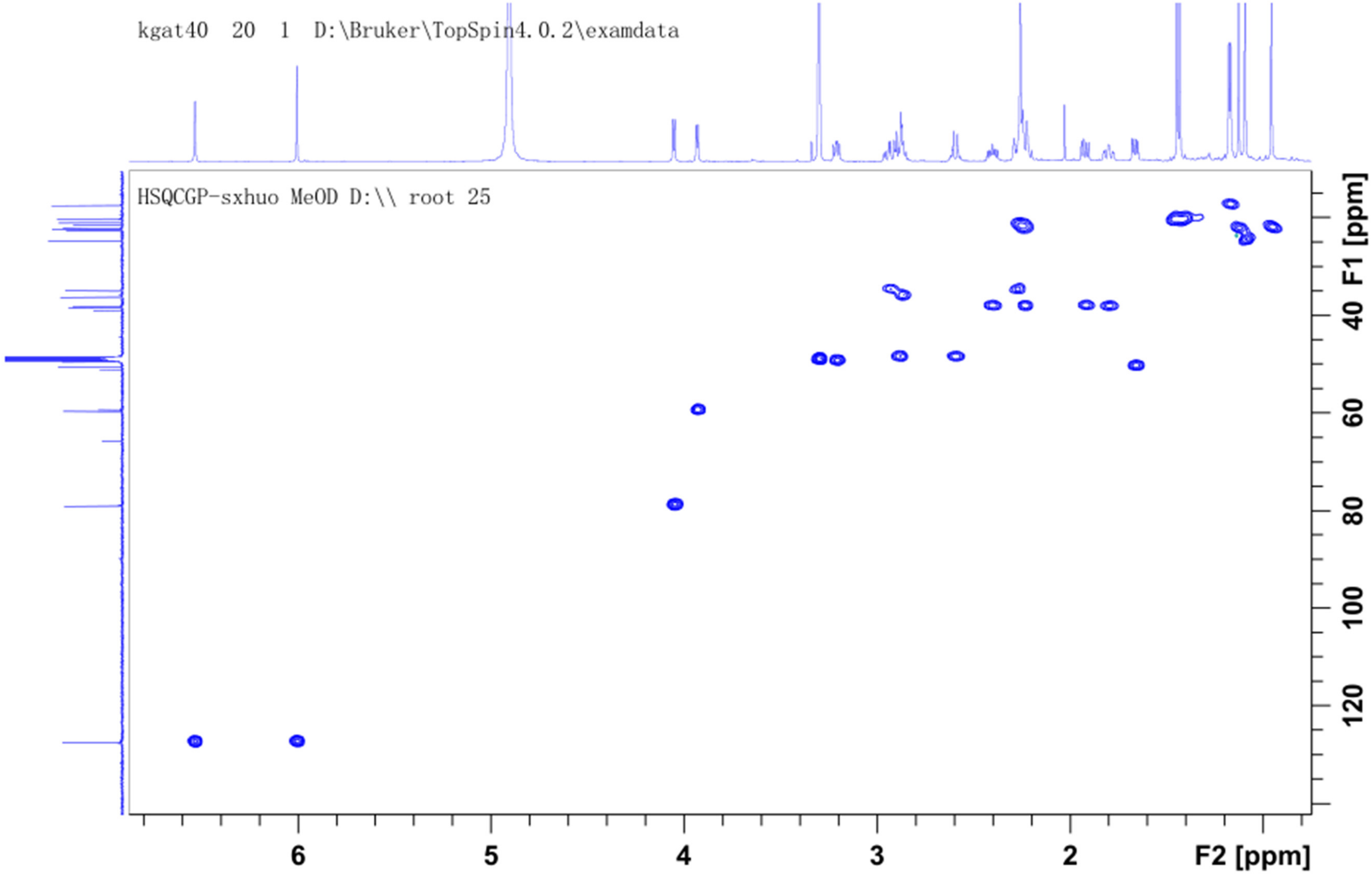

Figure S92. HMBC (600/150 MHz, MeOH-d_4) spectrum of 13.

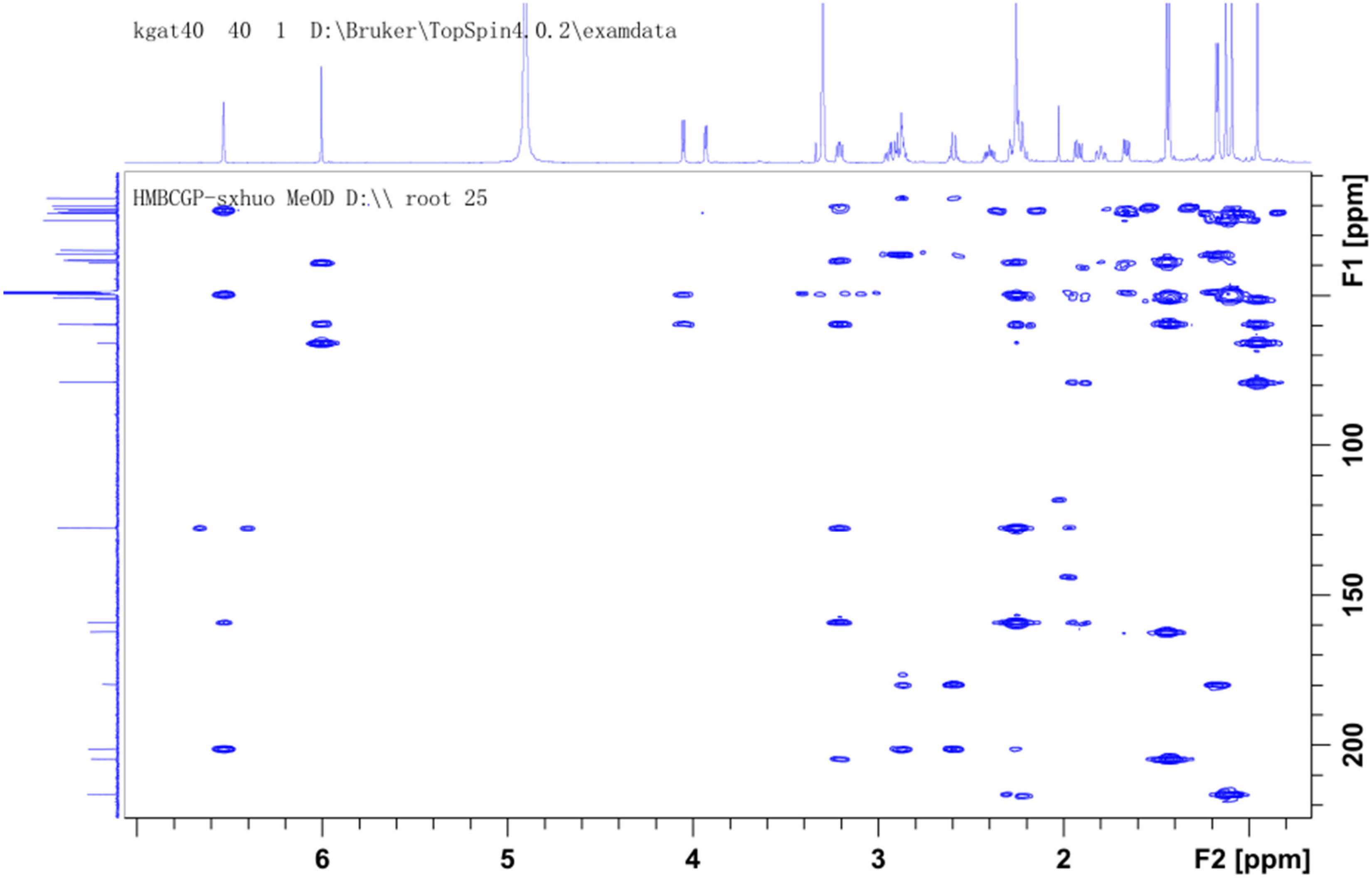

Figure S93. ROESY (600/150 MHz, MeOH-d_4) spectrum of 13.

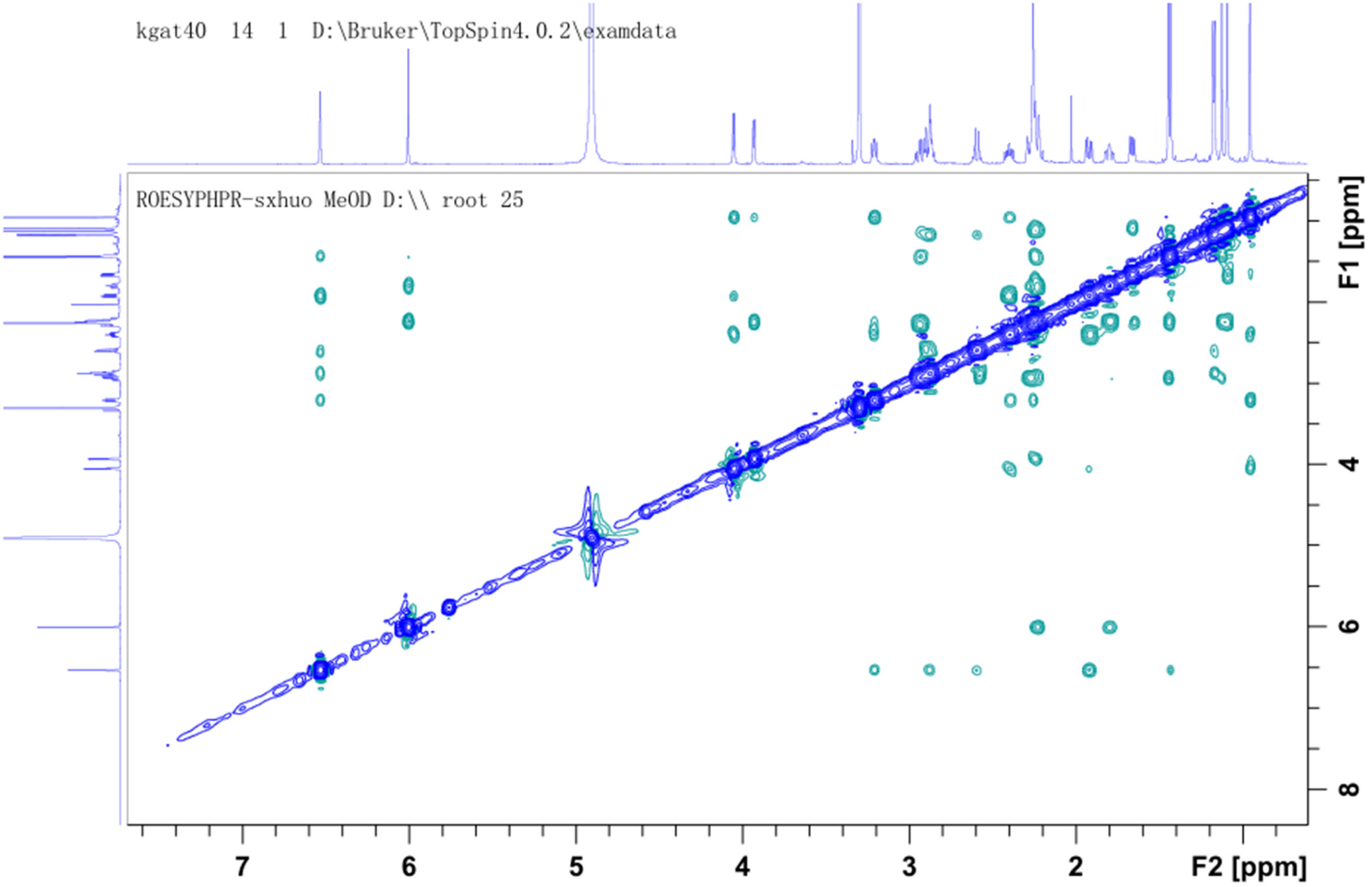

Section S28: HRESIMS spectrum of 13

Figure S94. HRESIMS spectrum of 13.

Qualitative Analysis Report

**Data Filename**	kgat40.d	**Sample Name**	kgat40
**Sample Type**	Sample	**Position**	P1-B4
**Instrument Name**	Instrument 1	**User Name**	
**Acq Method**	s.m	**Acquired Time**	2/25/2021 2:06:00 PM
**IRM Calibration Status**	Success	**DA Method**	Default.m
**Comment**			

Info.

**Sample Group**	
**Acquisition SW Version**	6200 series TOF/6500 series Q-TOF B.05.01 (B5125.2)

User Spectra

Fragmentor Voltage	Collision Energy	Ionization Mode
150	0	ESI

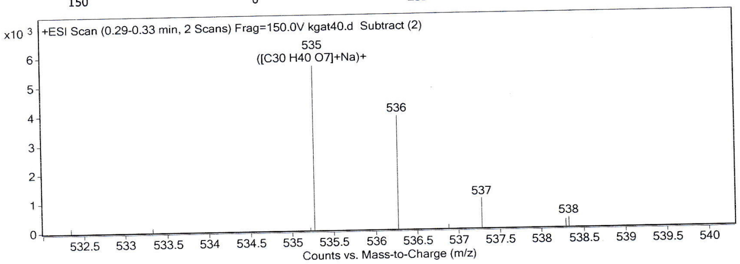

Peak List

m/z	z	Abund	Formula	Ion
535		5665.46	C30 H40 O7	(M+Na)+
536	1	3920.02		
552	1	19353.48		
553	1	11162.89		
554	1	3792.42		
557	1	2477.68		
568	1	7182.2		
569	1	3411.75		
701	1	4822.14		
702	1	3227.21		

Formula Calculator Element Limits

Element	Min	Max
C	3	60
H	0	120
O	0	30

Formula Calculator Results

Formula	CalculatedMass	CalculatedMz	Mz	Diff. (mDa)	Diff. (ppm)	DBE
C30 H40 O7	512.2774	535.0000	535.0000	0.00	0.00	11.0000

--- End Of Report ---

Agilent Technologies | Page 1 of 1 | Printed at: 2:09 PM on: 2/25/2021

Section S29: 1D and 2D NMR spectra of compound 14

Figure S95. ^{1}H NMR (600 MHz, MeOH-d_4) spectrum of 14.

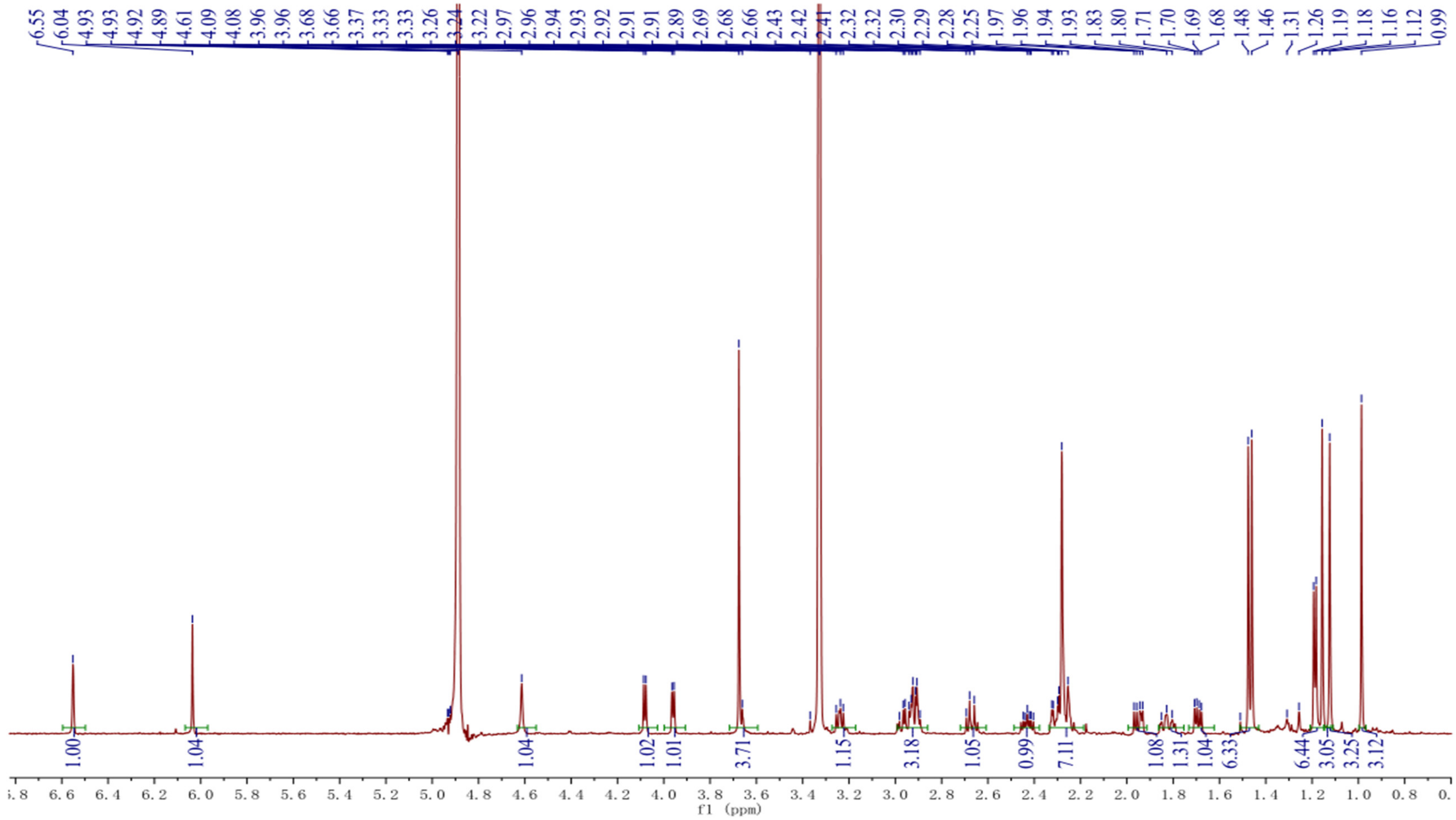

Figure S96. ^{13}**C NMR (150 MHz, MeOH-*d*$_4$) spectrum of 14.**

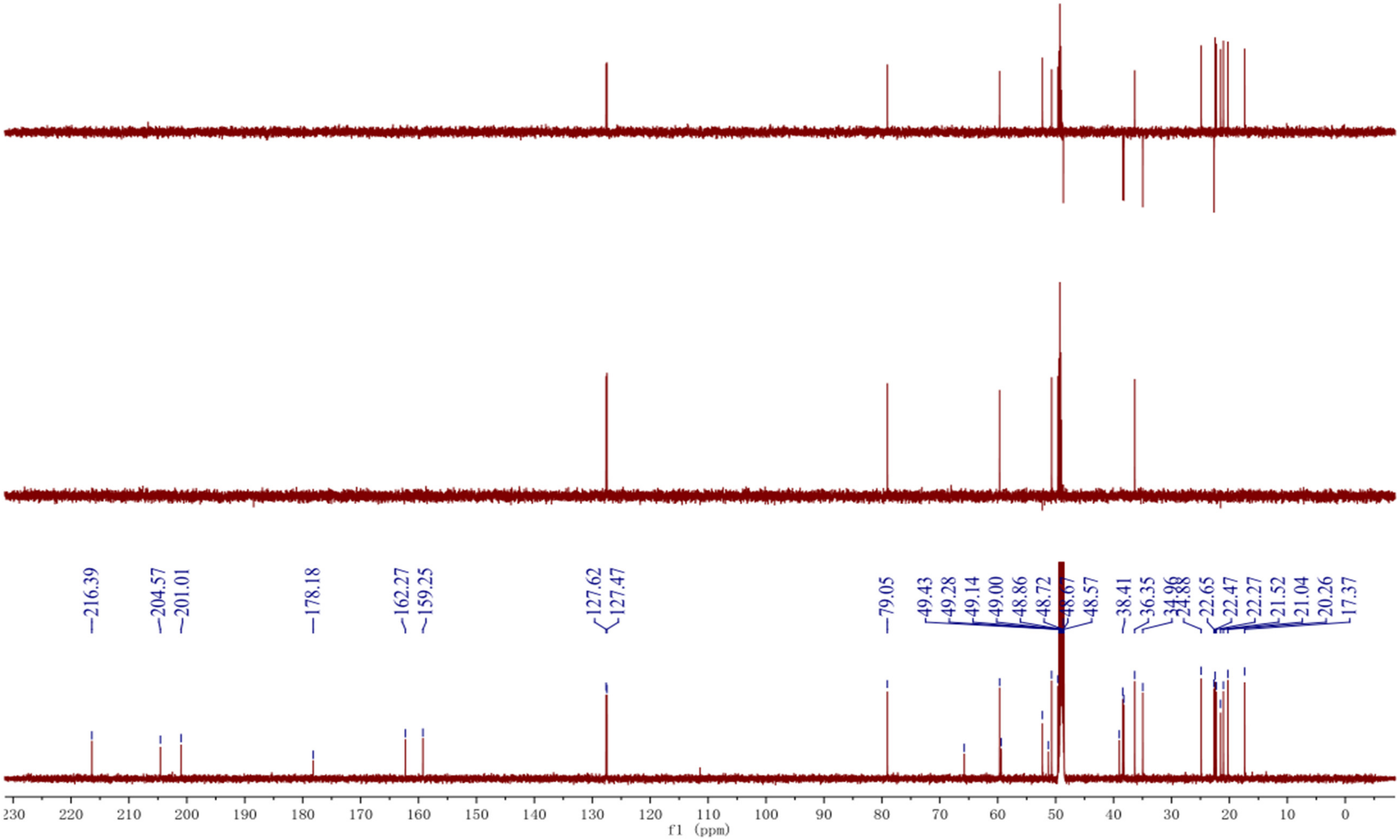

Figure S97. **^{1}H-^{1}H COSY (600 MHz, MeOH-d_4) spectrum of 14.**

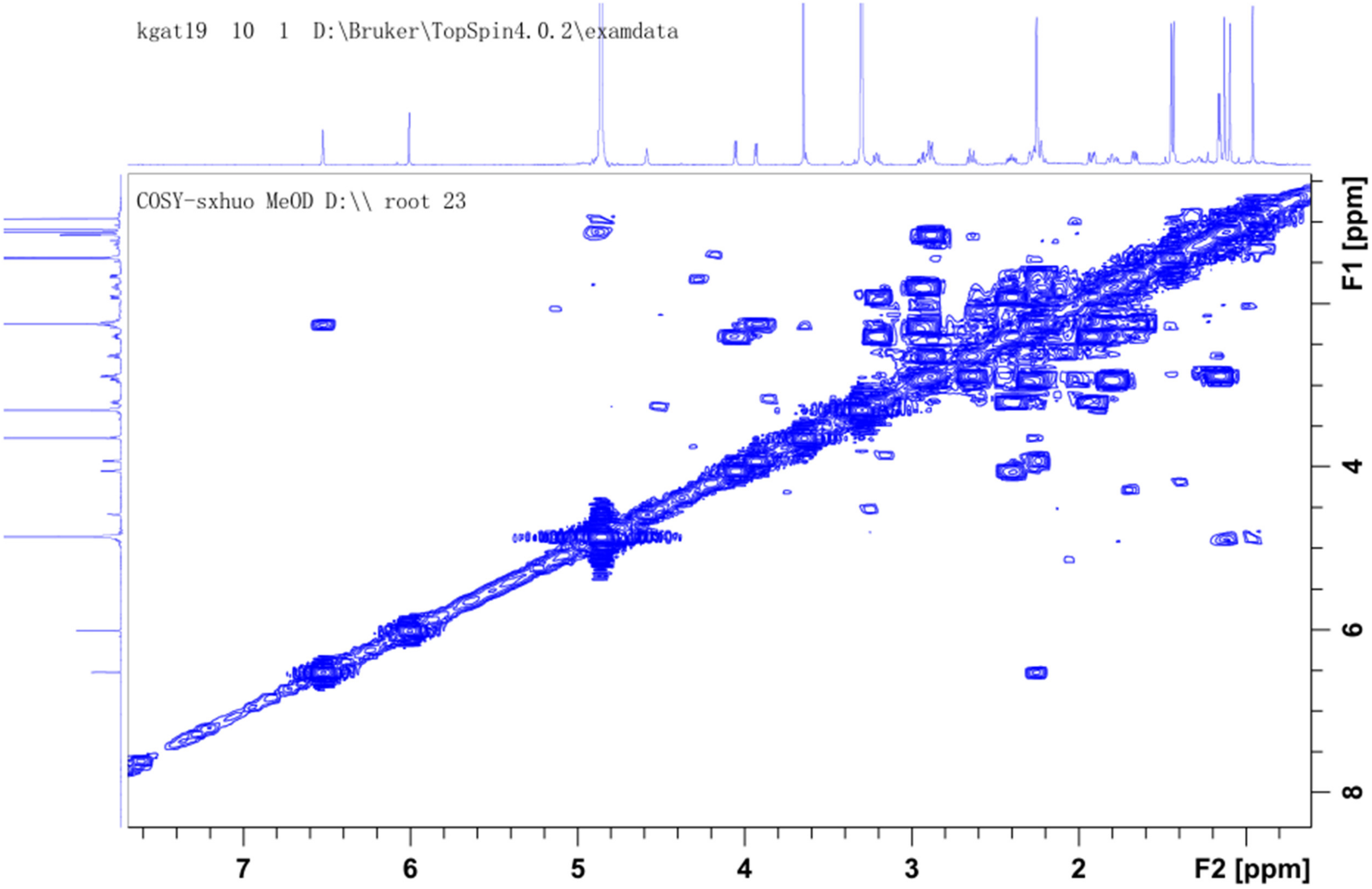

Figure S98. HSQC (600/150 MHz, MeOH-d_4) spectrum of 14.

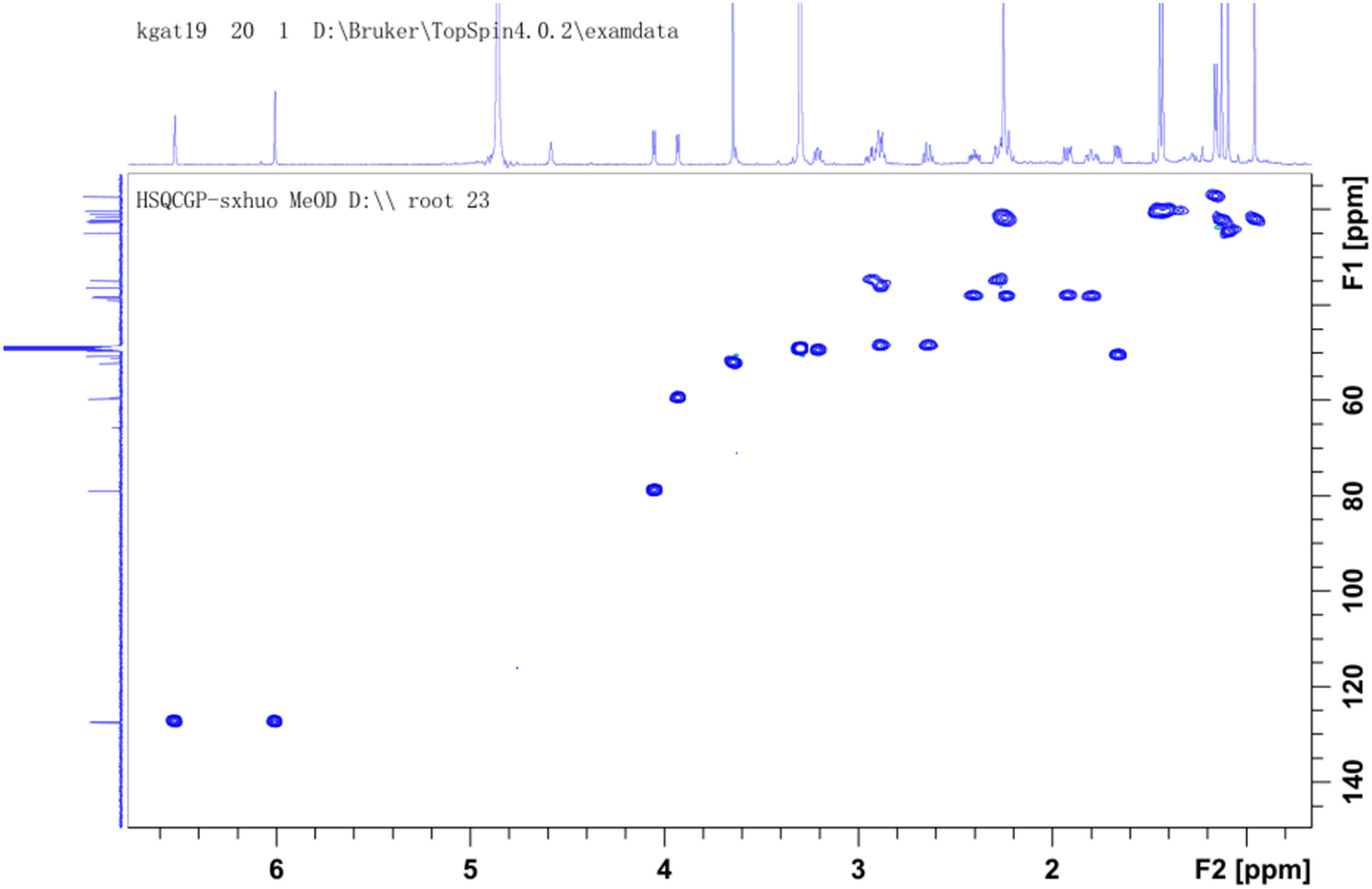

Figure S99. HMBC (600/150 MHz, MeOH-d_4) spectrum of 14.

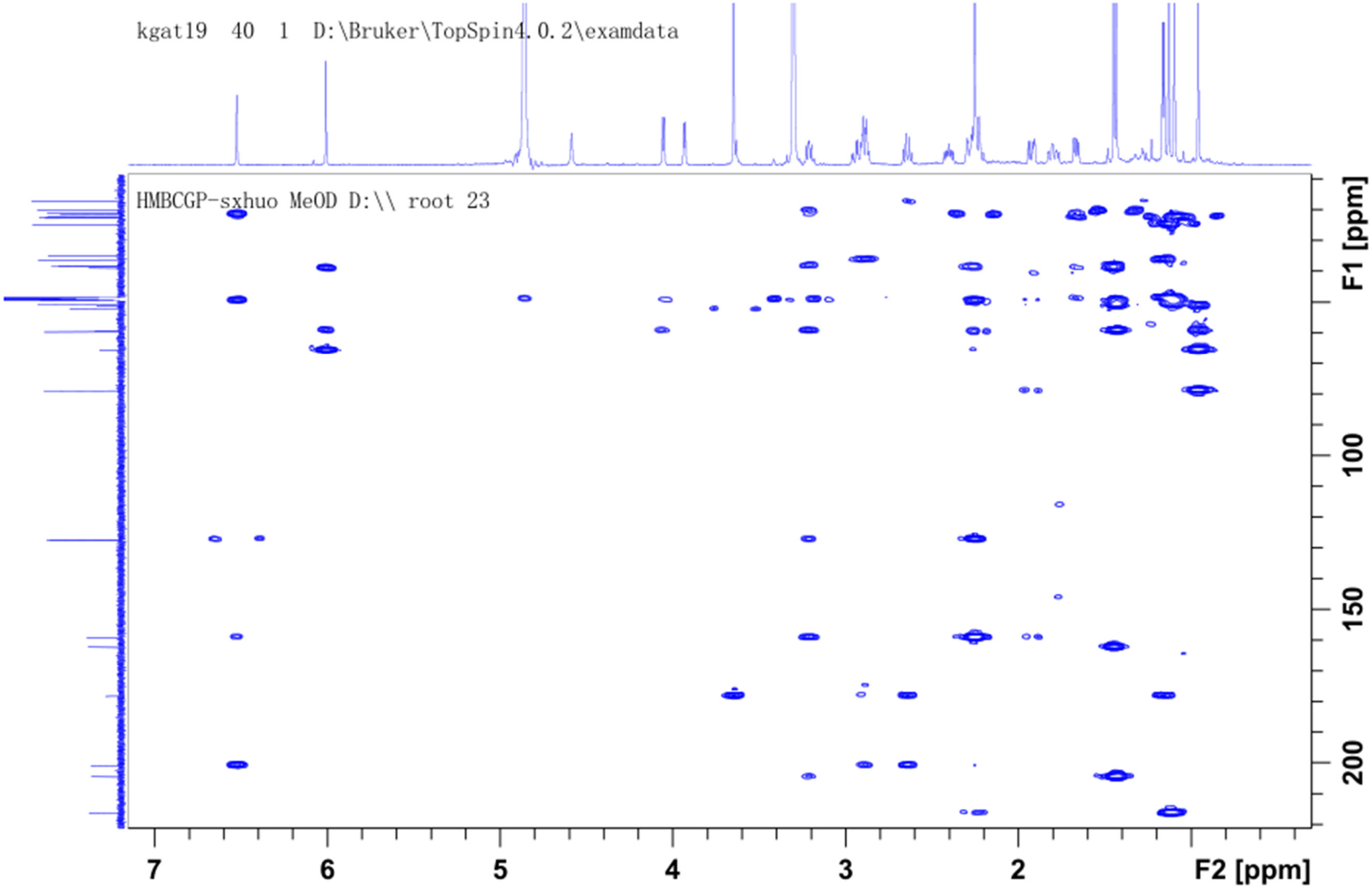

Figure S100. ROESY (600/150 MHz, MeOH-d_4) spectrum of 14.

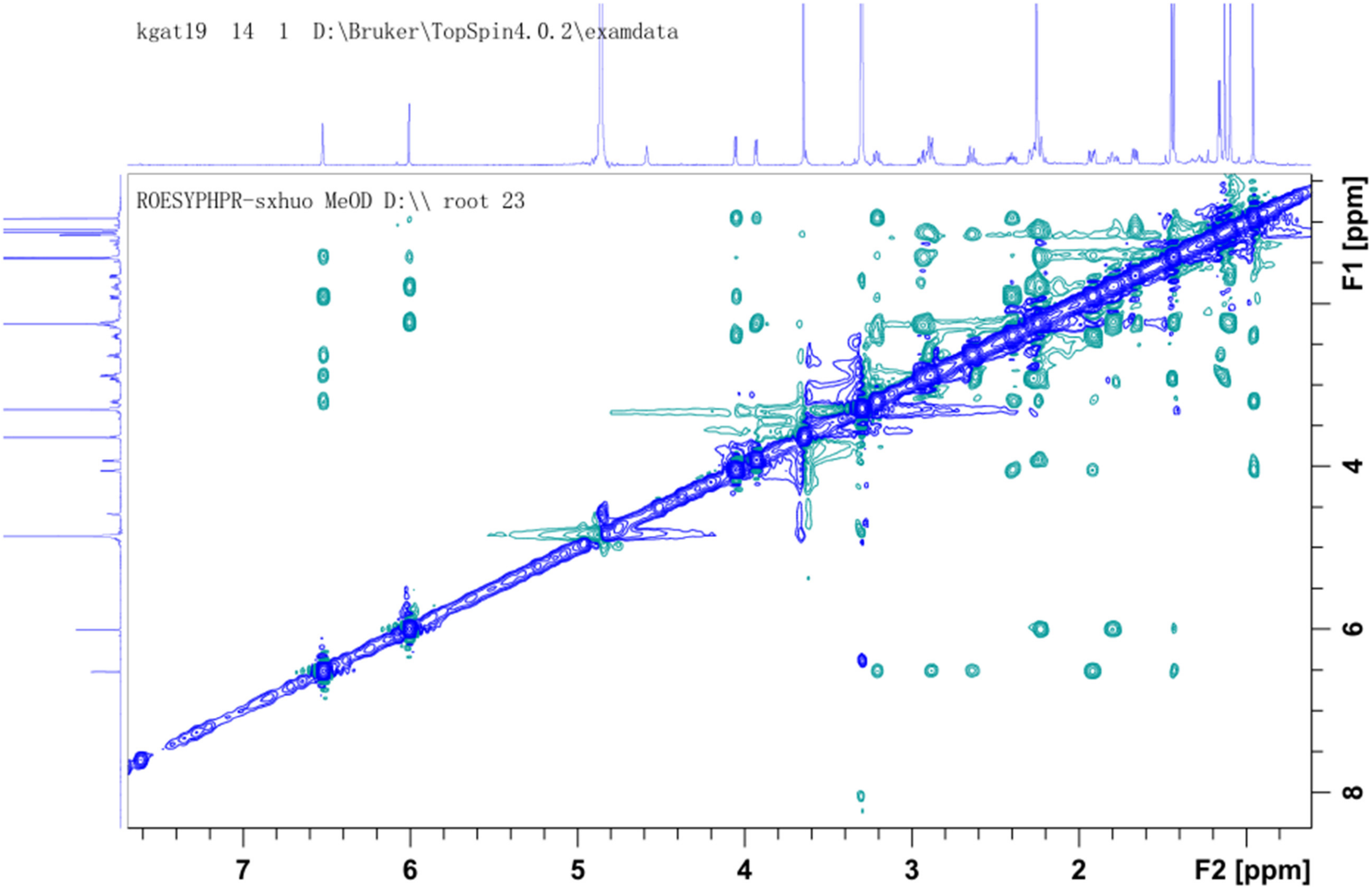

Section S30: HRESIMS spectrum of 14

Figure S101. HRESIMS spectrum of 14.

Qualitative Analysis Report

**Data Filename**	kgat19.d	**Sample Name**	kgat19
**Sample Type**	Sample	**Position**	P1-A8
**Instrument Name**	Instrument 1	**User Name**	
**Acq Method**	s.m	**Acquired Time**	2/25/2021 11:45:17 AM
**IRM Calibration Status**	Success	**DA Method**	Default.m
**Comment**			

Sample Group **Info.**

Acquisition SW Version 6200 series TOF/6500 series Q-TOF B.05.01 (B5125.2)

User Spectra

Fragmentor Voltage	Collision Energy	Ionization Mode
150	0	ESI

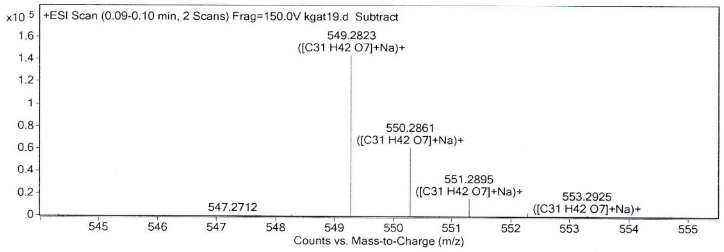

Peak List

m/z	z	Abund	Formula	Ion
535.3027	1	41472.34		
536.3062	1	16993.7		
549.2823	1	144187.16	C31 H42 O7	(M+Na)+
550.2861	1	61901.48	C31 H42 O7	(M+Na)+
551.2895	1	15919.39	C31 H42 O7	(M+Na)+
565.2684	1	22792.32		
698.3868	1	20275.38		
1061.5959	1	17146.34		
1075.5751	1	36301.75		
1076.5791	1	32261.56		

Formula Calculator Element Limits

Element	Min	Max
C	3	60
H	0	120
O	0	30

Formula Calculator Results

Formula	CalculatedMass	CalculatedMz	Mz	Diff. (mDa)	Diff. (ppm)	DBE
C31 H42 O7	526.2931	549.2823	549.2823	0.00	0.00	11.0000

--- End Of Report ---

Agilent Technologies Page 1 of 1 Printed at: 2:01 PM on: 2/25/2021

Section S31: 1D and 2D NMR spectra of compound 15

Figure S102. Comparison of the ^{1}H NMR (600 MHz, MeOH-d_4) spectra of 15 and 28.

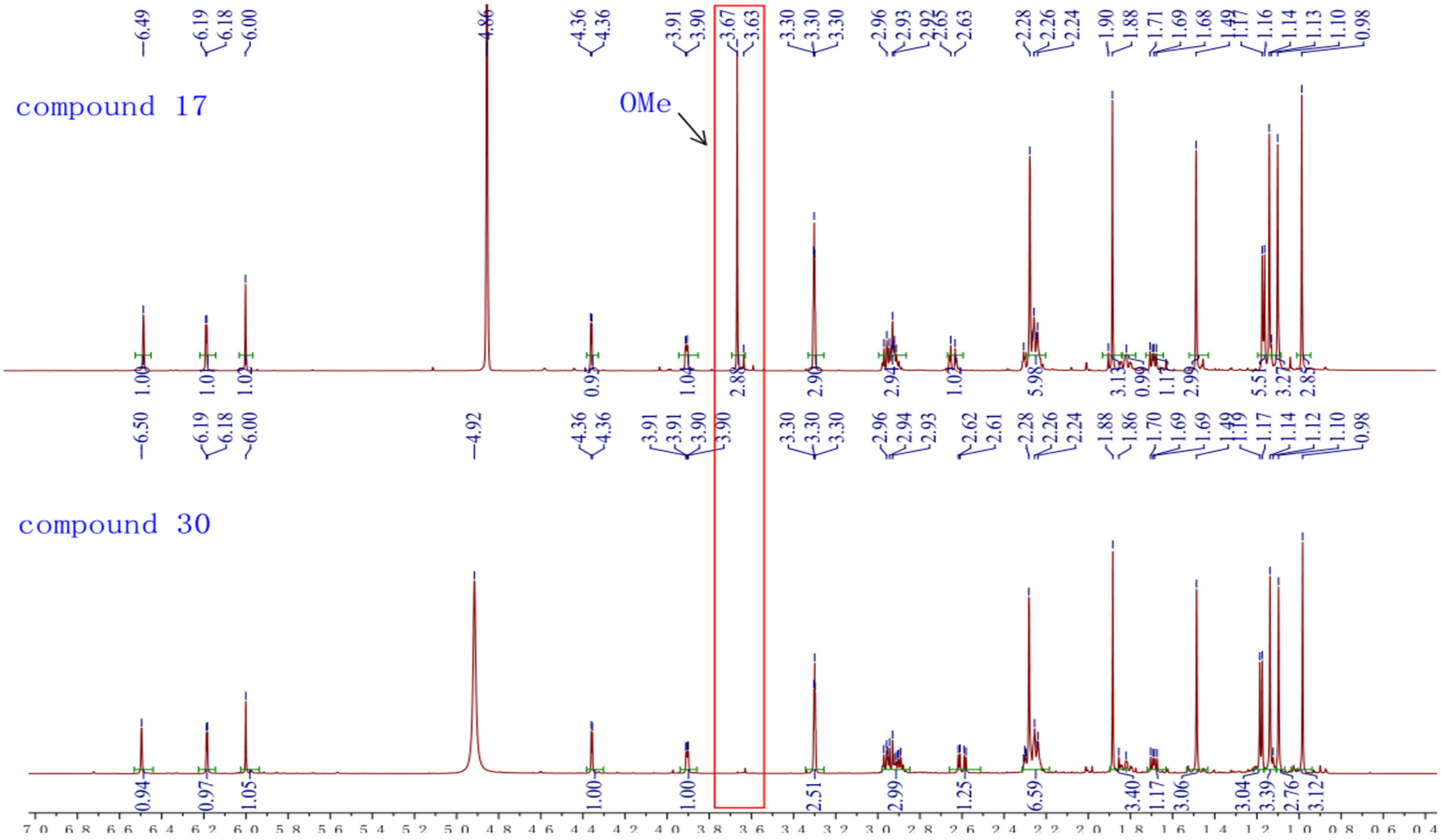

Section S32: HRESIMS spectrum of 15

Figure S103. HRESIMS spectrum of 15.

Qualitative Analysis Report

**Data Filename**	kgat24.d	**Sample Name**	kgat24
**Sample Type**	Sample	**Position**	P1-B2
**Instrument Name**	Instrument 1	**User Name**	
**Acq Method**	s.m	**Acquired Time**	2/25/2021 11:48:49 AM
**IRM Calibration Status**	Success	**DA Method**	Default.m
**Comment**			

**Sample Group**		**Info.**
**Acquisition SW Version**	6200 series TOF/6500 series Q-TOF B.05.01 (B5125.2)	

User Spectra

Fragmentor Voltage	Collision Energy	Ionization Mode
150	0	ESI

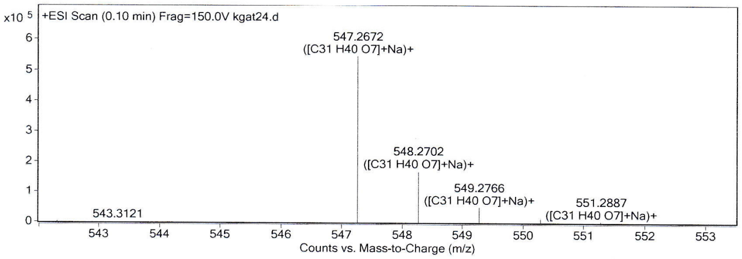

Peak List

m/z	z	Abund	Formula	Ion
172.0937	1	58406.09		
507.2738	1	47417.16		
547.2672	1	545135.31	C31 H40 O7	(M+Na)+
548.2702	1	169740.53	C31 H40 O7	(M+Na)+
549.2766	1	49009.27	C31 H40 O7	(M+Na)+
563.2411	1	65920.91		
696.372	1	58927.06		
1071.5453	1	236543.08		
1072.5485	1	156751.8		
1073.5541	1	76835.13		

Formula Calculator Element Limits

Element	Min	Max
C	3	60
H	0	120
O	0	30

Formula Calculator Results

Formula	CalculatedMass	CalculatedMz	Mz	Diff. (mDa)	Diff. (ppm)	DBE
C31 H40 O7	524.2774	547.2666	547.2672	-0.60	-1.10	12.0000

--- End Of Report ---

Agilent Technologies Page 1 of 1 Printed at: 2:03 PM on: 2/25/2021

Section S33: 1D and 2D NMR spectra of compound 16

Figure S104. ^1H NMR (600 MHz, $CDCl_3$) spectrum of 16.

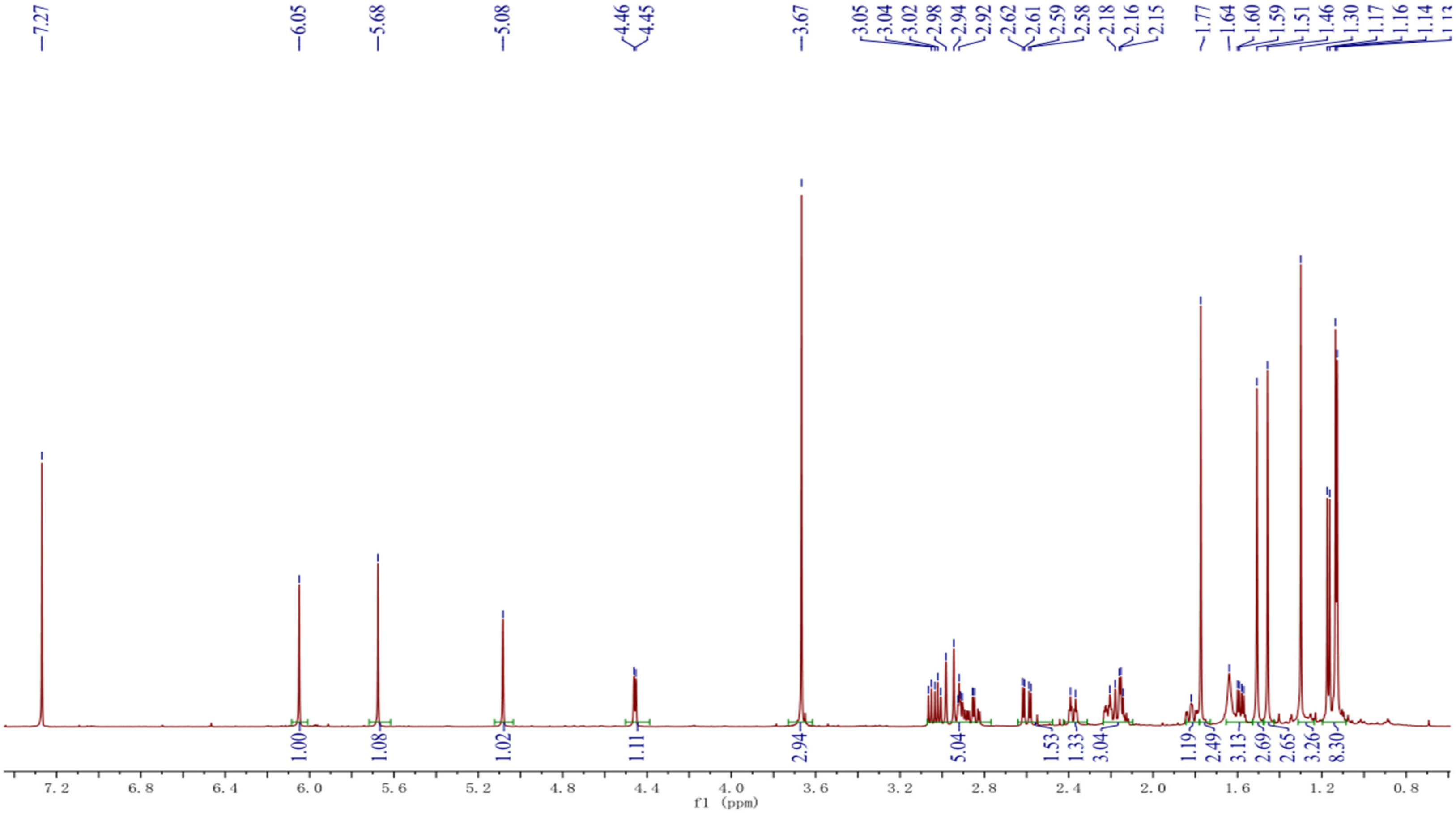

Figure S105. ^{13}**C NMR (150 MHz, CDCl$_3$) spectrum of 16.**

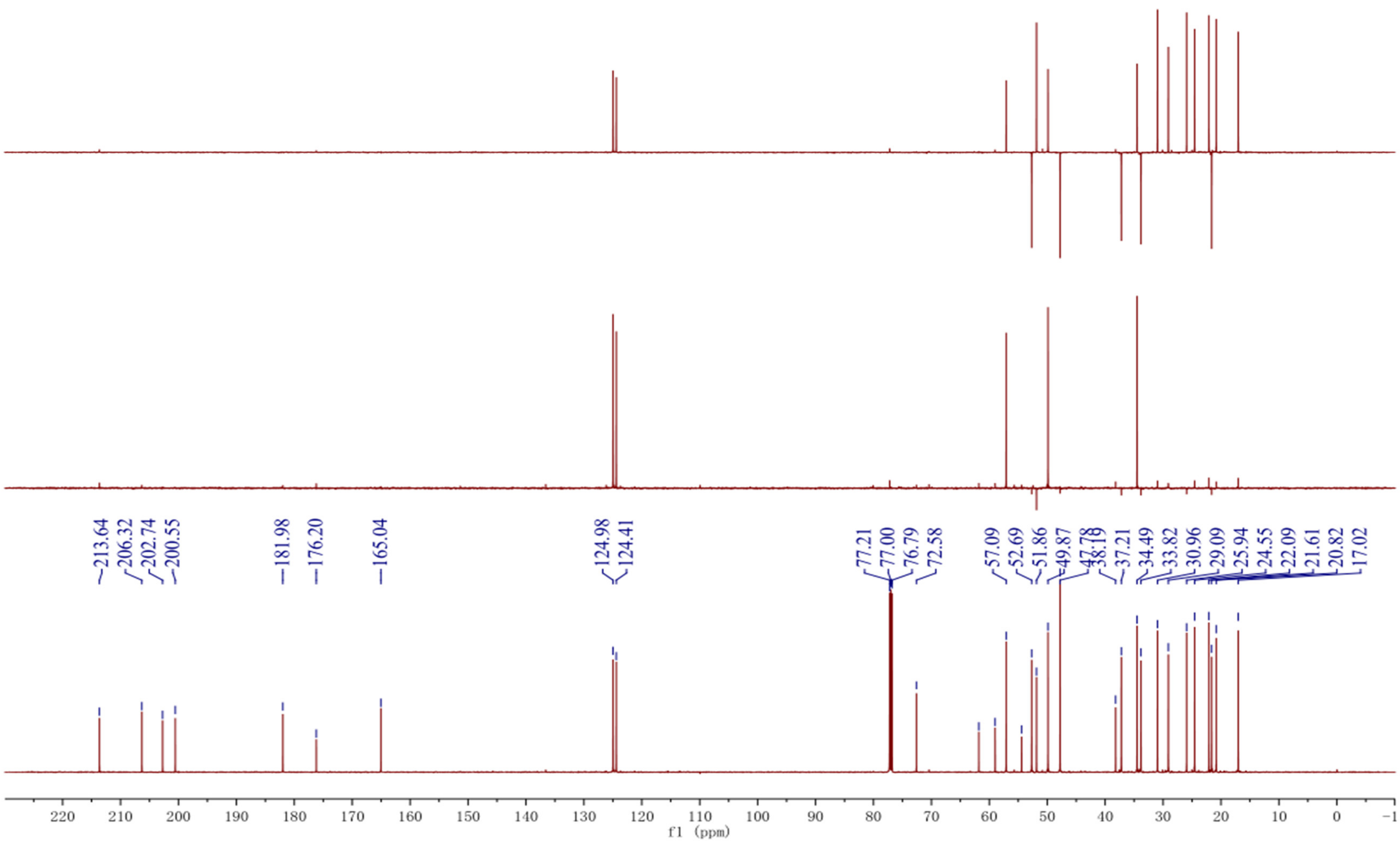

Figure S106. ^{1}H-^{1}H COSY (600 MHz, $CDCl_3$) spectrum of **16**.

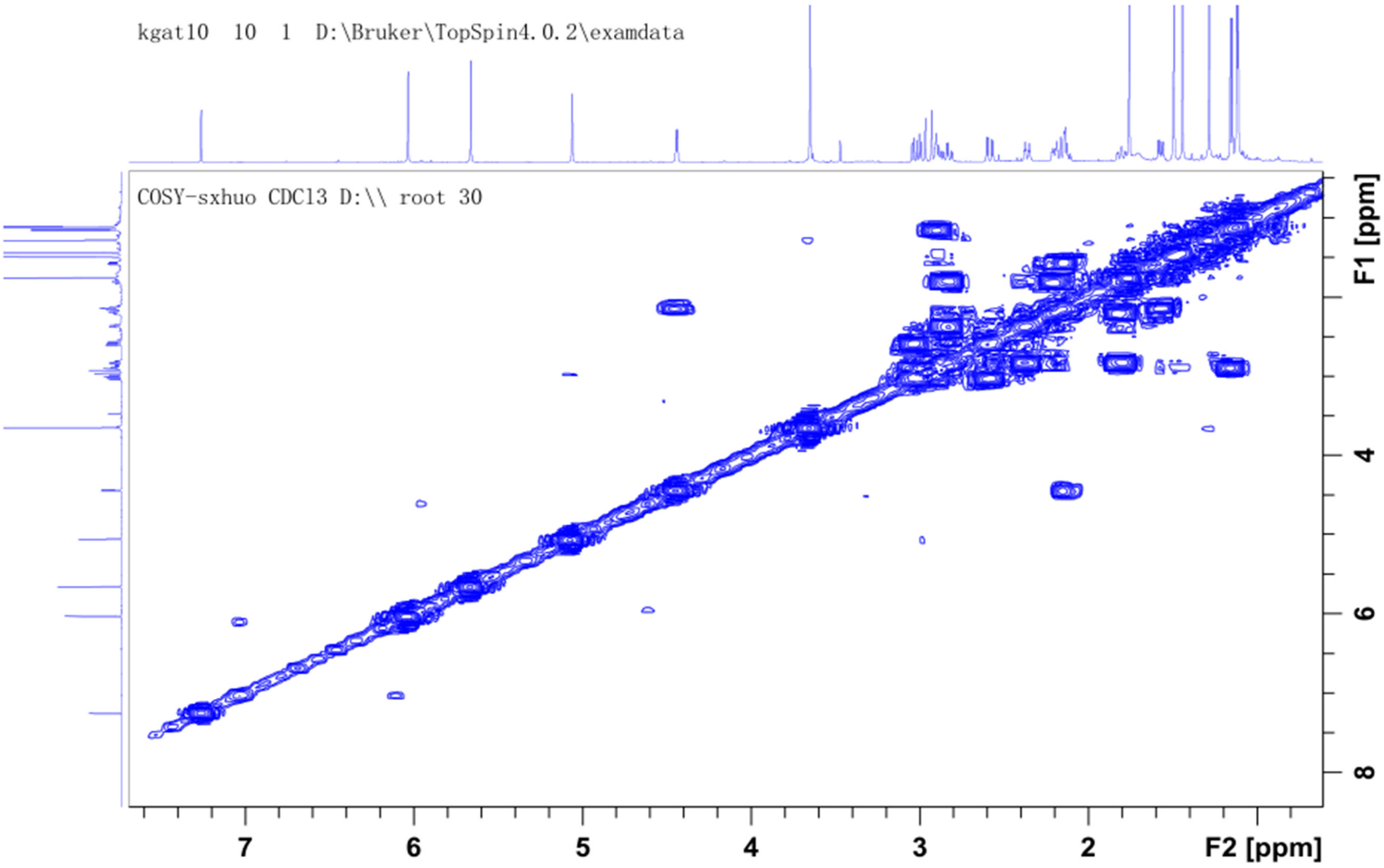

Figure S107. HSQC (600/150 MHz, $CDCl_3$) spectrum of 16.

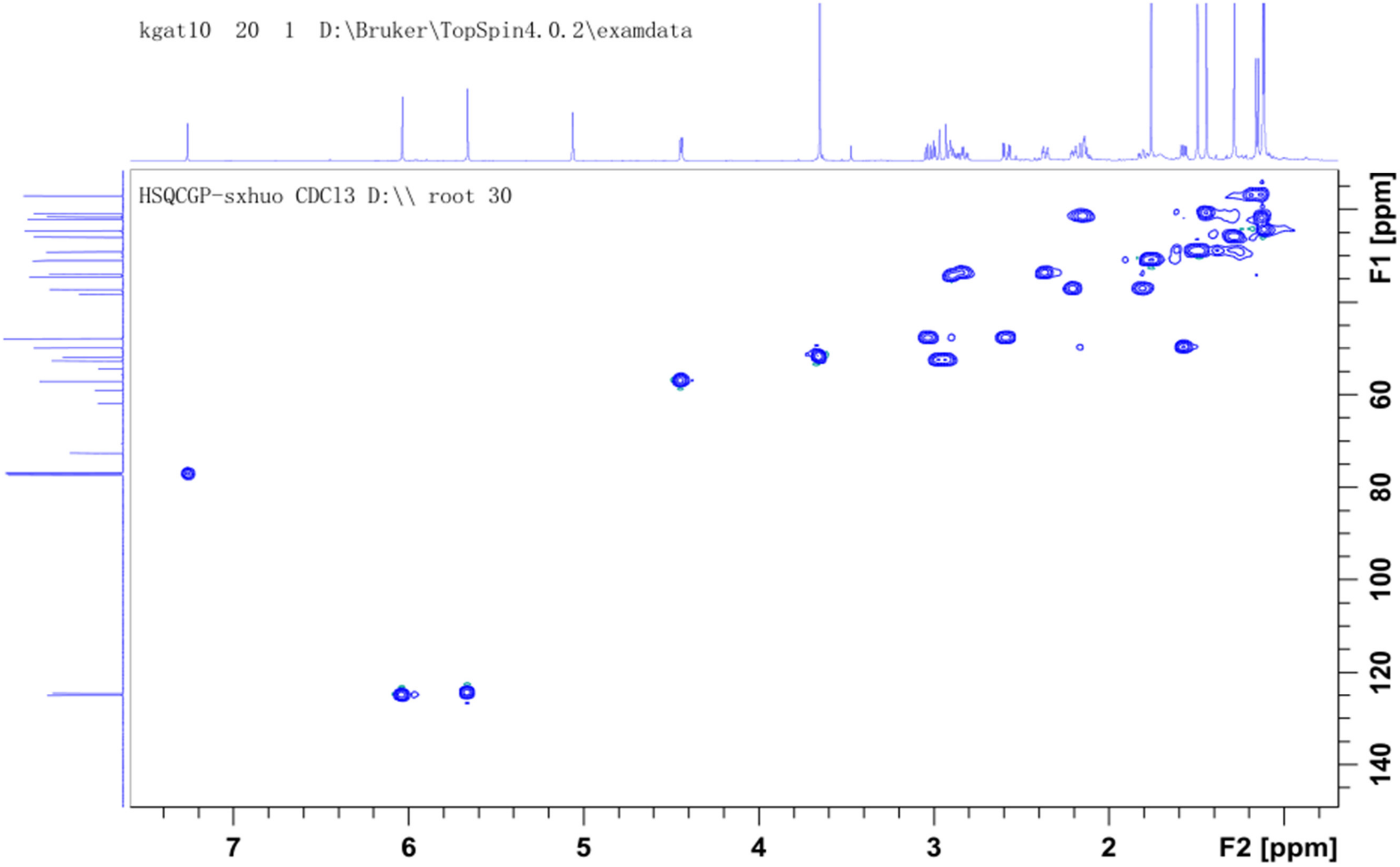

Figure S108. HMBC (600/150 MHz, $CDCl_3$) spectrum of 16.

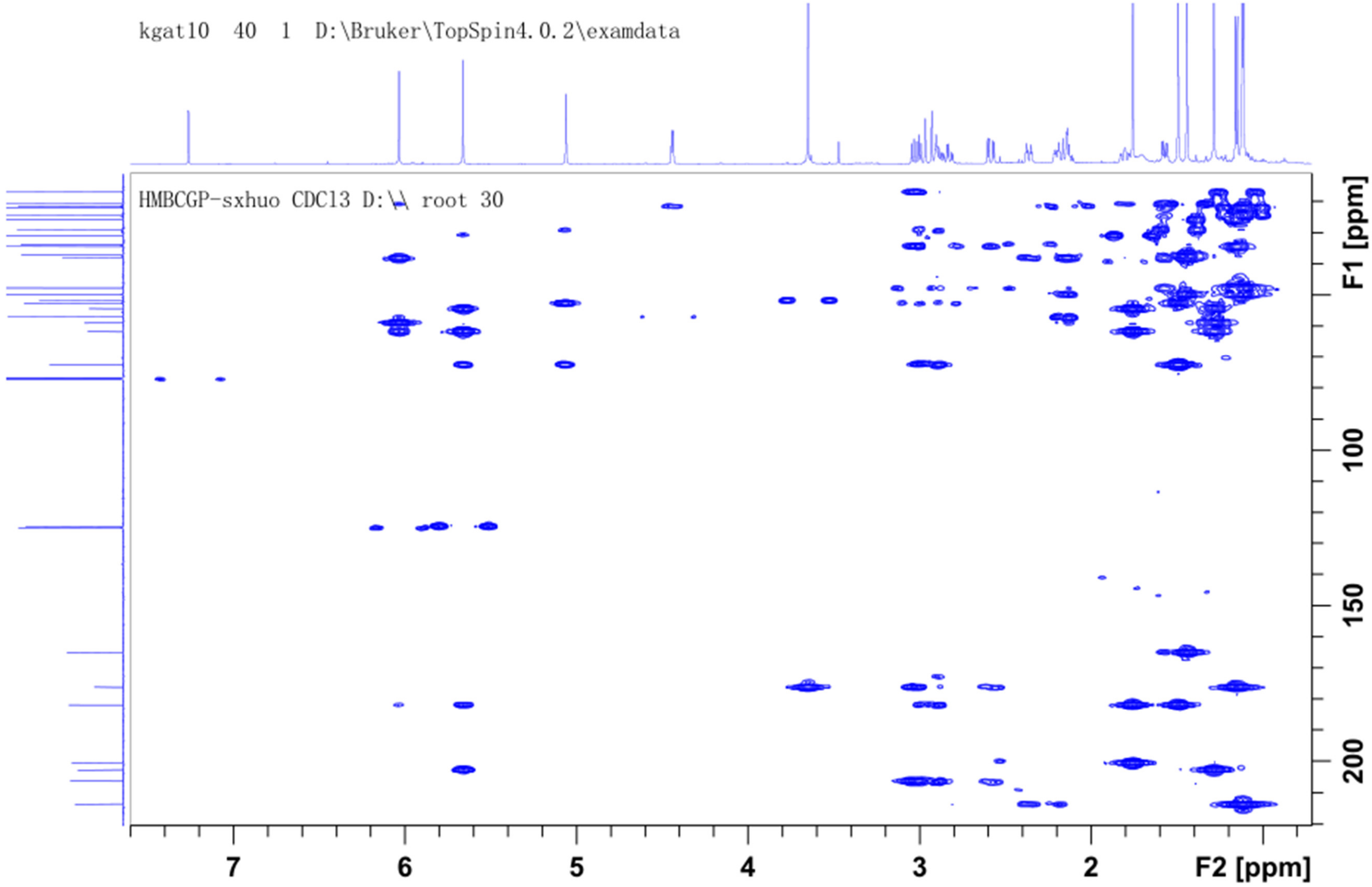

Figure S109. ROESY (600/150 MHz, $CDCl_3$) spectrum of 16.

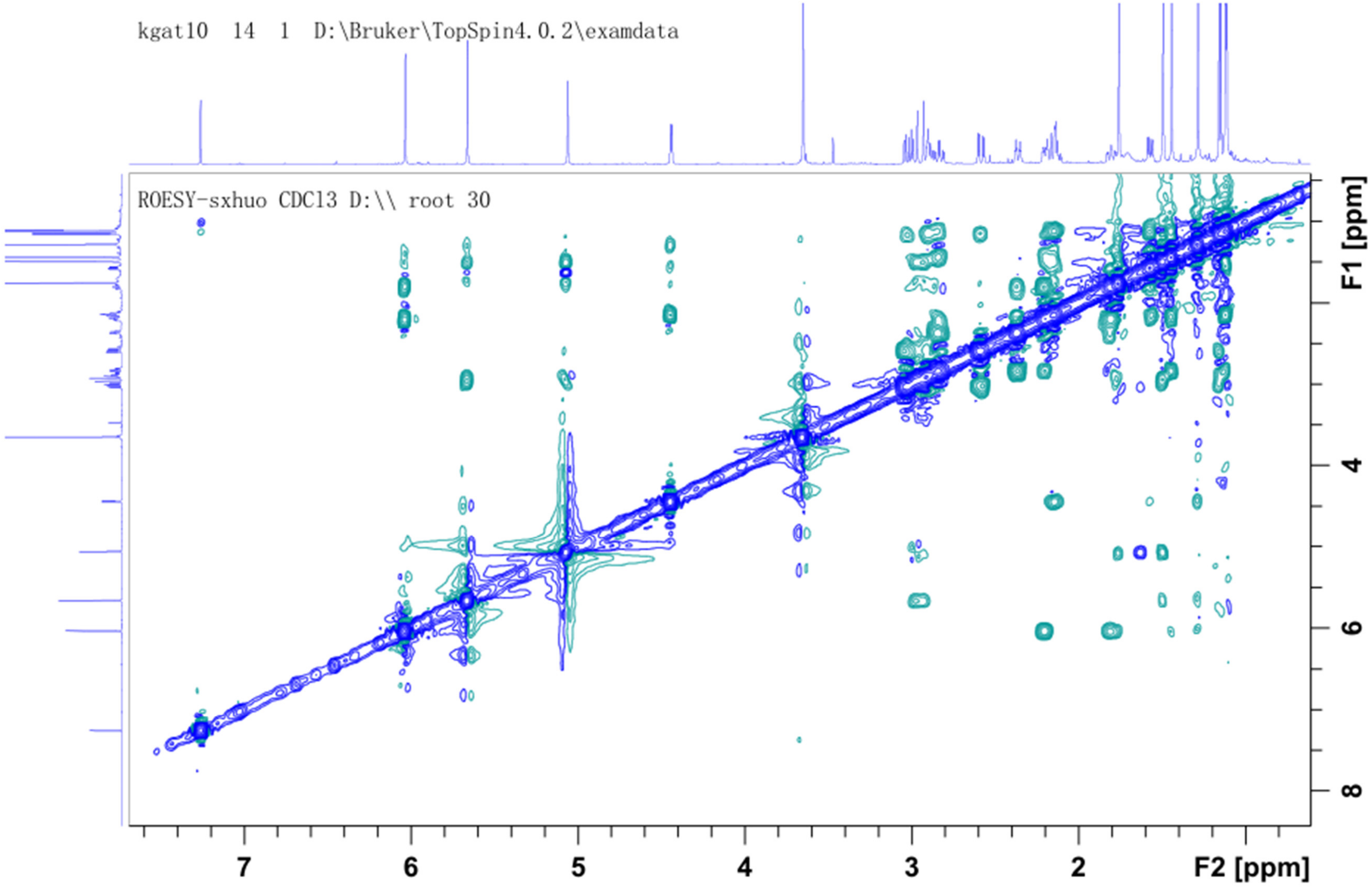

Section S34: HRESIMS spectrum of 16

Figure S110. HRESIMS spectrum of 16.

Qualitative Analysis Report

**Data Filename**	kgat10.d	**Sample Name**	kgat10
**Sample Type**	Sample	**Position**	P1-A5
**Instrument Name**	Instrument 1	**User Name**	
**Acq Method**	s.m	**Acquired Time**	2/25/2021 11:41:44 AM
**IRM Calibration Status**	Success	**DA Method**	Default.m
**Comment**			

Sample Group **Info.**
Acquisition SW Version 6200 series TOF/6500 series Q-TOF B.05.01 (B5125.2)

User Spectra

Fragmentor Voltage	Collision Energy	Ionization Mode
150	0	ESI

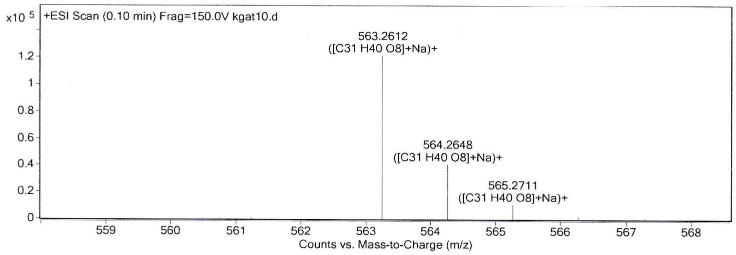

Peak List

m/z	z	Abund	Formula	Ion
102.1273	1	84281.93		
118.0856	1	18442.87		
134.0802	1	26488.62		
146.0803	1	39095.93		
150.1117	1	18621.35		
172.0936	1	38768.34		
563.2612	1	121532.69	C31 H40 O8	(M+Na)+
564.2648	1	41008.18	C31 H40 O8	(M+Na)+
579.2346	1	14703.55		
1103.5336	1	16689.55		

Formula Calculator Element Limits

Element	Min	Max
C	3	60
H	0	120
O	0	30

Formula Calculator Results

Formula	CalculatedMass	CalculatedMz	Mz	Diff. (mDa)	Diff. (ppm)	DBE
C31 H40 O8	540.2723	563.2615	563.2612	0.30	0.53	12.0000

--- End Of Report ---
